# Supplementary material for: 4-(2,5-Dimethyl-1H-pyrrol-1-yl)-N-(2,5-dioxopyrrolidin-1-yl) benzamide improves monoclonal antibody production in a Chinese hamster ovary cell culture
Source: PLoS One. 2021 Apr 22;16(4):e0250416. doi: 10.1371/journal.pone.0250416 (PMC8061942; doi:10.1371/journal.pone.0250416)
Supplement: S1 Fig — Glucose was added on days 8 and 12 to keep the concentration above 1 g/L in the fed-batch cultures. And Feed medium was added on days 4, 6, and 8. Viable cell density (A), viability (B), glucose concentration (C), lactate concentration (D), and mAb concentration (E) were measured every 2 days. The each slope of mAb concentration, consumed glucose concentration and lactate concentration to integral viable cell concentration were used to indicate cell-specific productivity (F), cell-specific glucose uptake rates (G), and cell-specific lactate production rates (H), respectively [28]. (DOCX) [file pone.0250416.s001.docx]

**H**

**F**

**G**

**E**

**B**

**A**

**D**

**C**
