# Supplementary material for: 4-(2,5-Dimethyl-1H-pyrrol-1-yl)-N-(2,5-dioxopyrrolidin-1-yl) benzamide improves monoclonal antibody production in a Chinese hamster ovary cell culture
Source: PLoS One. 2021 Apr 22;16(4):e0250416. doi: 10.1371/journal.pone.0250416 (PMC8061942; doi:10.1371/journal.pone.0250416)
Supplement: S1 Table — DMSO was used as the 0% control, and 4-phenyl butyric acid dissolved in DMSO was used as the 100% control. Validity was evaluated at the points of coefficient of variation (CV) using day 3 cell culture solution. (DOCX) [file pone.0250416.s003.docx]

**96-Well Plate Map**

**Results of the evaluation of the screening protocol**
