# Supplementary material for: 4-(2,5-Dimethyl-1H-pyrrol-1-yl)-N-(2,5-dioxopyrrolidin-1-yl) benzamide improves monoclonal antibody production in a Chinese hamster ovary cell culture
Source: PLoS One. 2021 Apr 22;16(4):e0250416. doi: 10.1371/journal.pone.0250416 (PMC8061942; doi:10.1371/journal.pone.0250416)
Supplement: Attachment — Submitted filename: PONE-D-20-26236_reviewer V1.pdf [file pone.0250416.s004.pdf]

PLOS ONE

4-(2,5-Dimethyl-1H-pyrrol-1-yl)-N-(2,5-dioxopyrrolidin-1-yl) benzamide improves monoclonal antibody production in a Chinese hamster ovary cell culture

--Manuscript Draft--

**Manuscript Number:**	PONE-D-20-26236
**Article Type:**	Research Article
**Full Title:**	4-(2,5-Dimethyl-1H-pyrrol-1-yl)-N-(2,5-dioxopyrrolidin-1-yl) benzamide improves monoclonal antibody production in a Chinese hamster ovary cell culture
**Short Title:**	A 2,5-dimethylpyrrol derivative improves monoclonal antibody productivity in rCHO cell culture
**Corresponding Author:**	Yuichi Aki<br>Daiichisankyo Co., Ltd.<br>Chiyoda-machi, Oura-gun, Gunma JAPAN
**Keywords:**	low molecular compounds; cell culture; chinese hamster ovary; galactosylation; adenosine triphosphate; monoclonal antibody production
**Abstract:**	There is a continuous demand to improve monoclonal antibody production for medication supply and medical cost reduction. For over 20 years, recombinant Chinese hamster ovary cells have been used as a host in monoclonal antibody production because of easy handling. And it is known that some chemical compounds improve monoclonal antibody production in these cell cultures. In this study, we aimed to discover new chemical compounds to improve monoclonal antibody production effectively in recombinant Chinese hamster ovary cells. During the screening performed for this study, out of 23,227 chemical compounds, 4-(2,5-dimethyl-1H-pyrrol-1-yl)-N-(2,5-dioxopyrrolidin-1-yl) benzamide was found as an enhancer of monoclonal antibody production. The compound has characteristics that suppress cell growth and increase specific glucose consumption during monoclonal antibody production. In addition, the compound also suppressed the galactosylation on a monoclonal antibody, which is a critical quality attribute of monoclonal antibodies as pharmaceuticals. Therefore, the compound might also be used as an N-linked glycan control reagent and especially as a galactosylation suppression reagent. Further, the structure-activity relationship study revealed that 2,5-dimethylpyrrole was the most effective partial structure of 4-(2,5-dimethyl-1H-pyrrol-1-yl)-N-(2,5-dioxopyrrolidin-1-yl) benzamide on monoclonal antibody production. Further structural optimization in structure-activity relationship study of 2,5-dimethylpyrrole derivatives could lead to improved production and quality control of monoclonal antibodies.
**Order of Authors:**	Yuichi Aki
	Yuta Katsumata
	Hirofumi Kakihara
	Koichi Nonaka
	Kenshu Fujiwara
**Additional Information:**	
**Question**	**Response**
**Financial Disclosure**<br><br>Enter a financial disclosure statement that describes the sources of funding for the work included in this submission. Review the submission guidelines for detailed requirements. View published research articles from *PLOS ONE* for specific examples.	The authors received no specific funding for this work.<br>As a notice, in this study, chemical compounds library was collected from Basis for Supporting Innovative Drug Discovery and Life Science Research (https://www.binds.jp/) for free. But, it didn't affect to our any study.

Powered by Editorial Manager® and ProduXion Manager® from Aries Systems Corporation

This statement is required for submission and **will appear in the published article** if the submission is accepted. Please make sure it is accurate.<br><br>**Unfunded studies**<br>Enter: *The author(s) received no specific funding for this work.*<br><br>**Funded studies**<br>Enter a statement with the following details:<br>• Initials of the authors who received each award<br>• Grant numbers awarded to each author<br>• The full name of each funder<br>• URL of each funder website<br>• Did the sponsors or funders play any role in the study design, data collection and analysis, decision to publish, or preparation of the manuscript?<br>• **NO** - Include this sentence at the end of your statement: *The funders had no role in study design, data collection and analysis, decision to publish, or preparation of the manuscript.*<br>• **YES** - Specify the role(s) played.<br><br>* typeset	
**Competing Interests**<br><br>Use the instructions below to enter a competing interest statement for this submission. On behalf of all authors, disclose any competing interests that could be perceived to bias this work—acknowledging all financial support and any other relevant financial or non-financial competing interests.<br><br>This statement **will appear in the published article** if the submission is accepted. Please make sure it is accurate. View published research articles from *PLOS ONE* for specific examples.	The authors have declared that no competing interests exist.

**NO authors have competing interests**<br><br>Enter: *The authors have declared that no competing interests exist.*<br><br>**Authors with competing interests**<br><br>Enter competing interest details beginning with this statement:<br><br>*I have read the journal's policy and the authors of this manuscript have the following competing interests: [insert competing interests here]*<br><br>* typeset	
**Ethics Statement**<br><br>Enter an ethics statement for this submission. This statement is required if the study involved:<br><br>• Human participants<br>• Human specimens or tissue<br>• Vertebrate animals or cephalopods<br>• Vertebrate embryos or tissues<br>• Field research<br><br>Write "N/A" if the submission does not require an ethics statement.<br><br>General guidance is provided below. Consult the submission guidelines for detailed instructions. **Make sure that all information entered here is included in the Methods section of the manuscript.**	N/A

Powered by Editorial Manager® and ProduXion Manager® from Aries Systems Corporation

**Format for specific study types**<br><br>**Human Subject Research (involving human participants and/or tissue)**<br>• Give the name of the institutional review board or ethics committee that approved the study<br>• Include the approval number and/or a statement indicating approval of this research<br>• Indicate the form of consent obtained (written/oral) or the reason that consent was not obtained (e.g. the data were analyzed anonymously)<br><br>**Animal Research (involving vertebrate animals, embryos or tissues)**<br>• Provide the name of the Institutional Animal Care and Use Committee (IACUC) or other relevant ethics board that reviewed the study protocol, and indicate whether they approved this research or granted a formal waiver of ethical approval<br>• Include an approval number if one was obtained<br>• If the study involved *non-human primates*, add *additional details* about animal welfare and steps taken to ameliorate suffering<br>• If anesthesia, euthanasia, or any kind of animal sacrifice is part of the study, include briefly which substances and/or methods were applied<br><br>**Field Research**<br><br>Include the following details if this study involves the collection of plant, animal, or other materials from a natural setting:<br>• Field permit number<br>• Name of the institution or relevant body that granted permission	
**Data Availability**<br><br>Authors are required to make all data underlying the findings described fully available, without restriction, and from the time of publication. PLOS allows rare exceptions to address legal and ethical concerns. See the PLOS Data Policy and FAQ for detailed information.	No - some restrictions will apply

Powered by Editorial Manager® and ProduXion Manager® from Aries Systems Corporation

A Data Availability Statement describing where the data can be found is required at submission. Your answers to this question constitute the Data Availability Statement and **will be published in the article**, if accepted.<br><br>**Important:** Stating 'data available on request from the author' is not sufficient. If your data are only available upon request, select 'No' for the first question and explain your exceptional situation in the text box.<br><br>Do the authors confirm that all data underlying the findings described in their manuscript are fully available without restriction?	
**Describe where the data may be found in full sentences. If you are copying our sample text, replace any instances of XXX with the appropriate details.**<br><br>• If the data are **held or will be held in a public repository**, include URLs, accession numbers or DOIs. If this information will only be available after acceptance, indicate this by ticking the box below. For example: *All XXX files are available from the XXX database (accession number(s) XXX, XXX.).*<br>• If the data are all contained **within the manuscript and/or Supporting Information files**, enter the following: *All relevant data are within the manuscript and its Supporting Information files.*<br>• If neither of these applies but you are able to provide **details of access elsewhere**, with or without limitations, please do so. For example:<br><br>*Data cannot be shared publicly because of [XXX]. Data are available from the XXX Institutional Data Access / Ethics Committee (contact via XXX) for researchers who meet the criteria for access to confidential data.*<br><br>*The data underlying the results presented in the study are available from (include the name of the third party*	Most chemical compounds structure cannot be shared publicly because of secret of Basis for Supporting Innovative Drug Discovery and Life Science Research [BINDS]. Authors are also limited to access the compounds without non-disclosure agreement. Only chemical compounds structure which related with our this study were available from the BINDS by contract with BINDS.

Powered by Editorial Manager® and ProduXion Manager® from Aries Systems Corporation

and contact information or URL).<br>• This text is appropriate if the data are owned by a third party and authors do not have permission to share the data.<br><br>* typeset	
Additional data availability information:	

Powered by Editorial Manager® and ProduXion Manager® from Aries Systems Corporation

Manuscript Body Click here to access/download;Manuscript;Manuscript.docx

4-(2,5-Dimethyl-1*H*-pyrrol-1-yl)-*N*-(2,5-dioxopyrrolidin-1-yl) benzamide improves monoclonal antibody production in a Chinese hamster ovary cell culture

A 2,5-dimethylpyrrol derivative improves monoclonal antibody productivity in rCHO cell culture

Yuichi Aki[1,2,¶,*], Yuta Katsumata[1,&], Hirofumi Kakihara[1,&], Koichi Nonaka[1,&], Kenshu Fujiwara[2,¶]

[1] Biologics Technology Research Laboratories, Biologics Division, Daiichi Sankyo Co., Ltd., Chiyoda-machi, Gunma, Japan

[2] Department of Life Science, Akita University, Tegata Gakuen-machi, Akita, Japan

*Corresponding author

E-mail: aki.yuichi.ha@daiichisankyo.co.jp (YA)

[¶]These authors contributed equally to this work.

[&]These authors also contributed equally to this work.

Abstract

There is a continuous demand to improve monoclonal antibody production for medication supply and medical cost reduction. For over 20 years, recombinant Chinese hamster ovary cells have been used as a host in monoclonal antibody production because of easy handling. And it is known that some chemical compounds improve monoclonal antibody production in these cell cultures. In this study, we aimed to discover new chemical compounds to improve monoclonal antibody production effectively in recombinant Chinese hamster ovary cells. During the screening performed for this study, out of 23,227 chemical compounds, 4-(2,5-dimethyl-1*H*-pyrrol-1-yl)-*N*-(2,5-dioxopyrrolidin-1-yl) benzamide was found as an enhancer of monoclonal antibody production. The compound has characteristics that suppress cell growth and increase specific glucose consumption during monoclonal antibody production. In addition, the compound also suppressed the galactosylation on a monoclonal antibody, which is a critical quality attribute of monoclonal antibodies as pharmaceuticals. Therefore, the compound might also be used as an *N*-linked glycan control reagent and especially as a galactosylation suppression reagent. Further, the structure-activity relationship study revealed that 2,5-dimethylpyrrole was the most effective partial structure of 4-(2,5-dimethyl-1*H*-pyrrol-1-yl)-*N*-(2,5-dioxopyrrolidin-1-yl) benzamide on monoclonal antibody production. Further structural optimization in structure-activity relationship study of 2,5-dimethylpyrrole derivatives could lead to improved production and quality control of monoclonal antibodies.

Introduction

Monoclonal antibody (mAb) treatment is becoming a common therapeutic treatment for cancer and autoimmune disorders. Pharmaceutical and biologics companies have strongly focused on increasing mAb supply and reducing ~~its~~ manufacturing costs for commercialization and sustainable growth. Several grams/liter of production by recombinant Chinese hamster ovary (rCHO) cells, which harbor mAb genes, has been achieved in fed-batch mode cultures through effective host-vector system development, genetically engineered host breeding, custom media development, culture parameter optimization, and so on. However, efforts have been continued to establish cost-effective manufacturing processes by reducing the use of expensive materials such as cell culture media and column resins. It is also known that adding chemical compounds in cell cultures improves mAb production. Various chemical compounds such as dimethyl sulfoxide (DMSO) [1], lithium chloride [2], butyric acid [3], valeric acid [4], valproic acid [5], phenolic antioxidants [6], thymidine [7], and CDK4/6 inhibitor [8] have the potential to enhance mAb production when added to the cell culture at an adequate concentration to control the state of rCHO cells. In addition, 3-methyladenine up-regulates the unfolded protein response pathway and improves production [9], and compound 7312, which works as a caspase inhibitor, suppresses apoptosis and improves production [10]. To pursue the possibility of the chemical compound approach, potential synthetic compounds that might enhance mAb production in rCHO cells were gathered from the Basis for Supporting Innovative Drug Discovery and Life Science Research (BINDS) program and evaluated by screening in batch mode cultures. We newly found that

4-(2,5-dimethyl-1*H*-pyrrol-1-yl)-*N*-(2,5-dioxopyrrolidin-1-yl) benzamide (MPPB), which was developed as an anti-tuberculosis therapeutic compound [11-13], stimulated mAb production in cell cultures. To reveal the characteristics of the compound, we investigated cell culture trends and the metabolism of rCHO cells under MPPB-added conditions. These studies were executed in both batch ~~mode~~ and fed-batch ~~mode~~ cultures. The results showed that MPPB improved mAb production in extended cell culture periods, suppressed cell growth, and increased glucose consumption in fed-batch mode cultures. MPPB not only improved mAb production, but it also affected the *N*-glycan profile. The structure-activity relationship of MPPB was also evaluated.

Materials and methods

Cell line and cell culture media

The tested rCHO cells, which expressed the mAb (Immunoglobulin G1), were generated from CHO-S host cells (Thermo Fisher Scientific, Waltham, MA, USA) by Daiichi Sankyo Co., Ltd. In-house chemically defined media in which L-alanyl-L-glutamine concentration was adjusted to 1 mM was used as expansion and basal media. In-house chemically defined feed media was used in the fed-batch mode cultures.

Chemical compound library

The chemical compound library was provided by the University of Tokyo, Osaka University, and Tohoku University, which are members of Basis for Supporting Innovative Drug Discovery and Life Science Research (BINDS) which is a program that supports drug discovery.

Expansion culture condition

The rCHO cells were inoculated at a target concentration of 0.3×10^6 cells/mL in 125-mL or 250-mL Erlenmeyer flasks (Corning, Corning, NY, USA) with expansion medium (50 mL in the 125-mL flasks or 100 mL in the 250-mL flasks) and incubated for 3 to 4 days in 5% CO_2-enriched air at 37.0 °C with shaking at 120 rpm (20-mm stroke length).

Screening of the chemical compound library

The rCHO cells were inoculated at a target concentration of 0.3×10^6 cells/mL in CELLSTAR 96-well suspension culture plates (Greiner Bio-One, Frickenhausen, Germany) with expansion medium

(198 µL). Rows 1 and 12 on the 96-well plates were used as controls with DMSO (2 µL). On day 0, 2 µL of each chemical compound dissolved in DMSO was added to each well. The plates were incubated at 37.0 °C in 5% CO_2-enriched air at 120 rpm (20-mm stroke length). After 3 days of culture, mAb concentrations were analyzed with Octet QKe (ForteBio, Fremont, CA, USA). The resulting mAb concentrations were evaluated as "indicators of mAb concentration," as defined by our team. An indicator of mAb concentration was calculated as: tested mAb concentration - (average mAb concentration of the control condition + 3 standard deviations). The compounds for which this indicator showed a positive value were selected as candidates for secondary screening. The validity of this screening protocol is shown in S1 Table.

Batch mode culture condition

In a secondary screening to evaluate the candidates, rCHO cells were inoculated at a target concentration of 0.3×10^6 cells/mL in 50-mL suspension culture flasks (Greiner Bio-One) with 10 mL of basal medium and incubated for 3 days in 5% CO_2-enriched air at 37.0 °C on static culture mode. All tested compounds were dissolved in DMSO at a concentration of 100 mg/mL, and 10 µL (0.1% v/v) of this solution was added to a culture on day 0. In the suspension batch mode cultures, the rCHO cell seeding flasks were incubated for 3 to 10 days in 5% CO_2-enriched air at 37.0 °C at 120 rpm (20-mm stroke length). ~~All tested compounds dissolved in DMSO were added to a culture on day 0.~~

Fed-batch mode culture condition

The rCHO cells were inoculated at a target concentration of 0.3 × 10^6 cells/mL in 125-mL Erlenmeyer flasks (Corning) with 50 mL of expansion medium and incubated until less than 70% viability was reached in 5% CO_2-enriched air at 37.0 °C at 120 rpm (20-mm stroke length). On days 4, 6, and 8, feed medium at 2% v/v against the starting volume was added. On day 0, 50 µL (0.1% v/v) of 4-(2,5-dimethyl-1*H*-pyrrol-1-yl)-*N*-(2, 5-dioxopyrrolidin-1-yl) benzamide (Abamachem Ltd., Kyiv, Ukraine) dissolved in DMSO at a concentration of 200 mg/mL was added.

Measurement of viable cell density and viability

Vi-CELL XR (Beckman Coulter, CA, USA) was used to evaluate viable cell density (VCD) and viability according to the manufacturer's instructions. All testing samples were diluted twice except for on days 0 to 4.

Metabolite analysis

Glucose consumption and lactate accumulation were analyzed by BioProfile FLEX2 (Nova Biomedical, Waltham, MA, USA) according to the manufacturer's protocol.

Concentration of mAb

Cell culture solution was filtrated with a 0.2-µm filter. The mAb concentration in the residual solution was measured by high-performance liquid chromatography (HPLC) with a Protein A affinity chromatography column, which is a PA ImmunoDetection Sensor Cartridge, with an ID of 2.1 × 30 mm (Applied Biosystems, Bedford, MA, USA).

N-linked glycan analysis

The samples purified with the spin column-based antibody purification kit (Cosmo Bio, Tokyo, Japan) according to the manufacturer's protocol were labeled with 2-AB using the EZGlyco mAb-N kit (Sumitomo Bakelite, Tokyo, Japan) for *N*-linked glycan analysis, which was performed under gradient conditions of 50 mM ammonium formate buffer (pH 4.4) on HPLC using a XBridge BEH Amide XP Column (2.5 µm, ID 4.6 × 150 mm, Waters Corporation, Milford, MA, USA) at 60 °C. The detected wavelength was 420 nm, and the flow rate was changed from 0.4 to 1.0 mL/min under gradient conditions.

Statistical analysis

At least 3 batches were executed for statistical analysis. The mean ± standard deviation (SD) and P-value were calculated using JMP statistical analysis software (SAS, NC, USA). Differences in the data were considered significant at $P < 0.05$.

Results

Initial screening of the chemical compound library

A total of 23,227 chemical compounds were evaluated by cultivation in 96-well plates as an initial screening process (Fig 1). In the rCHO cell cultures, 566 compounds showed a positive effect in terms of increased mAb production. The hit rate was 2.5%. In parallel, we investigated the commercial availability of the first screening positives before starting the second screening to obtain sufficient amounts of the positives for further study. As a result, sixty-seven chemical compounds were identified as commercially available. In addition, other five compounds were found to be ~~close~~ similar to commercially

available compounds. To conduct further screening, the original five compounds were replaced ~~to~~ the commercially available substitutes. A total of 72 samples underwent a second screening in a suspension culture.

Fig 1. Results of the first screening to evaluate the effect of the chemical compounds on mAb production. Higher mAb production is shown as a positive arbitrary unit.

Second screening of the selected compounds in a suspension culture

Seventy-two chemical compounds were further evaluated in a suspension culture for 3 days. Relative cell-specific productivity, relative mAb concentration, and viability were compared with the control condition. For most of the compounds, the relative mAb concentration was increased with the chemical compound treatment. Therefore, the chemical compounds were screened for relative mAb concentration, relative cell-specific productivity, and viability. The cutoff points for the selection of the candidate compounds were set to over 120% for relative mAb concentration, 105% for relative cell-specific productivity, and 80% for viability. The samples with the ID numbers 42, 62, and 67 met these criteria, with increases of 125 to 158% for mAb concentration and 108 to 392% for cell-specific productivity in comparison with the control condition (Fig 2).

Fig 2. Relative mAb concentration and cell-specific productivity increased with the addition of the chemical compounds. The numbers in and above the circles indicate viability and the sample's ID number, respectively.

MPPB as an effective additive of mAb production

The three selected compounds had higher ~~values in terms of~~ relative mAb concentration and relative cell-specific productivity. ID number 62 showed an especially strong effect, with a 171% increase in relative mAb concentration and a 202% increase in relative cell-specific productivity compared with the control condition (Fig 3). ID number 62 showed higher values than the other tested compounds, so we investigated it further. The selected compound was MPPB (Fig 4), a commercially available substitute for a first-screening-hit-compound supplied from the chemical compound library of the University of Tokyo. MPPB was originally developed as an anti-tuberculosis therapeutic compound [11-13].

Fig 3. The effect of the three candidate compounds on relative mAb concentration and relative cell-specific productivity. All cell cultures were executed three times and evaluated on day 3. Each value of relative mAb production (**A**) and relative cell-specific productivity (**B**) were compared statistically.

Fig 4. The structure of 4-(2,5-dimethyl-1*H*-pyrrol-1-yl)-*N*-(2,5-dioxopyrrolidin-1-yl) benzamide (MPPB).

Effect of MPPB on the culture profile in the batch mode cultures

The effect of MPPB on the culture profile was tested in the batch mode cultures, as shown in Fig 5. MPPB suppressed cell growth depending on the concentration of MPPB added (Fig 5A). Viability was slightly decreased at a MPPB concentration of 1.28 mM (Fig 5B). Cell-specific productivity was increased by an addition of MPPB with a concentration over 0.32 mM (Fig 5C). From these results, MPPB concentrations of 0.32 to 0.64 mM were used in following experiments.

Fig 5. MPPB suppresses cell growth and increases cell-specific productivity. The tested MPPB concentration is indicated in the figure. In these tests, VCD (**A**), Viability (**B**), and cell-specific productivity (**C**) were evaluated on day 4.

MPPB was further evaluated in the batch mode cultures. The maximum VCD was reduced from 16.6×10^6 cells/mL to 8.0×10^6 cells/mL with MPPB treatment (Fig 6A). On the other hand, there was longer viability under the MPPB-added condition (Fig 6B). The mAb concentration under the MPPB-added condition was almost the same value as the control condition at each harvest time point (Fig 6C). The MPPB addition increased the cell-specific productivity from 4.2 pg/cell/day to 7.9 pg/cell/day (Fig 6D).

Fig 6. Cell culture profile and mAb production in rCHO cells treated with MPPB. MPPB at 0.64 mM was added in the batch mode cultures on day 0. VCD (**A**) and viability (**B**) were measured every 2 days. Cell culture was continued until viability below 70% was reached. The concentration of mAb (**C**) and integral viable cell concentration were used to calculate mAb production (**D**) [14].

From a spent media analysis, glucose consumption and lactate accumulation showed no differences between the conditions with and without the addition of MPPB (Figs 7A and 7B). However, specific glucose consumption increased from 0.49 to 1.1 pmol/cell/day with MPPB treatment (Fig 7C). Glucose consumption under the MPPB-added condition was 2.1-fold higher than that under the control condition. Specific lactate accumulation slightly increased from 0.22 to 0.37 pmol/cell/day under the MPPB-added condition (Fig 7D).

Fig 7. The effect of MPPB on the metabolite profile in the batch mode cultures. The spent media analysis of glucose concentration (**A**) and lactate concentration (**B**) was conducted every 2 days starting on day 2. Specific glucose consumption (**C**) and specific lactate accumulation (**D**) were indicated as cumulative glucose consumption and cumulative lactate accumulation to integral viable cell concentration [14, 15].

Effect of MPPB on the culture profile in the fed-batch mode cultures

Further evaluation of the MPPB-added condition was continued in the fed-batch mode cultures. The maximum VCD under the MPPB-added condition was reduced from 21.2 × 10^6 cells/mL in the control condition to 14.0 × 10^6 cells/mL (Fig 8A). The VCD and viability also decreased after day 8 in the control condition (Figs 8A and 8B). However, they were kept until day 12 under the MPPB-added condition. Despite suppression of the VCD, the final mAb concentration under the MPPB-added condition reached 1,098 mg/L ~~and became~~ 1.5 times higher than that under the control condition (Fig 8C). The cell-specific productivity under the MPPB-added condition also became 1.5-fold higher than that under the control condition (Fig 8D). The cell-specific productivity under the control and MPPB-added conditions was 7.1 and 11 pg/cell/day, respectively.

Fig 8. Cell culture profile and mAb production in the fed-batch mode cultures under the MPPB-added condition. MPPB at 0.64 mM was added to a culture on day 0, and 2% (v/v) feed medium was added on days 2, 4, and 6. VCD (**A**) and viability (**B**) were measured every 2 days. The cell culture was continued until viability below 70% was reached. The slope of the mAb concentration (**C**) and integral viable cell concentration were used as cell-specific productivity (**D**) [14].

The glucose consumption rate under the control condition was faster than that under the MPPB-added condition, as shown in Fig 9A. Residual glucose was fully consumed by day 10. On the other hand, glucose under the MPPB-added condition was retained until day 14. The lactate concentration was kept under 0.8 g/L, and no difference was noted in both conditions (Fig 9B). The specific glucose consumption rates were 0.63 and 0.74 pmol/cell/day under the control and MPPB-added conditions, respectively (Fig 9C).

Fig 9. Metabolite analysis of the fed-batch mode cultures under the MPPB-added condition. Glucose concentration (**A**) and lactate accumulation (**B**) were measured before adding the feed medium. Glucose concentration after adding the feed medium was calculated using the added feed medium amount. Specific glucose consumption (**C**) was indicated as a slope of cumulative glucose consumption against integral viable cell concentration [14, 15].

In addition, major *N*-linked glycans (G0F, G1F, G2F, and M5) on mAb were investigated on day 14 under the MPPB-added condition. G0F was the major *N*-linked glycan, and G1F was decreased from 24.5 to 14.8% under the MPPB-added condition, although G2F and M5 ratios were not changed, as shown in Fig 10.

Fig 10. MPPB suppresses galactosylation of mAb. Each value was calculated as the percentage of total *N*-linked glycans. The ratio was statically analyzed.

Structure-activity relationship of MPPB

The MPPB structure is divided into five chemical components, as follows: *N*-(2,5-dioxopyrrolidin-1-yl) benzamide; 4-(2,5-dimethyl-1*H*-pyrrol-1-yl) benzamide; succinimide; 4-aminobenzamide; and 2,5-dimethylpyrrole (Fig 11).

Fig 11. Five chemical components derived from MPPB.

The activity of the five chemical components derived from MPPB was identified and compared with that of MPPB in terms of cell-specific productivity (Fig 12). 4-(2,5-Dimethyl-1*H*-pyrrol-1-yl) benzamide and 2,5-dimethylpyrrole increased cell-specific productivity. Further, the 2,5-dimethylpyrrole-added condition had a 2.2-fold higher cell-specific productivity than the control condition.

Fig 12. Evaluation of the effect of the five chemical components derived from MPPB on cell-specific productivity. Each chemical compound was added on day 0 and evaluated on day 3. The tested concentration of each chemical compound was 0.32 mM.

Lane 1: dimethyl sulfoxide (control); lane 2: MPPB; lane 3: *N*-(2,5-dioxopyrrolidin-1-yl) benzamide; lane 4: 4-(2,5-dimethyl-1*H*-pyrrol-1-yl) benzamide; lane 5: succinimide; lane 6: 4-aminobenzamide; lane 7: 2,5-dimethylpyrrole.

Further evaluation was conducted to identify the effect of pyrrole derivatives on viability and cell-specific productivity in the batch mode cultures (Table 1). Cell-specific productivity with alkyl pyrrole derivatives (**2**, **9-14**) was increased up to 1.4- to 7.8-fold higher than that with the control condition, although pyrrole (**7**) and 1-alkyl pyrroles (**1**, **8**) did not show any activity. On the other hand, viability decreased below 50% with these alkyl pyrrole derivatives (**2**, **9-14**), except for 2,5-dimethylpyrrole (**13**). 2,5-Dimethylpyrrolidine (**6**), which has a reduced framework of 2,5-dimethylpyrrole (**13**), also did not affect the cell culture. Thus, 2,5-Dimethyl pyrrole (**13**) was found to be the most effective chemical structure to increase mAb production in a tested rCHO cell culture.

Table 1. Effect of pyrrole derivatives on viability and relative specific productivity in rCHO cell cultures.

Compound No.	Compound name	Viability (%)	Relative specific production rate
**-**	Dimethyl sulfoxide (control)	99.4 (0.4)	1.0 (0.0)
**-**	MPPB	99.2 (0.5)	1.4 (0.2)
**1**	1-(2-Hydroxyethyl)pyrrole	99.6 (0.2)	1.0 (0.1)
**2**	2-Ethylpyrrole	41.2 (2.5)	1.4 (0.1)
**3**	Pyrrole-2-carboxaldehyde	99.3 (0.2)	1.0 (0.1)
**4**	Pyrrole-2-carboxylic acid	99.5 (0.2)	1.0 (0.1)
**5**	Pyrrolidine	98.9 (1.4)	1.0 (0.0)
**6**	2,5-Dimethylpyrrolidine	99.0 (1.0)	1.0 (0.0)
**7**	Pyrrole	99.3 (0.7)	1.0 (0.1)
**8**	1-Methylpyrrole	99.4 (0.1)	1.0 (0.1)
**9**	2-Methylpyrrole	50.2 (0.2)	3.0 (0.2)
**10**	3-Methylpyrrole	34.4 (2.0)	4.0 (0.2)
**11**	2,3-Dimethylpyrrole	2.6 (1.7)	7.8 (0.4)
**12**	2,4-Dimethylpyrrole	30.7 (3.4)	4.8 (0.3)
**13**	2,5-Dimethylpyrrole	97.9 (0.6)	2.2 (0.0)
**14**	3,4-Dimethylpyrrole	4.0 (1.2)	7.4 (0.3)

Each pyrrole derivative was added on day 0 and evaluated on day 3. The tested concentration of each pyrrole derivative was 0.32 mM. Standard deviation was indicated in the blanket.

Discussion

In this study, we screened chemical compounds to find novel additives that improve mAb production in rCHO cells and thereby promote pharmaceutical supply (Figs 1-3). MPPB had 1.5-fold higher mAb production than the control conditions in the fed-batch mode cultures. This is the first study in which MPPB was applied in rCHO cell cultures to improve mAb production.

Our study revealed two key factors for the improvement of mAb production by MPPB. One is the extension of cell culture period (Figs 6A and 8A), and the other is the increase of cell-specific productivity (Figs 6D and 8D). It is likely that these two factors explain the increased mAb production in the MPPB-added condition despite lower cell concentrations compared to the control condition.

The reason why MPPB extended cell culture periods is unclear. MPPB might keep glucose concentrations high, leading to extended cell culture periods (Figs 8A and 9A). However, although the remaining glucose concentration was almost similar with and without the addition of MPPB in the batch mode cultures, the viability remained high under the MPPB-added condition (Figs 6B and 7A). ~~Additionally,~~ once ~~we kept a~~ glucose concentration above 1 g/L in the fed-batch mode cultures (S1 Fig), the viability under the control condition was lower than that under the MPPB-added condition. These results indicate that MPPB worked on any intracellular functions that keep cells alive or suppress the exhaustion of unidentified medium ingredients to maintain viability.

The cause of increased cell-specific productivity, however, is clearer. In our study, MPPB treatment had two important effects to increase cell-specific productivity. One was the suppression of cell growth (Figs 6A and 8A), and the other was the acceleration of specific glucose consumption. Both the effects would control the amount of intracellular adenosine triphosphates (ATPs). As reported by Mulukutla et al., growing cells require more ATPs than quiescent cells [16]. Increased specific glucose consumption (Figs 7C and 9C) would lead to increased amounts of intracellular ATP since glucose is commonly metabolized to ATP in the tricarboxylic acid cycle [17, 18]. Becker et al. [19] and Hara and

Kondo [20] have reported that the energy available in the form of intracellular ATP is crucial for mAb production. Accordingly, it may be hypothesized that rCHO cells increased intracellular ATP amounts by suppressing cell growth and increasing glucose consumption, thereby increasing cell-specific productivity, as expected. To test this hypothesis, we preliminary investigated intracellular ATP amounts. We found that intracellular ATP amounts were highly enhanced (6.4-fold) under the MPPB-added condition (S2 Fig). Therefore, we predicted that the increased cell-specific productivity under the MPPB-added condition might occur due to the increasing amounts of intracellular ATP. Our results showed that a chemical compound such as MPPB, which suppressed cell growth and increased intracellular ATP amounts, may improve mAb production in rCHO cell cultures.

Furthermore, we found that MPPB treatment suppressed galactosylation in *N*-linked glycans on the expressed mAb (Fig 10). The galactosylation ratio in *N*-linked glycans on mAb is one of the critical quality attributes of mAb in terms of pharmaceutical development, because the galactosylation profile affects complement-dependent cytotoxicity [21, 22] and antibody-dependent cellular cytotoxicity [23, 24, 25]. Therefore, to adjust mAb activity and maintain consistent quality, galactosylation is often controlled during pharmaceutical process research and development. Some media additives are known to affect the galactosylation of mAb in rCHO cells. For example, CDK4/6 inhibitor, galactose, manganese, and uridine encourage galactosylation [8, 26, 27], while zinc and ammonia suppress galactosylation [28, 29]. However, ammonia is harmful to rCHO cells [30]. The number of effective galactsylation suppressant is small, and, therefore, the suppression of galactosylation by media additives is not well understood. Our findings that

MPPB suppressed the galactosylation may offer a new option to control the quality of mAb by the supression of garactosylation in the *N*-linked glycans of mAb.

Finally, the structure-activity relationship study revealed that the most critical chemical portion of MPPB to maintain viability and improve cell-specific productivity of mAb in rCHO cells is 2,5-dimethyl pyrrole (Fig 12, Table 1). Since 2,5-dimethyl pyrrolidine having a reduced framework of 2,5-dimethyl pyrrole showed no effect, the importance of the heteroaromatic framework of 2,5-dimethyl pyrrole for both the activities was suggested. The 2,5-dialkyl substitution of pyrrole was also indispensable. The viability was ~~extremely~~ decreased in the cases of other mono- or bis-alkyl-substituted pyrroles. Furthermore, non-alkylated or *N*-substituted pyrroles showed no effect on both the viability and the cell-specific productivity. Thus, the 2,5-dialkyl-substituted pyrrole framework was found to be effective on maintaining viability and improving cell-specific productivity of mAb in rCHO cells during the structure-activity relationship study. The mechanism of the action of MPPB and 2,5-dimethyl pyrrole in rCHO cell cultures is currently being considered for future application studies.

Acknowledgments

This research was supported by the Platform Project for Supporting Drug Discovery and Life Science Research (Basis for Supporting Innovative Drug Discovery and Life Science Research [BINDS]) from Japan Agency for Medical Research and Development (AMED) under grant numbers JP19am0101086, JP19am0101084, and JP19am0101095 (support numbers 1009, 0847, and 1011).

References

1. Liu CH, Chen LH. Promotion of recombinant macrophage colony stimulating factor production by dimethyl sulfoxide addition in Chinese hamster ovary cells. J Biosci Bioeng. 2007 Jan;103(1):45-9. doi: 10.1263/jbb.103.45
2. Ha TK, Kim YG, Lee GM. Effect of lithium chloride on the production and sialylation of Fc-fusion protein in Chinese hamster ovary cell culture. Appl Microbiol Biotechnol. 2014;98:9239-48. doi: 10.1007/s00253-014-6012-0
3. Jiang Z, Sharfstein ST. Sodium butyrate stimulates monoclonal antibody over-expression in CHO cells by improving gene accessibility. Biotechnol Bioeng. 2008 May 1;100(1):189-94. doi: 10.1002/bit.21726
4. Park JH, Noh SM, Woo JR, Kim JW, Lee GM. Valeric acid induces cell cycle arrest at G1 phase in CHO cell cultures and improves recombinant antibody productivity. Biotechnol J. 2016 Mar;11(4):487-96. doi: 10.1002/biot.201500327
5. Yang WC, Lu J, Nguyen NB, Zhang A, Healy NV, Kshirsagar R, et al. Addition of valproic acid to CHO cell fed-batch cultures improves monoclonal antibody titers. Mol Biotechnol. 2014 May;56(5):421-8. doi: 10.1007/s12033-013-9725-x
6. Toronjo-Urquiza L, James DC, Nagy T, Falconer RJ. Screening naturally occurring phenolic antioxidants for their suitability as additives to CHO cell culture media used to produce

monoclonal antibodies. Antioxidants (Basel). 2019 Jun 3;8(6):159. doi: 10.3390/antiox8060159

7. Chen F, Fan L, Wang J, Zhou Y, Ye Z, Zhao L, et al. Insight into the roles of hypoxanthine and thymidine on cultivating antibody-producing CHO cells: cell growth, antibody production and long-term stability. Appl Microbiol Biotechnol. 2012 Jan;93(1):169-78. doi: 10.1007/s00253-011-3484-z
8. Du Z, Treiber D, McCarter JD, Fomina-Yadlin D, Saleem RA, McCoy RE, et al. Use of a small molecule cell cycle inhibitor to control cell growth and improve specific productivity and product quality of recombinant proteins in CHO cell cultures. Biotechnol Bioeng. 2015 Jan;112(1):141-55. doi: 10.1002/bit.25332
9. Baek E, Lee JS, Lee GM. Untangling the mechanism of 3-methyladenine in enhancing the specific productivity: transcriptome analysis of recombinant Chinese hamster ovary cells treated with 3-methyladenine. Biotechnol Bioeng. 2018 Sep;115(9):2243-54. doi: 10.1002/bit.26777
10. Arden N, Ahn SH, Vaz W, Rhodes M, Hancock C, Abitorabi MA, et al. Chemical caspase inhibitors enhance cell culture viabilities and protein titer. Biotechnol Prog. 2007;23:506-11. doi: 10.1021/bp060222m
11. Joshi SD, More Y, Vagdevi HM, Vaidya VP, Gadaginamath GS, Kulkarni VH, et al. Synthesis of new 4-(2,5-dimethylpyrrol-1-yl)/4-pyrrol-1-yl benzoic acid hydrazide analogs

and some derived oxadiazole, triazole and pyrrole ring systems: a novel class of potential antibacterial, antifungal and antitubercular agents. Med Chem Res. 2013;22:1073-89. doi: 10.1007/s00044-012-0112-0

12. Joshi SD, More UA, Dixit SR, Korat HH, Aminabhavi TM, Badiger AM, et al. Synthesis, characterization, biological activity, and 3D-QSAR studies on some novel class of pyrrole derivatives as antitubercular agents. Med Chem Res. 2014;23:1123-47. doi: 10.1007/s00044-013-0709-y

13. Zhao T, Zhao Z, Lu F, Chang S, Zhang J, Pang J, et al. Two- and three-dimensional QSAR studies on hURAT1 inhibitors with flexible linkers: topomer CoMFA and HQSAR. Mol Divers. 2020;24:141-54. doi: 10.1007/s11030-019-09936-5

14. Renard JM, Spagnoli R, Mazier C, Salles MF, Mandine E. Evidence that monoclonal antibody production kinetics is related to the integral of the viable cells curve in batch systems. Biotechnol Lett. 1988;10(2):91-6. doi; 10.1007/BF01024632

15. Chen F, Ye Z, Zhao L, Liu X, Fan L, Tan WS. Correlation of antibody production rate with glucose and lactate metabolism in Chinese hamster ovary cells. Biotechnol Lett. 2012;34:425-32. doi.10.1007/s10529-011-0798-y

16. Mulukutla BC, Khan S, Lange A, Hu WS. Glucose metabolism in mammalian cell culture: new insights for tweaking vintage pathways. Trends Biotechnol. 2010 Sep;28(9):476-84. doi: 10.1016/j.tibtech.2010.06.005

17. Lowenstein J. Methods in enzymology, volume 13: Citric acid cycle. 1st ed. Boston: Academic Press; 1969.

18. Kay J, Weitzman PD. Krebs' citric acid cycle: half a century and still turning. 1st ed. London: Biochemical Society; 1987.

19. Becker M, Junghans L, Teleki A, Bechmann J, Takors R. The less the better: how suppressed base addition boosts production of monoclonal antibodies with Chinese hamster ovary cells. Front Bioeng Biotechnol. 2019;7:76. doi: 10.3389/fbioe.2019.00076

20. Hara KY, Kondo A. ATP regulation in bioproduction. Microb Cell Fact. 2015;14:198. doi: 10.1186/s12934-015-0390-6

21. Hodoniczky J, Zheng YZ, James DC. Control of recombinant monoclonal antibody effector functions by Fc N-glycan remodeling in vitro. Biotechnol Prog. 2005;21:1644-52. doi: 10.1021/bp050228w

22. Liu L. Antibody glycosylation and its impact on the pharmacokinetics and pharmacodynamics of monoclonal antibodies and Fc-fusion proteins. J Pharm Sci. 2015;104(6):1866-84. doi: 10.1002/jps.24444

23. Simonov V, Ivanov S, Smolov M, Abbasova S, Piskunov A, Poteryaev D. Control of therapeutic IgG antibodies galactosylation during cultivation process and its impact on IgG1/FcγR interaction and ADCC activity. Biologicals. 2019 Mar;58:16-21. doi: 10.1016/j.biologicals.2019.01.002

24. Thomann M, Reckermann K, Reusch D, Prasser J, Tejada ML. Fc-galactosylation modulates antibody-dependent cellular cytotoxicity of therapeutic antibodies. Mol Immunol. 2016 May;73:69-75. doi: 10.1016/j.molimm.2016.03.002

25. Thomann M, Schlothauer T, Dashivets T, Malik S, Avenal C, Bulau P, et al. In vitro glycoengineering of IgG1 and its effect on Fc receptor binding and ADCC activity. PLoS One. 2015;10(8):e0134949. doi: 10.1371/journal.pone.0134949

26. Gramer MJ, Eckblad JJ, Donahue R, Brown J, Shultz C, Vickerman K, et al. Modulation of antibody galactosylation through feeding of uridine, manganese chloride, and galactose. Biotechnol Bioeng. 2011 Jul;108(7):1591-602. doi: 10.1002/bit.23075

27. Williamson J, Miller J, McLaughlin J, Combs R, Chu C. Scale-dependent manganese leaching from stainless steel impacts terminal galactosylation in monoclonal antibodies. Biotechnol Prog. 2018 Oct;34(5):1290-7. doi: 10.1002/btpr.2662

28. Prabhu A, Gadre R, Gadgil M. Zinc supplementation decreases galactosylation of recombinant IgG in CHO cells. Appl Microbiol Biotechnol. 2018;102:5989-99. doi: 10.1007/s00253-018-9064-8

29. Chen P, Harcum SW. Effects of elevated ammonium on glycosylation gene expression in CHO cells. Metabol Eng. 2006 Mar;8(2):123-32. doi: 10.1016/j.ymben.2005.10.002

30. Chen P, Harcum SW. Effects of amino acid additions on ammonium stressed CHO cells. J Biotechnol. 2005 May 25;117(3):277-86. doi: 10.1016/j.jbiotec.2005.02.003

Supporting information

S1 Fig. The fed-batch culture profile in the retained glucose condition. Glucose was added on days 8 and 12 to keep the concentration above 1 g/L in the fed-batch cultures. And Feed was added on days 4, 6, and 8. VCD (**A**), Viability (**B**), Glucose concentration (**C**), Lactate Concentration (**D**) were measured every 2 days.

S2 Fig. MPPB affects intracellular ATP amounts in rCHO cells. The tested MPPB concentration was 0.64 mM in the culture solution, and the ATP amounts were analyzed by sampling day-4 cells in the batch mode cell cultures.

S1 Table. The validity of the first screening condition. DMSO was used as the 0% control, and 4-phenyl butyric acid dissolved in DMSO was used as the 100% control. Validity was evaluated at the points of coefficient of variation (CV) using day 3 cell culture solution.

Click here to access/download;Figure;Fig 1.tif

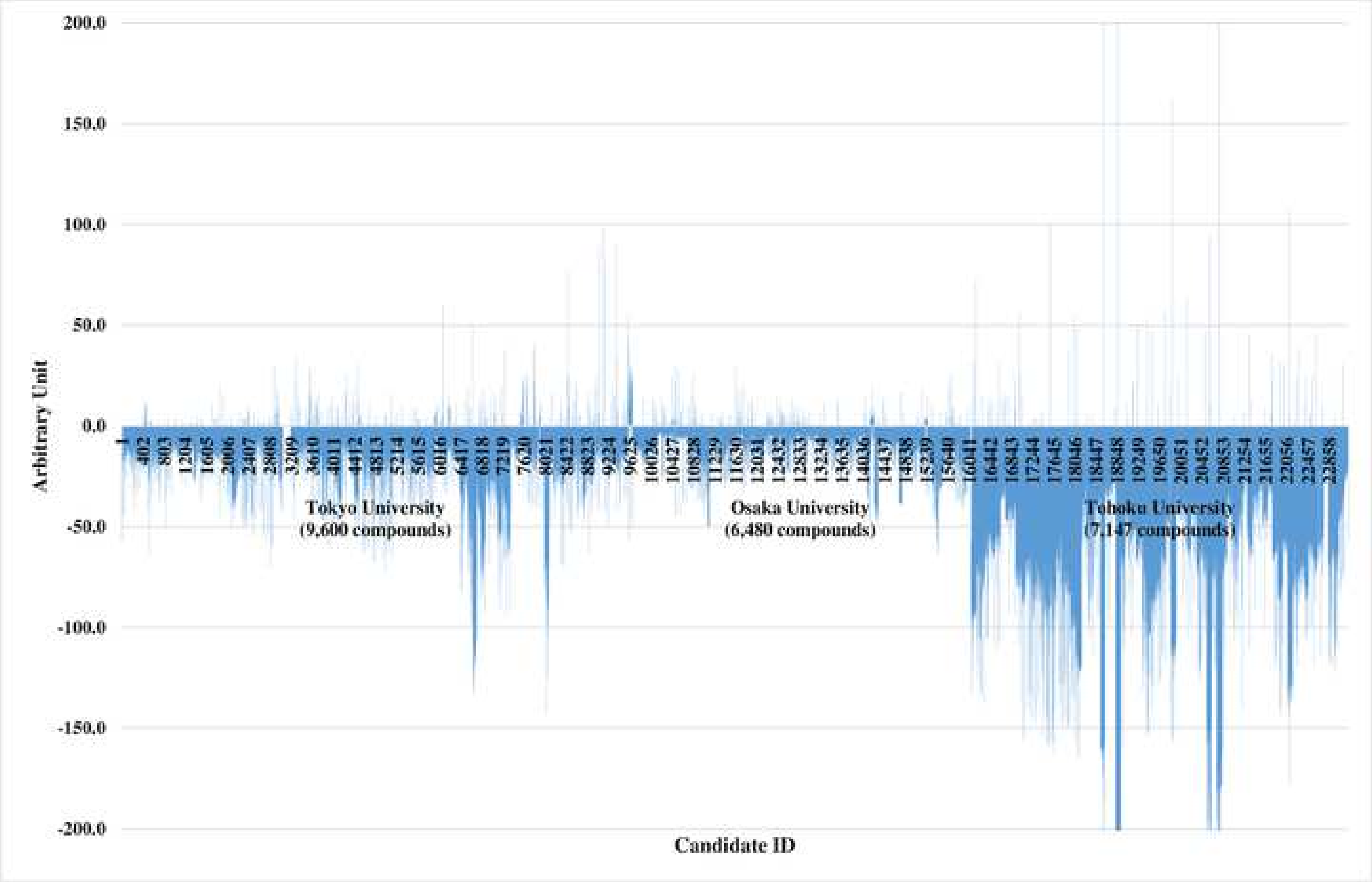

Click here to access/download;Figure;Fig 2.tif

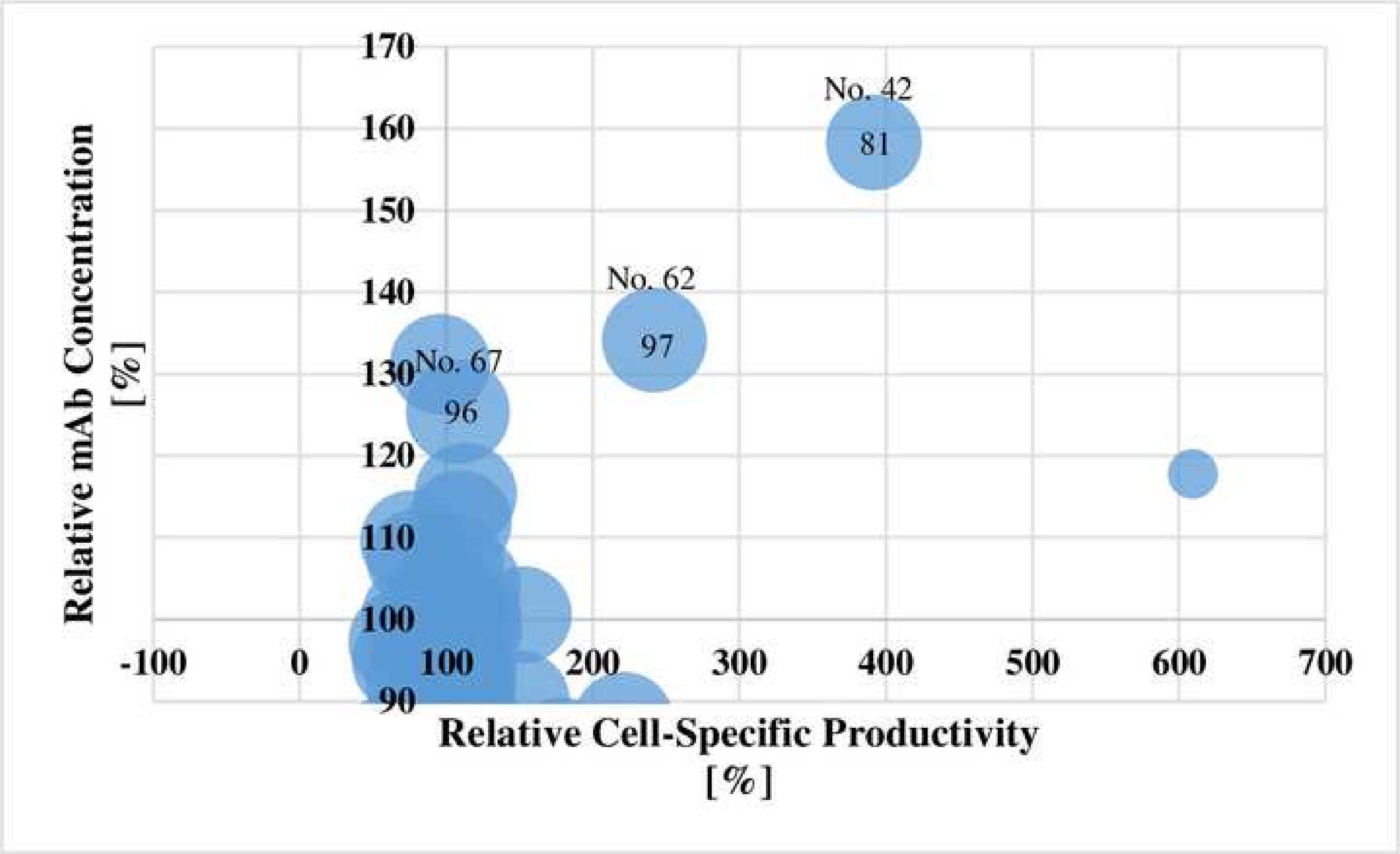

Click here to access/download;Figure;Fig 3.tif

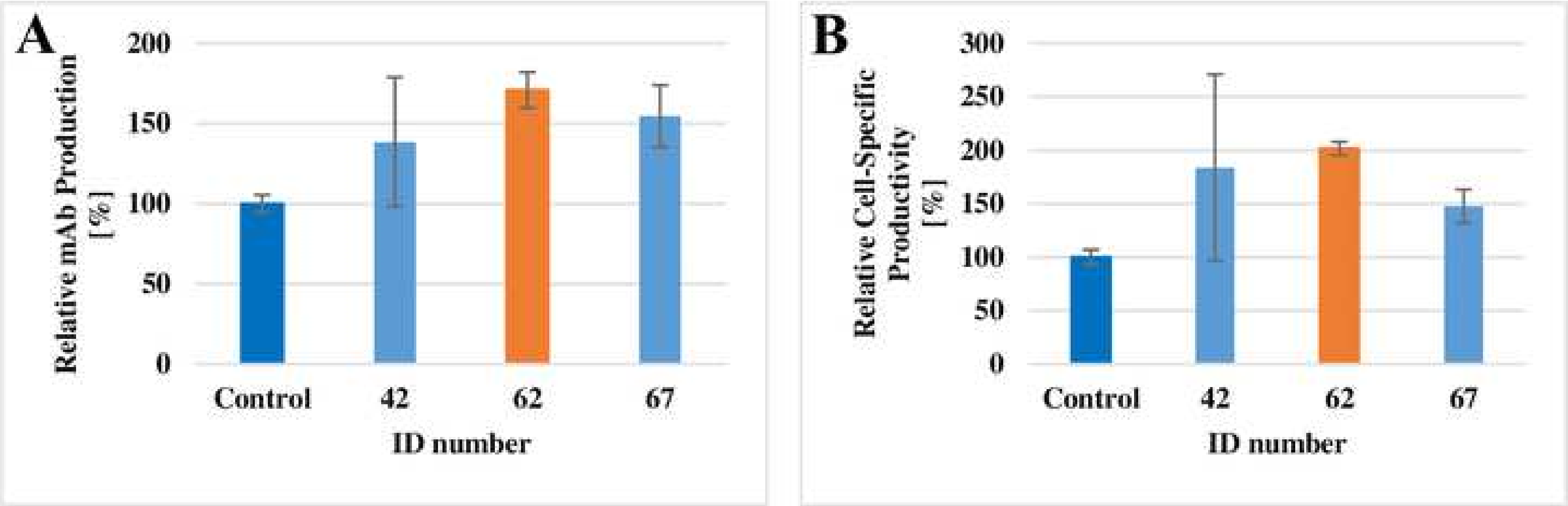

Click here to access/download;Figure;Fig 4.tif

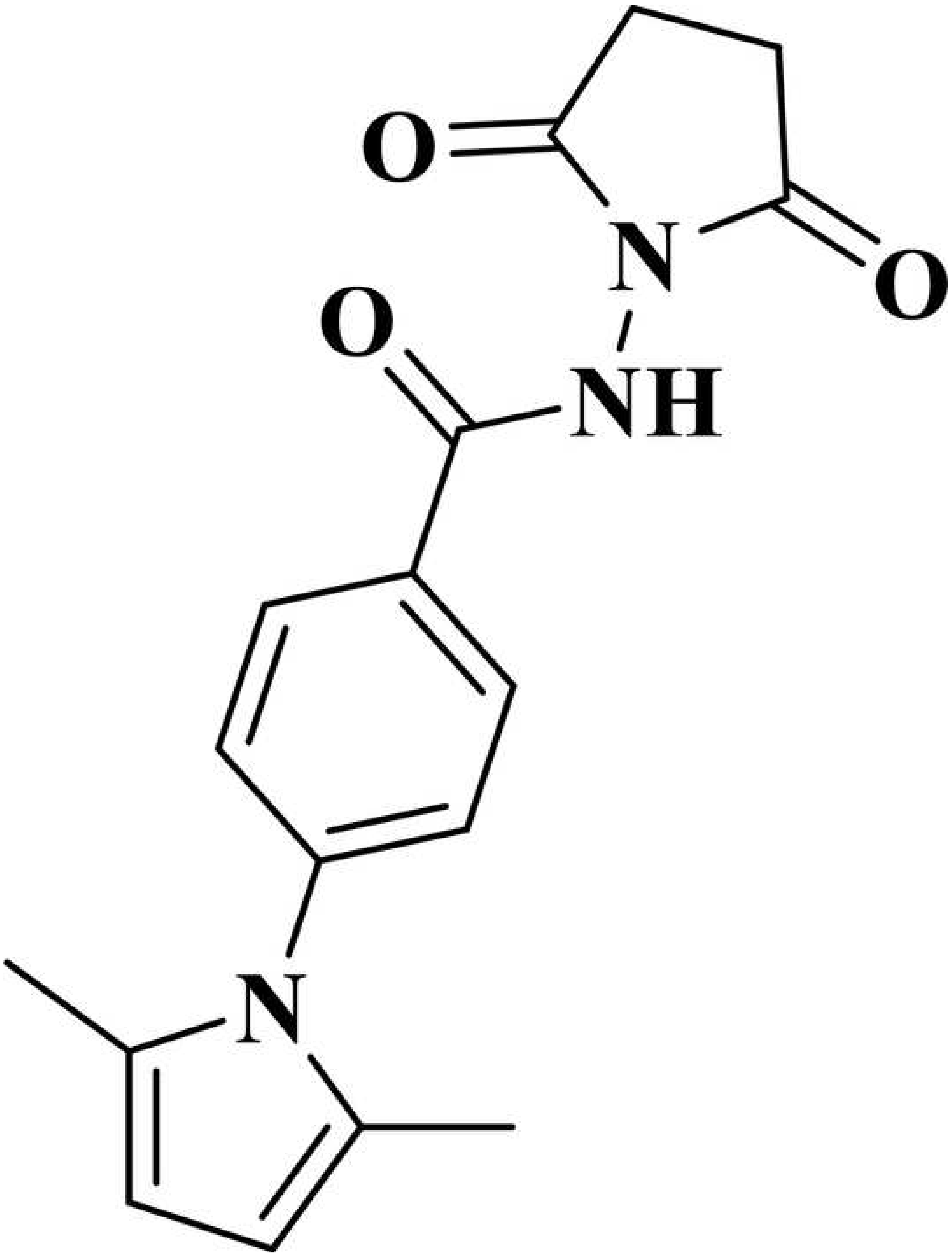

Click here to access/download;Figure;Fig 5.tif

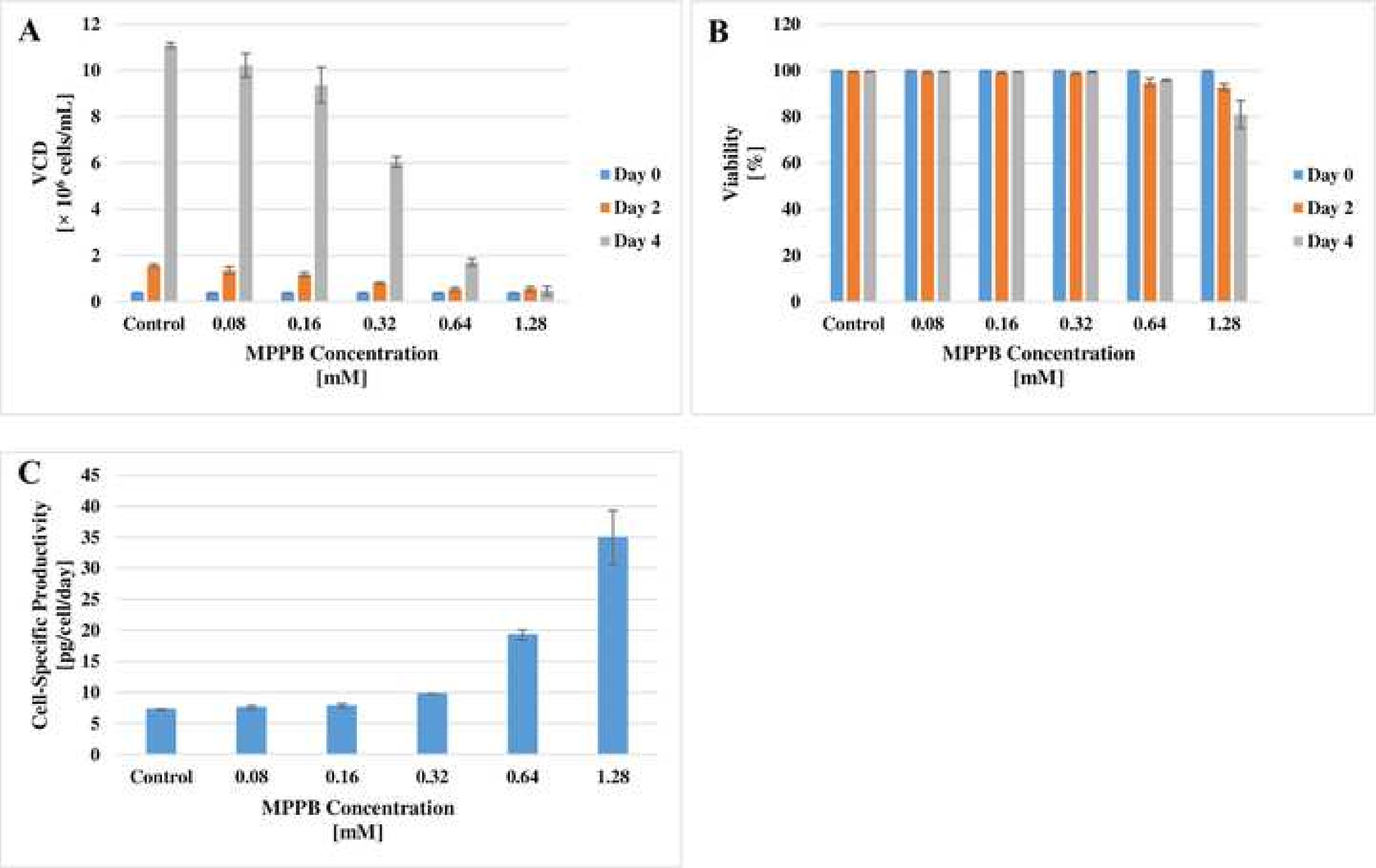

Click here to access/download;Figure;Fig 6.tif

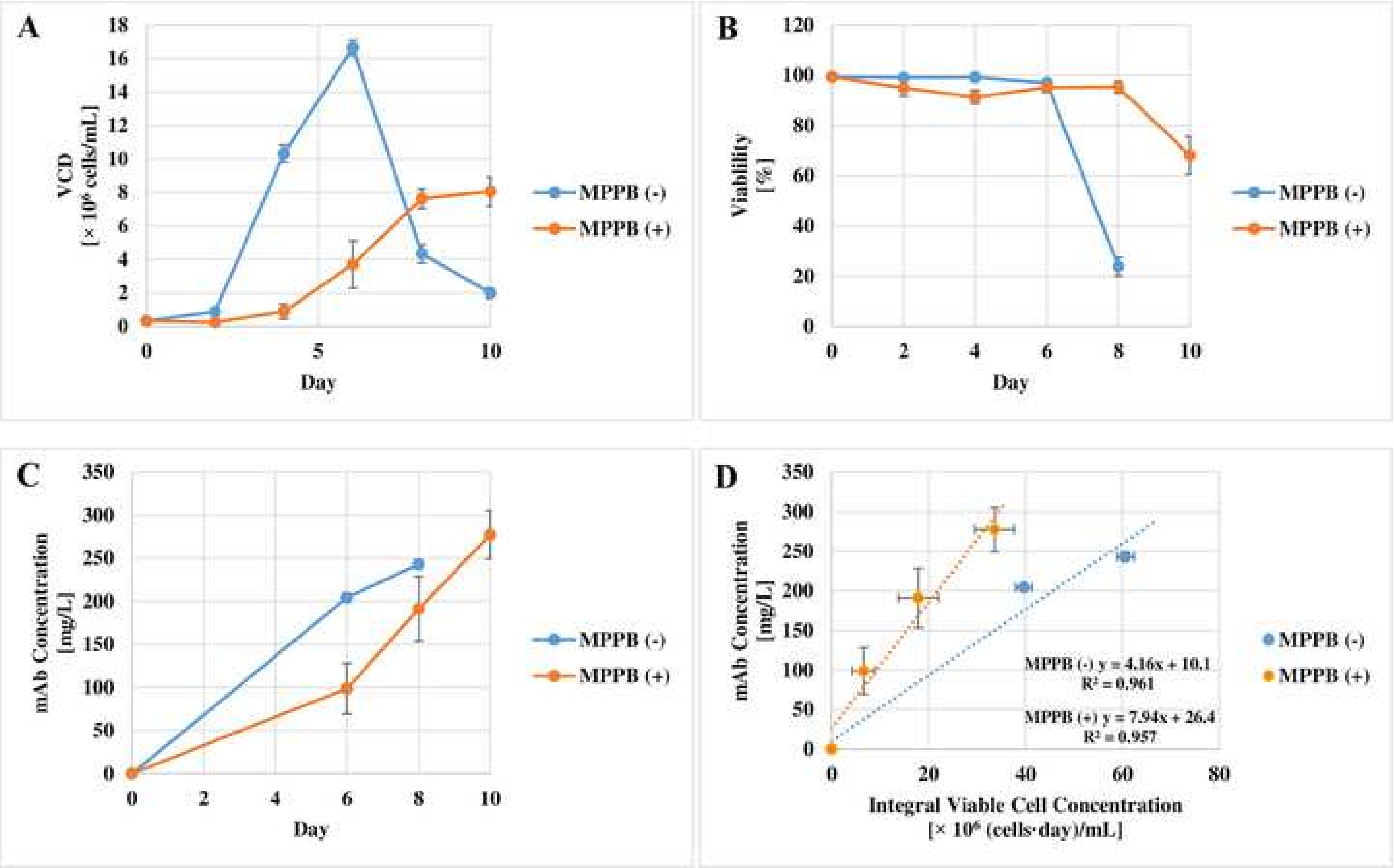

Click here to access/download;Figure;Fig 7.tif

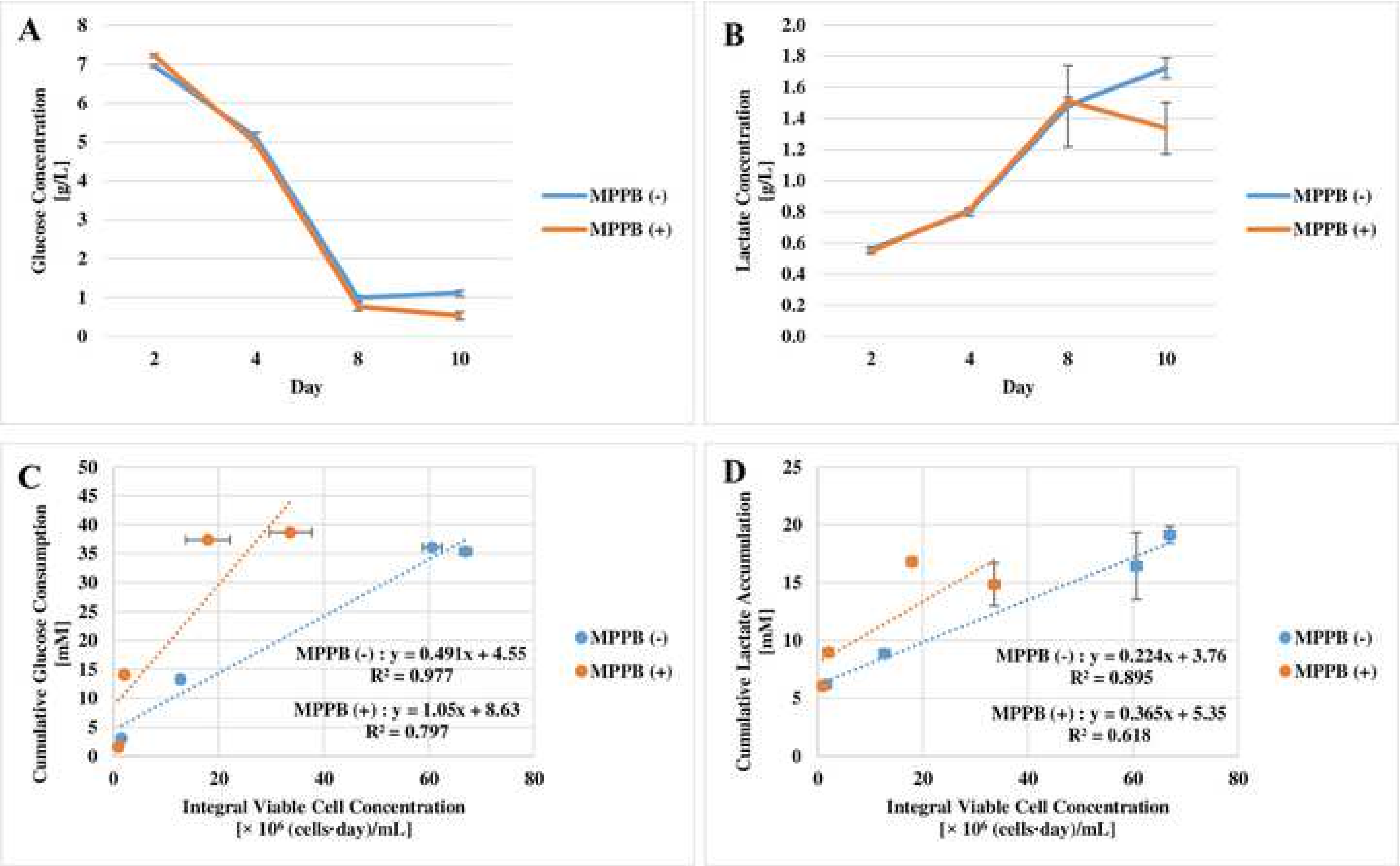

Click here to access/download;Figure;Fig 8.tif

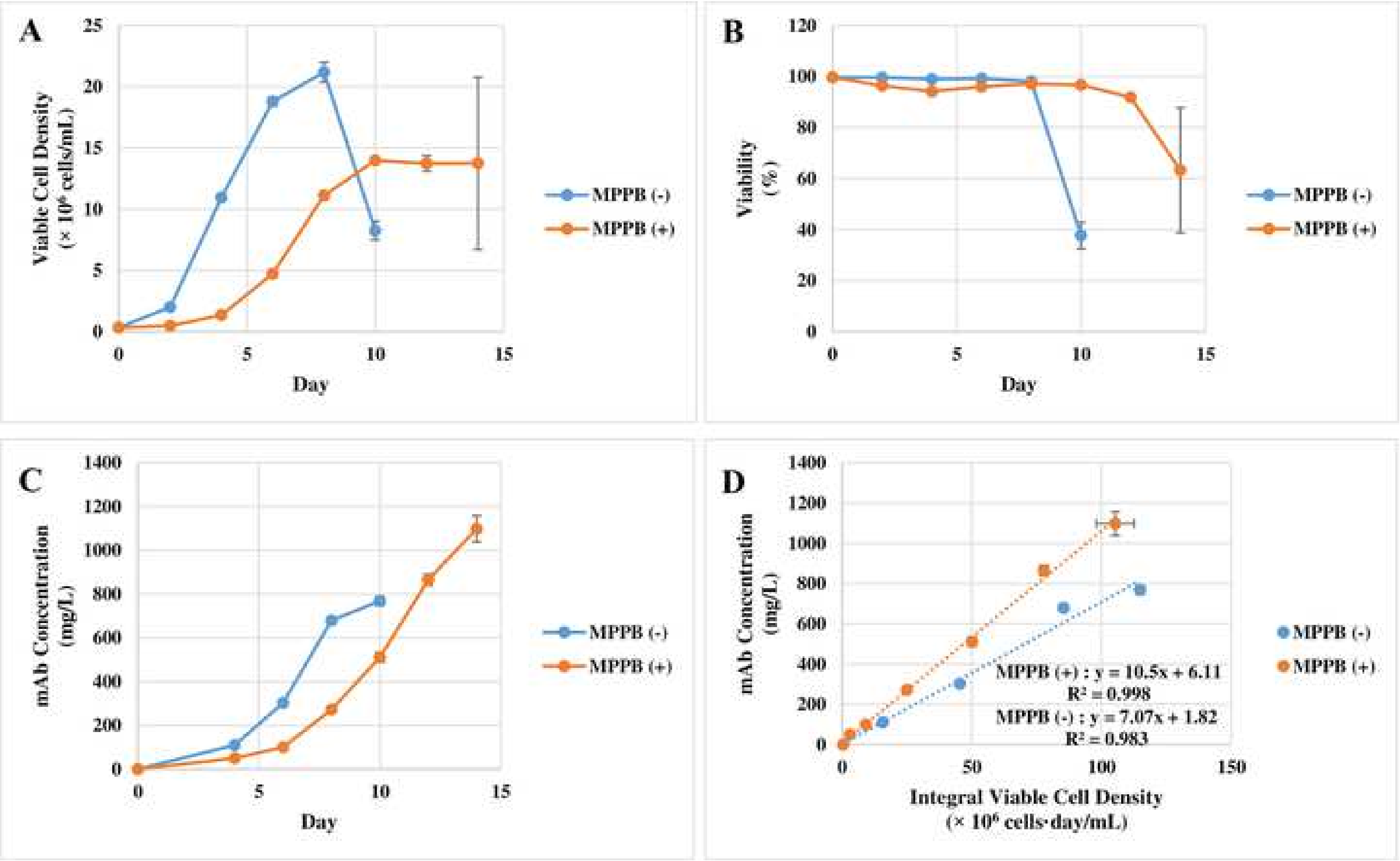

Click here to access/download;Figure;Fig 9.tif

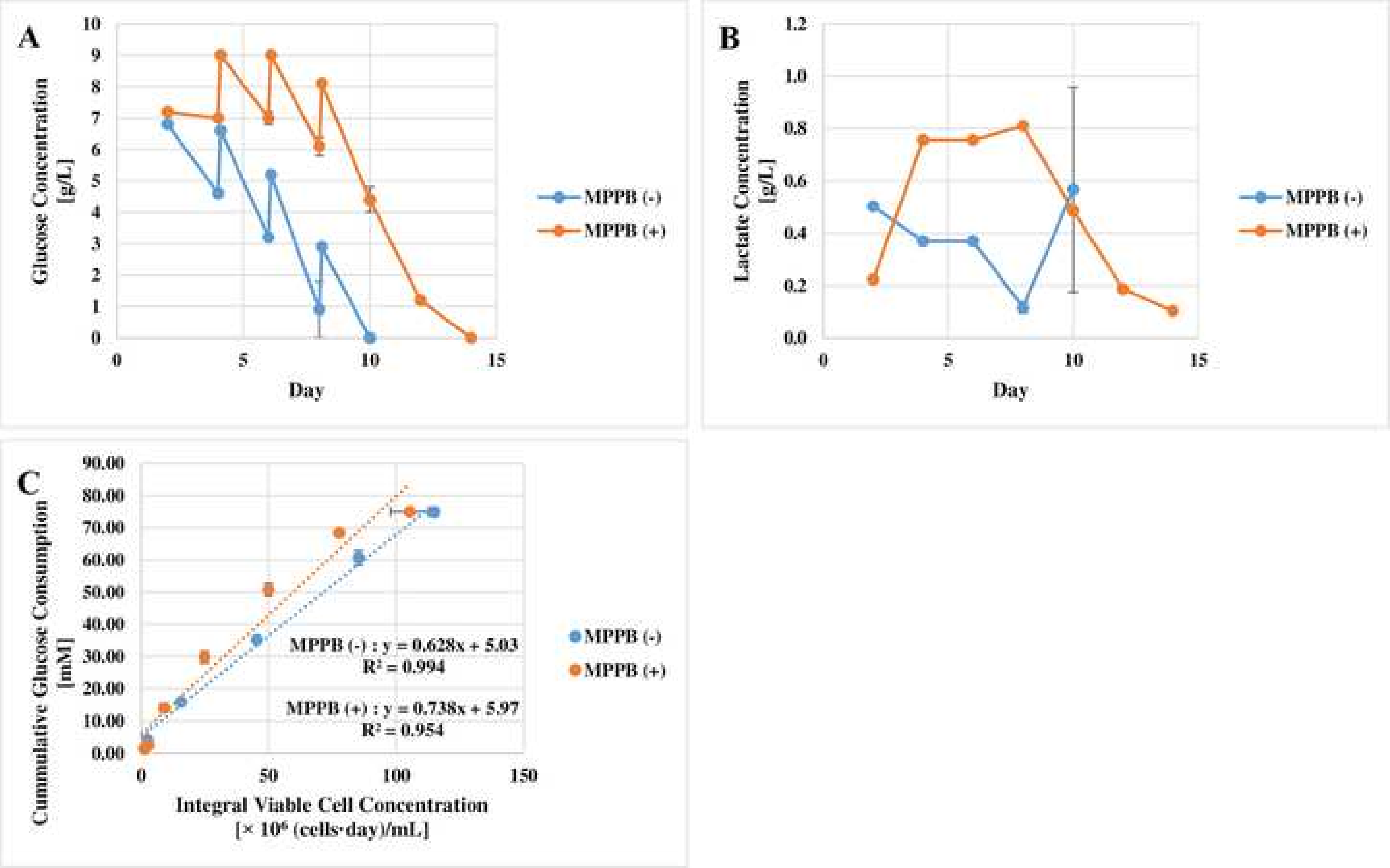

Click here to access/download;Figure;Fig 10.tif

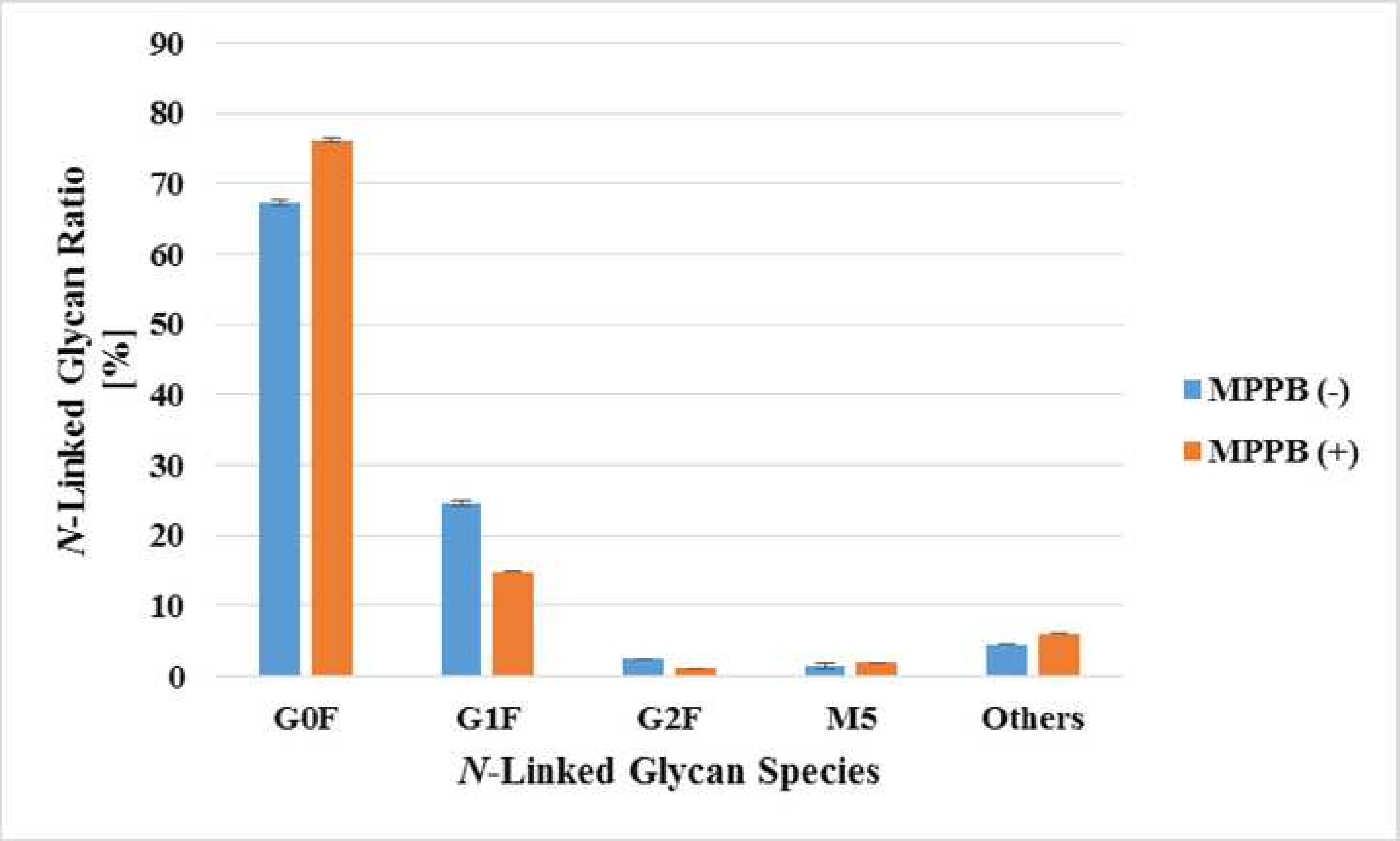

Click here to access/download;Figure;Fig 11.tif

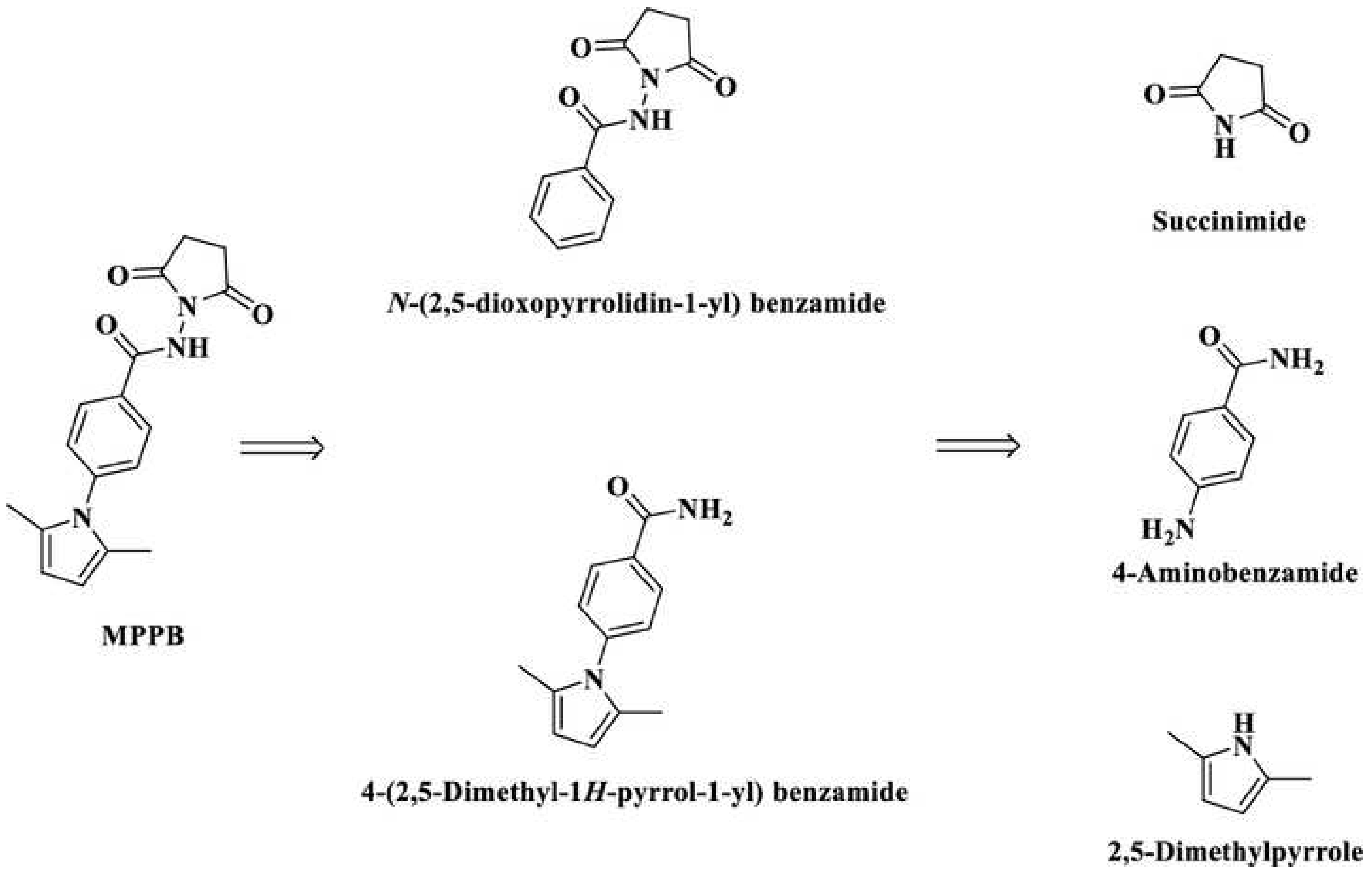

Click here to access/download;Figure;Fig 12.tif

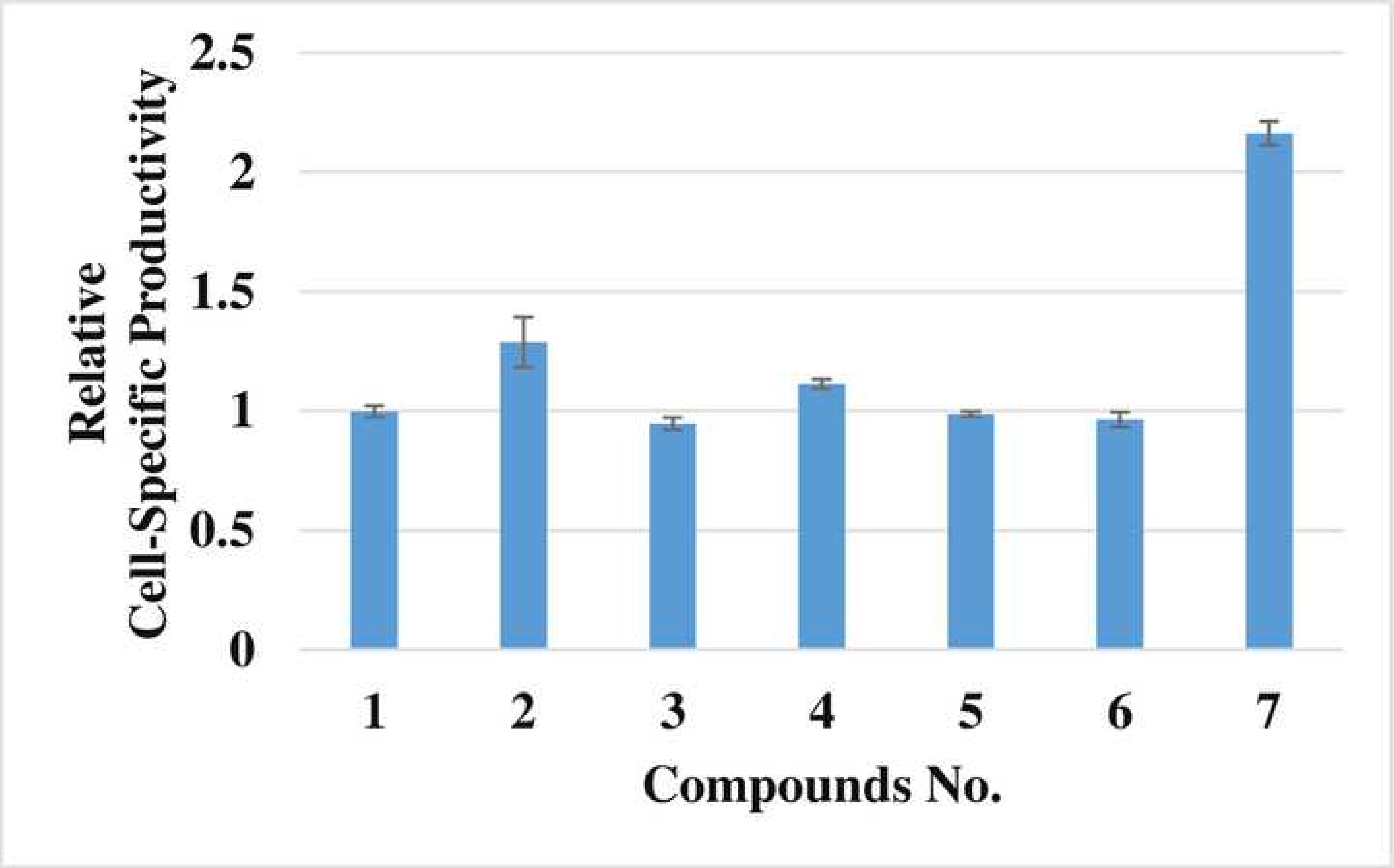

Click here to access/download
Supporting Information
S1_Fig.tif

Click here to access/download
Supporting Information
S2_Fig.tif

Click here to access/download
Supporting Information
S1 Table.tif
