# Supplementary material for: 4-(2,5-Dimethyl-1H-pyrrol-1-yl)-N-(2,5-dioxopyrrolidin-1-yl) benzamide improves monoclonal antibody production in a Chinese hamster ovary cell culture
Source: PLoS One. 2021 Apr 22;16(4):e0250416. doi: 10.1371/journal.pone.0250416 (PMC8061942; doi:10.1371/journal.pone.0250416)
Supplement: Attachment — Submitted filename: PONE-D-20-26236_R1_25Feb21.pdf [file pone.0250416.s007.pdf]

PLOS ONE

4-(2,5-Dimethyl-1H-pyrrol-1-yl)-N-(2,5-dioxopyrrolidin-1-yl) benzamide improves monoclonal antibody production in a Chinese hamster ovary cell culture

--Manuscript Draft--

**Manuscript Number:**	PONE-D-20-26236R1
**Article Type:**	Research Article
**Full Title:**	4-(2,5-Dimethyl-1H-pyrrol-1-yl)-N-(2,5-dioxopyrrolidin-1-yl) benzamide improves monoclonal antibody production in a Chinese hamster ovary cell culture
**Short Title:**	A 2,5-dimethylpyrrol derivative improves monoclonal antibody productivity in rCHO cell culture
**Corresponding Author:**	Yuichi Aki<br>Daiichisankyo Co., Ltd.<br>Chiyoda-machi, Oura-gun, Gunma JAPAN
**Keywords:**	low molecular compounds; cell culture; Chinese Hamster Ovary; galactosylation; adenosine triphosphate; monoclonal antibody production
**Abstract:**	There is a continuous demand to improve monoclonal antibody production for medication supply and medical cost reduction. For over 20 years, recombinant Chinese hamster ovary cells have been used as a host in monoclonal antibody production due to robustness, high productivity and ability to produce proteins with ideal glycans. Some chemical compounds such as dimethyl sulfoxide, lithium chloride and butyric acid, and so on, have been shown to improve monoclonal antibody production in these cell cultures. In this study, we aimed to discover new chemical compounds that can improve cell-specific antibody production in recombinant Chinese hamster ovary cells. Out of the 23,227 chemicals screened in this study, 4-(2,5-dimethyl-1 H -pyrrol-1-yl)- N -(2,5-dioxopyrrolidin-1-yl) benzamide was found to increase monoclonal antibody production. The compound has characteristics that suppress cell growth and increase cell-specific glucose uptake rate and amount of intracellular adenosine triphosphate during monoclonal antibody production. In addition, the compound also suppressed the galactosylation on a monoclonal antibody, which is a critical quality attribute of therapeutic monoclonal antibodies. Therefore, the compound might also be used to control the level of the galactosylation for the N -linked glycans. Further, the structure-activity relationship study revealed that 2,5-dimethylpyrrole was the most effective partial structure of 4-(2,5-dimethyl-1 H -pyrrol-1-yl)- N -(2,5-dioxopyrrolidin-1-yl) benzamide on monoclonal antibody production. Further structural optimization of 2,5-dimethylpyrrole derivatives could lead to improved production and quality control of monoclonal antibodies.
**Order of Authors:**	Yuichi Aki<br>Yuta Katsumata<br>Hirofumi Kakihara<br>Koichi Nonaka<br>Kenshu Fujiwara
**Response to Reviewers:**	Responses to Reviewer #1:<br>Thank you for your advice, which was valuable as we revised our manuscript. We appreciate your suggestions regarding grammar and scientific wording. The revised document “PONE-D-20-26236_reviewer V1_authors V1” was submitted in addition to this manuscript. The file will be shared by Yong-Bin Yan, Ph.D.<br><br>Responses to Reviewer #2:<br>Thank you very much for your essential questions, which we addressed to strengthen our study. The questions helped us to improve our manuscript and to focus the direction of our future research.
**Additional Information:**	
**Question**	**Response**

Powered by Editorial Manager® and ProduXion Manager® from Aries Systems Corporation

**Financial Disclosure**<br><br>Enter a financial disclosure statement that describes the sources of funding for the work included in this submission. Review the submission guidelines for detailed requirements. View published research articles from *PLOS ONE* for specific examples.<br><br>This statement is required for submission and **will appear in the published article** if the submission is accepted. Please make sure it is accurate.<br><br>**Unfunded studies**<br>Enter: *The author(s) received no specific funding for this work.*<br><br>**Funded studies**<br>Enter a statement with the following details:<br>• Initials of the authors who received each award<br>• Grant numbers awarded to each author<br>• The full name of each funder<br>• URL of each funder website<br>• Did the sponsors or funders play any role in the study design, data collection and analysis, decision to publish, or preparation of the manuscript?<br>• **NO** - Include this sentence at the end of your statement: *The funders had no role in study design, data collection and analysis, decision to publish, or preparation of the manuscript.*<br>• **YES** - Specify the role(s) played.<br><br>* typeset	1. The chemical compound library used in this study was provided by the Platform Project for Supporting Drug Discovery and Life Science Research (the Basis for Supporting Innovative Drug Discovery and Life Science Research [BINDS] program) from the Japan Agency for Medical Research and Development (AMED) under grant numbers JP19am0101086, JP19am0101084, and JP19am0101095 (support numbers 1009, 0847, and 1011, respectively). The funders had no role in the study design, data collection, data analysis, decision to publish, or preparation of the manuscript.<br>2. Daiichi Sankyo Co., Ltd., provided support in the form of salaries for the authors and supporters (YA, YK, HK, KN, AK, and MK), but the company did not have any additional role in the study design, data collection, data analysis, decision to publish, or preparation of the manuscript. The specific roles of these authors are articulated in the Author Contributions section.
**Competing Interests**<br><br>Use the instructions below to enter a competing interest statement for this submission. On behalf of all authors, disclose any competing interests that could be perceived to bias this work—acknowledging all financial support and any other relevant financial or non-financial competing interests.<br><br>This statement **will appear in the published article** if the submission is accepted. Please make sure it is	Daiichi Sankyo Co., Ltd. employs some authors [YA, YK, HK, and KN]. This commercial affiliation does not alter our adherence to all PLOS ONE policies on sharing data and materials.

accurate. View published research articles from *PLOS ONE* for specific examples.<br><br>**NO authors have competing interests**<br><br>Enter: *The authors have declared that no competing interests exist.*<br><br>**Authors with competing interests**<br><br>Enter competing interest details beginning with this statement:<br><br>*I have read the journal's policy and the authors of this manuscript have the following competing interests: [insert competing interests here]*<br><br>* typeset	
**Ethics Statement**<br><br>Enter an ethics statement for this submission. This statement is required if the study involved:<br><br>• Human participants<br>• Human specimens or tissue<br>• Vertebrate animals or cephalopods<br>• Vertebrate embryos or tissues<br>• Field research<br><br>Write "N/A" if the submission does not require an ethics statement.<br><br>General guidance is provided below. Consult the submission guidelines for detailed instructions. **Make sure that all information entered here is included in the Methods section of the manuscript.**	N/A

**Format for specific study types**<br><br>**Human Subject Research (involving human participants and/or tissue)**<br>• Give the name of the institutional review board or ethics committee that approved the study<br>• Include the approval number and/or a statement indicating approval of this research<br>• Indicate the form of consent obtained (written/oral) or the reason that consent was not obtained (e.g. the data were analyzed anonymously)<br><br>**Animal Research (involving vertebrate animals, embryos or tissues)**<br>• Provide the name of the Institutional Animal Care and Use Committee (IACUC) or other relevant ethics board that reviewed the study protocol, and indicate whether they approved this research or granted a formal waiver of ethical approval<br>• Include an approval number if one was obtained<br>• If the study involved *non-human primates*, add *additional details* about animal welfare and steps taken to ameliorate suffering<br>• If anesthesia, euthanasia, or any kind of animal sacrifice is part of the study, include briefly which substances and/or methods were applied<br><br>**Field Research**<br><br>Include the following details if this study involves the collection of plant, animal, or other materials from a natural setting:<br>• Field permit number<br>• Name of the institution or relevant body that granted permission	
**Data Availability**<br><br>Authors are required to make all data underlying the findings described fully available, without restriction, and from the time of publication. PLOS allows rare exceptions to address legal and ethical concerns. See the PLOS Data Policy and FAQ for detailed information.	No - some restrictions will apply

A Data Availability Statement describing where the data can be found is required at submission. Your answers to this question constitute the Data Availability Statement and **will be published in the article**, if accepted.<br><br>**Important:** Stating 'data available on request from the author' is not sufficient. If your data are only available upon request, select 'No' for the first question and explain your exceptional situation in the text box.<br><br>Do the authors confirm that all data underlying the findings described in their manuscript are fully available without restriction?	
**Describe where the data may be found in full sentences. If you are copying our sample text, replace any instances of XXX with the appropriate details.**<br><br>• If the data are **held or will be held in a public repository**, include URLs, accession numbers or DOIs. If this information will only be available after acceptance, indicate this by ticking the box below. For example: *All XXX files are available from the XXX database (accession number(s) XXX, XXX.*).<br>• If the data are all contained **within the manuscript and/or Supporting Information files**, enter the following: *All relevant data are within the manuscript and its Supporting Information files.*<br>• If neither of these applies but you are able to provide **details of access elsewhere**, with or without limitations, please do so. For example:<br><br>*Data cannot be shared publicly because of [XXX]. Data are available from the XXX Institutional Data Access / Ethics Committee (contact via XXX) for researchers who meet the criteria for access to confidential data.*<br><br>*The data underlying the results presented in the study are available from (include the name of the third party*	The chemical compound library used for screening in this study is owned by the Basis for Supporting Innovative Drug Discovery and Life Science Research (BINDS) program. BINDS has contractually imposed upon us restrictions involving data availability. The contact information of the data access committee and other institutional bodies are as follows:<br><br>Data access committee<br>Basis for Supporting Innovative Drug Discovery and Life Science Research (BINDS)<br>1-1-1, Yayoi, Bunkyo-ku, Tokyo 113-8657, Japan<br>E-mail: assist@mail.ecc.u-tokyo.ac.jp<br>Website: https://www.binds.jp/<br><br>Other institutional bodies<br>1.University of Tokyo<br>7-3-1, Hongo, Bunkyo-ku, Tokyo 113-0033, Japan<br>E-mail: ddiinfo@mol.f.u-tokyo.ac.jp<br><br>2.Osaka University<br>1-6, Yamadaoka, Fukida-shi, Osaka 565-0871, Japan<br>E-mail: yakugaku-syomu@office.osaka-u.ac.jp<br><br>3.Tohoku University<br>6-3, Aoba, Aramaki-aza, Aoba-ku, Sendai-shi, Miyagi 980-8578, Japan<br>E-mail: www@mail.pharm.tohoku.ac.jp<br><br>There are no restrictions on the other data we studied. These data are included in the paper and Supporting Information files.

Powered by Editorial Manager® and ProduXion Manager® from Aries Systems Corporation

and contact information or URL).<br>• This text is appropriate if the data are owned by a third party and authors do not have permission to share the data.<br><br>* typeset	
Additional data availability information:	

Powered by Editorial Manager® and ProduXion Manager® from Aries Systems Corporation

Manuscript Click here to access/download;Manuscript;Manuscript.docx

4-(2,5-Dimethyl-1*H*-pyrrol-1-yl)-*N*-(2,5-dioxopyrrolidin-1-yl) benzamide improves monoclonal antibody production in a Chinese hamster ovary cell culture

A 2,5-dimethylpyrrol derivative improves monoclonal antibody productivity in rCHO cell culture

Yuichi Aki[1,2,¶,*], Yuta Katsumata[1,&], Hirofumi Kakihara[1,&], Koichi Nonaka[1,&], Kenshu Fujiwara[2,¶]

[1] Biologics Technology Research Laboratories, Biologics Division, Daiichi Sankyo Co., Ltd., Chiyoda-machi, Gunma, Japan

[2] Department of Life Science, Akita University, Tegata Gakuen-machi, Akita, Japan

*Corresponding author

E-mail: aki.yuichi.ha@daiichisankyo.co.jp (YA)

[¶]These authors contributed equally to this work.

[&]These authors also contributed equally to this work.

Abstract

There is a continuous demand to improve monoclonal antibody production for medication supply and medical cost reduction. For over 20 years, recombinant Chinese hamster ovary cells have been used as a host in monoclonal antibody production due to robustness, high productivity and ability to produce proteins with ideal glycans. Some chemical compounds such as dimethyl sulfoxide, lithium chloride and butyric acid, and so on, have been shown to improve monoclonal antibody production in ~~these~~ cell cultures. In this study, we aimed to discover new chemical compounds that can improve cell-specific antibody production in recombinant Chinese hamster ovary cells. Out of the 23,227 chemicals screened in this study, 4-(2,5-dimethyl-1*H*-pyrrol-1-yl)-*N*-(2,5-dioxopyrrolidin-1-yl) benzamide was found to increase monoclonal antibody production. The compound has characteristics that suppress cell growth and increase cell-specific glucose uptake rate and amount of intracellular adenosine triphosphate during monoclonal antibody production. In addition, the compound also suppressed the galactosylation on a monoclonal antibody, which is a critical quality attribute of therapeutic monoclonal antibodies. Therefore, the compound might also be used to control the level of the galactosylation for the *N*-linked glycans. Further, the structure-activity relationship study revealed that 2,5-dimethylpyrrole was the most effective partial structure of 4-(2,5-dimethyl-1*H*-pyrrol-1-yl)-*N*-(2,5-dioxopyrrolidin-1-yl) benzamide on monoclonal antibody production. Further structural optimization of 2,5-dimethylpyrrole derivatives could lead to improved production and quality control of monoclonal antibodies.

Introduction

The use of therapeutic monoclonal antibodies (mAb) is becoming more common in the treatment of cancer (~~with drugs such as~~ pembrolizumab, nivolumab, trastuzumab, ~~and so on~~) and autoimmune diseases (~~with drugs such as~~ adalimumab, infliximab, and so on) [1-7]. Pharmaceutical and biologics companies have strongly focused on increasing mAb supply and reducing manufacturing costs for commercialization and sustainable growth. MAb concentrations of several grams per liter, produced by using recombinant Chinese hamster ovary (rCHO) cells, which harbor genes encoding for mAb, has been achieved in fed-batch cultures through effective host-vector system development [8, 9], genetically engineered host breeding [10], custom media development [11, 12], culture parameter optimization [13, 14]~~, and so on~~. ~~However,~~ efforts have been continued to establish cost-effective manufacturing processes by reducing the use of expensive materials such as cell culture media and column resins. It is also known that adding chemical compounds in cell cultures improves mAb production. Various chemical compounds such as dimethyl sulfoxide (DMSO) [15], lithium chloride [16], butyric acid [17], valeric acid [18], valproic acid [19], phenolic antioxidants [20], thymidine [21], and CDK4/6 inhibitor [22] have the potential to enhance mAb production when added to the cell culture at an adequate concentration to control the intracellular state of rCHO cells. In addition, 3-methyladenine up-regulates the unfolded protein response pathway and improves production [23], and compound 7312, which works as a caspase inhibitor, suppresses apoptosis and improves production [24]. To pursue the possibility of the chemical compound approach, potential synthetic compounds that might enhance mAb production in rCHO cells were gathered

from the Basis for Supporting Innovative Drug Discovery and Life Science Research (BINDS) program and evaluated by screening in batch cultures. We discovered that 4-(2,5-dimethyl-1*H*-pyrrol-1-yl)-*N*-(2,5-dioxopyrrolidin-1-yl) benzamide (MPPB), which was developed as an anti-tuberculosis therapeutic compound [25-27], stimulated mAb production in cell cultures. To reveal the characteristics of the compound, we investigated cell culture trends and the metabolism of rCHO cells under MPPB-added conditions. These studies were executed in both batch and fed-batch cultures. The results showed that MPPB improved mAb production in retaining viability, suppressed cell growth, and increased cell-specific glucose uptake rate and intracellular adenosine triphosphate amounts. MPPB not only improved mAb production, but it also affected the *N*-glycan profile. The structure-activity relationship of MPPB was also evaluated using the compounds corresponding to the partial structures of MPPB as additives in batch cultures and demonstrated that 2,5-dimethylpyrrole exhibited a strong enhancement of the cell-specific productivity of mAb.

Materials and Methods

Cell line and cell culture media

The tested rCHO cells, which expressed the mAb (Immunoglobulin G1), were generated from CHO-S host cells (Thermo Fisher Scientific, Waltham, MA, USA) by Daiichi Sankyo Co., Ltd. In-house

chemically defined media in which L-alanyl-L-glutamine concentration was adjusted to 1 mM was used as expansion and basal media. In-house chemically defined feed media was used in the fed-batch cultures.

Chemical compound library

The chemical compound library was provided by the University of Tokyo, Osaka University, and Tohoku University, which are members of Basis for Supporting Innovative Drug Discovery and Life Science Research (BINDS) which is a program that supports drug discovery.

Expansion culture condition

The rCHO cells were inoculated at a target viable cell concentration of 0.3 × 10^6 cells/mL in 125-mL or 250-mL Erlenmeyer flasks (Corning, Corning, NY, USA) with expansion medium. The working volume was adjusted to 50 mL in the 125-mL flasks and to 100 mL in the 250-mL flasks. The culture solutions were incubated for 3 to 4 days at 37.0 °C in 5% CO_2-enriched air with the shaker set to 120 rpm (20-mm stroke length).

Screening of the chemical compound library

The rCHO cells were inoculated at a target viable cell concentration of 0.3 × 10^6 cells/mL in CELLSTAR 96-well suspension culture plates (Greiner Bio-One, Frickenhausen, Germany) with expansion medium (198 µL). Rows 1 and 12 on the 96-well plates were used as controls with DMSO (2

μL). On day 0, 2 μL of each chemical compound dissolved in DMSO was added to each well. The plates were incubated at 37.0 °C in 5% CO_2-enriched air with the shaker set to 120 rpm (20-mm stroke length). After 3 days of culture, mAb concentrations were analyzed with Octet QKe (ForteBio, Fremont, CA, USA). The resulting mAb concentrations were converted to arbitrary units according to the following equation (1).

$$\text{Arbitrary unit} = (\text{tested mAb concentration} - (\text{average mAb concentration of the controls conditions} + 3\ \text{standard deviations})) \quad (1)$$

The compounds for which the arbitrary unit showed a positive value were selected as candidates for the secondary screening. The validity of this screening protocol is shown in S1 Table.

Batch culture condition

In a secondary screening to evaluate the candidates, rCHO cells were inoculated at a target viable cell concentration of 0.3 × 10^6 cells/mL in 50-mL suspension culture flasks (Greiner Bio-One) with basal medium. The working volume was adjusted to 10 mL. The culture solutions were incubated for 3 days in 5% CO_2-enriched air at 37.0 °C on static culture. All tested compounds were dissolved in DMSO at a concentration of 100 mg/mL, and 10 μL (0.1% v/v) of this solution was added to a culture on day 0. In the suspension batch cultures, the rCHO cell seeding flasks were incubated for 3 to 10 days in 5% CO_2-enriched air at 37.0 °C with the shaker set to 120 rpm (20-mm stroke length).

Fed-batch culture condition

The rCHO cells were inoculated at a target viable cell concentration of 0.3 × 10^6 cells/mL in 125-mL Erlenmeyer flasks (Corning) with basal medium. The working volume was adjusted to 50 mL. The culture solutions were incubated at 37.0 °C in 5% CO_2-enriched air with the shaker set to 120 rpm (20-mm stroke length) until less than 70% viability was achieved. On days 4, 6, and 8, feed medium at 2% v/v against the starting volume was added. On day 0, 50 µL (0.1% v/v) of 4-(2,5-dimethyl-1*H*-pyrrol-1-yl)-*N*-(2, 5-dioxopyrrolidin-1-yl) benzamide (Abamachem Ltd., Kyiv, Ukraine) dissolved in DMSO at a concentration of 200 mg/mL was added.

Measurement of viable cell density and viability

Vi-CELL XR (Beckman Coulter, CA, USA) was used to evaluate viable cell density (VCD) and viability according to the manufacturer's instructions. All testing samples were diluted twice except for on days 0 to 4.

Metabolite analysis

Glucose and lactate concentrations in the cultures were analyzed by BioProfile FLEX2 (Nova Biomedical, Waltham, MA, USA) according to the manufacturer's protocol.

Concentration of mAb

Cell culture solution was filtrated with a 0.2-µm filter. The mAb concentration in the residual solution was measured by high-performance liquid chromatography (HPLC) with a Protein A affinity chromatography column, which is a PA ImmunoDetection Sensor Cartridge, with an ID of 2.1 × 30 mm (Applied Biosystems, Bedford, MA, USA).

Measurement of intracellular ATP

An intracellular ATP assay kit (v2, Toyo B-Net, Tokyo, Japan) was used to quantitate ATP amounts in rCHO cells. Each cell culture solution (100 µL) was diluted with phosphate-buffered saline (1 mL) and then centrifuged (450 g, 5 min), and the supernatant was discarded (1 mL). The resulting cell pellet was suspended in phosphate-buffered saline (1 mL) and then centrifuged (450 g, 5 min), and the supernatant was again discarded (1 mL). The cell pellet was suspended in the ATP extraction reagent from the kit (0.5 mL) and incubated for 5 minutes to extract ATP from the cells. The suspension (10 µL) was mixed with luciferase luminescence assay reagents (100 µL), and measurements were obtained by using the Infinite M Plex plate reader (Tecan, Mannedorf, Switzerland). The resulting ATP concentration was converted to the amount of intracellular ATP per cell

Calculation of cell-specific productivity, cell-specific glucose uptake rates, and cell-specific lactate production rates

Cell-specific productivity (pg/cell/day), cell-specific glucose uptake rates (pmol/cell/day), and cell-specific lactate production rates (pmol/cell/day) were calculated as the slopes of mAb concentration (mg/L), consumed glucose concentration (mM), and lactate concentration (mM), respectively, to integral viable cell concentration ((cells·day)/mL), according to previous reports [28, 29].

N-linked glycan analysis

The samples purified with the spin column-based antibody purification kit (Cosmo Bio, Tokyo, Japan) according to the manufacturer's protocol were labeled with 2-AB using the EZGlyco mAb-N kit (Sumitomo Bakelite, Tokyo, Japan) for *N*-linked glycan analysis, which was performed under gradient conditions of 50 mM ammonium formate buffer (pH 4.4) on HPLC using a XBridge BEH Amide XP Column (2.5 µm, ID 4.6 × 150 mm, Waters Corporation, Milford, MA, USA) at 60 °C. The detected wavelength was 420 nm, and the flow rate was changed from 0.4 to 1.0 mL/min under gradient conditions.

Statistical analysis

At least 3 batches were executed for statistical analysis. The mean ± standard deviation (SD) and P-value were calculated using JMP statistical analysis software (SAS, NC, USA). Differences in the data were considered significant at $P < 0.05$.

Results

Initial screening of the chemical compound library

A total of 23,227 chemical compounds were evaluated by cultivation in 96-well plates as an initial screening process (Fig 1). In the rCHO cell cultures, 566 compounds showed a positive effect in terms of increased mAb production. The hit rate was 2.5%. In parallel, we investigated the commercial availability of the first screening positives before starting the second screening to obtain sufficient amounts of the positives for further study. Although 67 chemical compounds were commercially available, another 5 compounds for which the original structures were not commercially available were also tested as derivatives. Therefore, a total of 72 samples underwent the second screening in suspension culture.

Fig 1. Results of the first screening to evaluate the effect of the chemical compounds on mAb production. Higher mAb production is shown as a positive arbitrary unit.

Second screening of the selected compounds in a suspension culture

Seventy-two chemical compounds were further evaluated in a suspension culture for 3 days. The relative cell-specific productivity, relative mAb concentration, and viability were assessed to evaluate the chemical compound treatments against the control condition. The selection criteria of the candidate compounds were set to over 120% for relative mAb concentration, 105% for relative cell-specific

productivity, and 80% for viability. The samples with the ID numbers 42, 62, and 67 met these criteria, with increases of 125 to 158% for mAb concentration and 108 to 392% for cell-specific productivity in comparison with the control condition (Fig 2).

Fig 2. Relative mAb concentration and cell-specific productivity increased with the addition of the chemical compounds. The numbers in and above the circles indicate viability and the sample's ID number, respectively.

MPPB as an effective additive of mAb production

The three selected compounds had higher relative mAb concentration and relative cell-specific productivity. ID number 62 showed an especially strong effect, with a 171% increase in relative mAb concentration and a 202% increase in relative cell-specific productivity compared with the control condition (Fig 3). As ID number 62 demonstrated the largest effect, we further investigated the characteristics of ID number 62 in batch and fed-batch cultures. The selected compound was MPPB (Fig 4), a commercially available substitute for a first-screening-hit-compound supplied from the chemical compound library of the University of Tokyo. MPPB was originally developed as an anti-tuberculosis therapeutic compound [25-27].

Fig 3. The effect of the three candidate compounds on relative mAb concentration and relative cell-specific productivity. All cell cultures were executed three times and evaluated on day 3. Each value of relative mAb production (**A**) and relative cell-specific productivity (**B**) were compared statistically.

Fig 4. The structure of 4-(2,5-dimethyl-1*H*-pyrrol-1-yl)-*N*-(2,5-dioxopyrrolidin-1-yl) benzamide (MPPB).

Effect of MPPB on the culture profile in the batch cultures

The effect of MPPB on the culture profile was tested in the batch cultures, as shown in Fig 5. MPPB suppressed cell growth depending on the concentration of MPPB added (Fig 5A). Viability was slightly decreased at a MPPB concentration of 1.28 mM (Fig 5B) and cell-specific productivity was increased by an addition of MPPB with a concentration over 0.32 mM (Fig 5C). MAb concentration decreased with the addition of MPPB due to suppressed cell growth in these short-period batch cultures (Fig 5D). These results showed that treatment with MPPB concentrations of 0.32 to 0.64 mM suppressed cell growth and increased cell-specific productivity while retaining viability. Therefore, MPPB concentrations of 0.32 to 0.64 mM were used to characterize MPPB.

Fig 5. MPPB suppresses cell growth and increases cell-specific productivity. The tested MPPB concentration is indicated in the figure. In these tests, viable cell density (**A**), viability (**B**), and cell-specific productivity (**C**), and mAb concentration (**D**) were evaluated on day 4.

Becker et al. [30] and Hara and Kondo [31] reported that increased amounts of intracellular ATP are associated with increased cell-specific productivity. Therefore, we investigated intracellular ATP amounts and cell-specific glucose uptake rates, which are related to ATP production [32, 33], in the batch cultures to clarify the reason for the increasing cell-specific productivity (Fig 6). The results showed that both intracellular ATP amounts and cell-specific glucose uptake rates were increased with higher MPPB concentrations. Intracellular ATP amounts increased from 8 to 24 fmol/cell (Fig 6A), and cell-specific glucose uptake rates increased from 0.9 to 2.6 pmol/cell/day (Fig 6B). Intracellular ATP amounts and cell-specific glucose uptake rates were positively correlated with cell-specific productivity (Fig 6C). Additionally, VCD was suppressed with MPPB treatment (Fig 6D)

Fig 6. MPPB increased both intracellular ATP amounts and cell-specific glucose uptake rates and suppressed VCD. Given previous results, the tested MPPB concentrations ranged from 0.32 to 0.64 mM. Intracellular ATP amounts (A), cell-specific glucose uptake rates (B), relative cell-specific productivity (C), and viable cell density (D) were evaluated on day 3.

MPPB was further evaluated in the batch cultures. The maximum VCD was reduced from 16.6 × 10^6 cells/mL to 8.0 × 10^6 cells/mL with MPPB treatment (Fig 7A). On the other hand, there was longer viability under the MPPB-added condition (Fig 7B). The mAb concentration under the MPPB-added

condition was almost the same value as the control condition at each harvest time point (Fig 7C). The MPPB addition increased the cell-specific productivity from 4.2 pg/cell/day to 7.9 pg/cell/day (Fig 7D).

Fig 7. Cell culture profile and mAb production in rCHO cells treated with MPPB. MPPB at 0.64 mM was added in the batch cultures on day 0. Viable cell density (**A**) and viability (**B**) were measured every 2 days. Cell culture was continued until viability below 70% was reached. The slope of mAb concentration (**C**) to integral viable cell concentration was used to indicate cell-specific productivity (**D**) [28].

From a spent media analysis, glucose and lactate concentrations showed no differences between the conditions with and without the addition of MPPB (Figs 8A and 8B). However, cell-specific glucose uptake rate increased from 0.49 to 1.1 pmol/cell/day with MPPB treatment (Fig 8C). ~~In other words, the cell-specific glucose uptake rate under the MPPB-added condition was 0.61 pmol/cell/day higher than that under the control condition.~~ Meanwhile, the cell-specific lactate production rate increased from 0.22 to 0.37 pmol/cell/day under the MPPB-added condition (Fig 8D). The increase in the cell-specific lactate production rate was 0.15 pmol/cell/day, which was smaller than the increase in the cell-specific glucose uptake rate (0.61 pmol/cell/day). This result suggested that consumed glucose was efficiently converted to ATP in the tricarboxylic acid cycle.

Fig 8. The effect of MPPB on the metabolite profile in the batch cultures. The spent media analysis of glucose concentration (**A**) and lactate concentration (**B**) was conducted every 2 days starting on day 2. Cell-specific glucose uptake rates (**C**) and cell-specific lactate production rates (**D**) were indicated as the slopes of consumed glucose concentration and lactate concentration, respectively to integral viable cell concentration [28, 29].

Effect of MPPB on the culture profile in the fed-batch cultures

Further evaluation of the MPPB-added condition was continued in the fed-batch cultures. The maximum VCD under the MPPB-added condition was reduced from 21.2 × 10^6 cells/mL in the control condition to 14.0 × 10^6 cells/mL (Fig 9A). The VCD and viability also decreased after day 8 in the control condition (Figs 9A and 9B). However, they were kept until day 12 under the MPPB-added condition. Despite ~~suppression of the~~ VCD, the final mAb concentration under the MPPB-added condition reached 1,098 mg/L which was 1.5 times higher than that under the control condition (Fig 9C). The cell-specific productivity under the MPPB-added condition also b~~ecame~~ 1.5-fold higher than that under the control condition (Fig 9D). The cell-specific productivity under the control and MPPB-added conditions was 7.1 and 11 pg/cell/day, respectively.

Fig 9. Cell culture profile and mAb production in the fed-batch cultures under the MPPB-added condition. MPPB at 0.64 mM was added to a culture on day 0, and 2% (v/v) feed medium was added on

days 2, 4, and 6. Viable cell density (**A**) and viability (**B**) were measured every 2 days. The cell culture was continued until viability below 70% was reached. The slope of the mAb concentration (**C**) and integral viable cell concentration were used as cell-specific productivity (**D**) [28].

Residual glucose was fully consumed by day 10 under the control condition, while glucose under the MPPB-added condition was retained until day 14 (Fig 10A). The cell-specific glucose uptake rates were 0.63 and 0.74 pmol/cell/day under the control and MPPB-added conditions, respectively (Fig 10C). The lactate concentration was kept under 0.8 g/L, and no difference was noted in both conditions (Fig 10B).

Fig 10. Metabolite analysis of the fed-batch cultures under the MPPB-added condition. Glucose concentration (**A**) and lactate concentration (**B**) were measured before adding the feed medium. Glucose concentration after adding the feed medium was calculated using the added feed medium amount. Cell-specific glucose uptake rates (**C**) was indicated as the slope of consumed glucose concentration against integral viable cell concentration [28, 29].

In addition, major *N*-linked glycans (G0F, G1F, G2F, and M5) on mAb were investigated on day 14 under the MPPB-added condition. G0F was the major *N*-linked glycan, and G1F was decreased from

24.5 to 14.8% under the MPPB-added condition, although G2F and M5 ratios were not changed, as shown in Fig 11.

Fig 11. MPPB suppresses galactosylation of mAb. Each value was calculated as the percentage of total *N*-linked glycans. The ratio was statically analyzed.

Structure-activity relationship of MPPB

The MPPB structure is divided into five chemical components, as follows: *N*-(2,5-dioxopyrrolidin-1-yl) benzamide; 4-(2,5-dimethyl-1*H*-pyrrol-1-yl) benzamide; succinimide; 4-aminobenzamide; and 2,5-dimethylpyrrole (Fig 12).

Fig 12. Five chemical components derived from MPPB.

The activity of the five chemical components derived from MPPB was identified and compared with that of MPPB in terms of cell-specific productivity (Fig 13). 4-(2,5-Dimethyl-1*H*-pyrrol-1-yl) benzamide and 2,5-dimethylpyrrole increased cell-specific productivity. Further, the 2,5-dimethylpyrrole-added condition had a 2.2-fold higher cell-specific productivity than the control condition.

Fig 13. Evaluation of the effect of the five chemical components derived from MPPB on cell-specific productivity. Each chemical compound was added on day 0 and evaluated on day 3. The tested concentration of each chemical compound was 0.32 mM.

Lane 1: dimethyl sulfoxide (control); lane 2: MPPB; lane 3: *N*-(2,5-dioxopyrrolidin-1-yl) benzamide; lane 4: 4-(2,5-dimethyl-1*H*-pyrrol-1-yl) benzamide; lane 5: succinimide; lane 6: 4-aminobenzamide; lane 7: 2,5-dimethylpyrrole.

Further evaluation was conducted to identify the effect of pyrrole derivatives on viability and cell-specific productivity in the batch cultures (Table 1). Cell-specific productivity with alkyl pyrrole derivatives (**2**, **9-14**) was increased up to 1.4- to 7.8-fold higher than that with the control condition, although pyrrole (**7**) and 1-alkyl pyrroles (**1**, **8**) did not show any activity. On the other hand, viability decreased below 50% with these alkyl pyrrole derivatives (**2**, **9-14**), except for 2,5-dimethylpyrrole (**13**). 2,5-Dimethylpyrrolidine (**6**), which has a reduced framework of 2,5-dimethylpyrrole (**13**), also did not affect the cell culture. Thus, 2,5-dimethylpyrrole (**13**) was found to be the most effective chemical structure to increase cell-specific productivity while retaining viability in the 0.32 mM concentration. In addition, alkyl-pyrroles (**9-14**) that showed high cell-specific productivity in the 0.32 mM concentration were tested to clarify which pyrrole derivative was the best compound in terms of increasing cell-specific productivity and retaining viability. The results showed that 2,5-dimethylpyrrole (**13**) effectively increased cell-specific productivity while retaining viability (S2 Fig).

337

Table 1. Effect of pyrrole derivatives on viability and relative cell-specific productivity in rCHO cell cultures.

Compound No.	Compound name	Viability (%)	Relative cell-specific productivity
-	Dimethyl sulfoxide (control)	99.4 (0.4)	1.0 (0.0)
-	MPPB	99.2 (0.5)	1.4 (0.2)
**1**	1-(2-Hydroxyethyl)pyrrole	99.6 (0.2)	1.0 (0.1)
**2**	2-Ethylpyrrole	41.2 (2.5)	1.4 (0.1)
**3**	Pyrrole-2-carboxaldehyde	99.3 (0.2)	1.0 (0.1)
**4**	Pyrrole-2-carboxylic acid	99.5 (0.2)	1.0 (0.1)
**5**	Pyrrolidine	98.9 (1.4)	1.0 (0.0)
**6**	2,5-Dimethylpyrrolidine	99.0 (1.0)	1.0 (0.0)
**7**	Pyrrole	99.3 (0.7)	1.0 (0.1)
**8**	1-Methylpyrrole	99.4 (0.1)	1.0 (0.1)
**9**	2-Methylpyrrole	50.2 (0.2)	3.0 (0.2)
**10**	3-Methylpyrrole	34.4 (2.0)	4.0 (0.2)
**11**	2,3-Dimethylpyrrole	2.6 (1.7)	7.8 (0.4)
**12**	2,4-Dimethylpyrrole	30.7 (3.4)	4.8 (0.3)
**13**	2,5-Dimethylpyrrole	97.9 (0.6)	2.2 (0.0)
**14**	3,4-Dimethylpyrrole	4.0 (1.2)	7.4 (0.3)

Each pyrrole derivative was added on day 0 and evaluated on day 3. The tested concentration of each pyrrole derivative was 0.32 mM. Standard deviation was indicated in the blanket.

Discussion

In this study, we screened chemical compounds to find novel additives that improve mAb production in rCHO cells and thereby promote pharmaceutical supply (Figs 1-3). MPPB had 1.5-fold

higher mAb production than the control conditions in the fed-batch cultures. This is the first study in which MPPB was applied in rCHO cell cultures to improve mAb production.

Our study revealed two key factors for the improvement of mAb production by MPPB. One was the retaining viability (Figs 7B and 9B), and the other was the increase of cell-specific productivity (Figs 7D and 9D). It is likely that these two factors explain the increased mAb production in the MPPB-added condition despite lower cell concentrations compared to the control condition.

The mechanism of MPPB in terms of retaining viability is unclear. MPPB might keep glucose concentrations high, leading to retained viability (Figs 9B and 10A). However, although the remaining glucose concentration was almost similar with and without the addition of MPPB in the batch cultures, the viability remained high under the MPPB-added condition (Figs 7B and 8A). Once, glucose was maintained above 1 g/L in the fed-batch cultures (S1 Fig), the viability under the control condition was 45% lower than that under the MPPB-added condition on day 12. These results suggested that cell growth suppression by MPPB treatment prevented the depletion of medium components except for glucose, resulting in maintained viability.

The cause of increased cell-specific productivity, however, is clearer. In our study, MPPB treatment had two important effects to increase cell-specific productivity. One was the suppression of cell growth (Figs 5A, 6D, 7A and 9A), and the other was the acceleration of cell-specific glucose uptake rates (Figs 6B, 8C and 10C), while keeping cell-specific lactate production rates low (Fig 8D). Both the effects would control the amounts of intracellular ATP. As reported by Mulukutla et al., growing cells require

more ATPs than quiescent cells [34]. An increased cell-specific glucose uptake rate leads to an increased amounts of intracellular ATP when glucose is metabolized to ATP in the tricarboxylic acid cycle [32, 33], but not when glucose is metabolized to lactate. Becker et al. [30] and Hara and Kondo [31] have reported that the energy available in the form of intracellular ATP is crucial for mAb production. Accordingly, it may be hypothesized that rCHO cells increased intracellular ATP amounts by suppressing cell growth and increasing cell-specific glucose uptake rates, thereby increasing cell-specific productivity, as expected. To test this hypothesis, we investigated the relationship between the amount of intracellular ATP and MPPB concentration (Fig 6). We found that the amount of intracellular ATP was enhanced with higher MPPB concentrations (Fig 6A). Cell-specific glucose uptake rates and cell-specific productivity were also increased (Figs 6B and 6C). On the other hand, VCD was also suppressed with higher MPPB conditions (Fig 6D). Therefore, we predicted that the increased cell-specific productivity under the MPPB-added condition might occur due to the increasing amount of intracellular ATP resulting from cell growth suppression and enhanced cell-specific glucose uptake rates. Our results showed that a chemical compound such as MPPB, which suppresses cell growth and increases intracellular ATP amounts, may improve mAb production in rCHO cell cultures.

Furthermore, we found that MPPB treatment suppressed galactosylation in *N*-linked glycans on the expressed mAb (Fig 11). The galactosylation ratio in *N*-linked glycans on mAb is one of the critical quality attributes of mAb in terms of pharmaceutical development, because the galactosylation profile affects complement-dependent cytotoxicity [35, 36] and antibody-dependent cellular cytotoxicity [37-39].

Therefore, to adjust mAb activity and maintain consistent quality, galactosylation is often controlled during pharmaceutical process research and development. Some media additives are known to affect the galactosylation of mAb in rCHO cells. For example, valeric acid, valproic acid, CDK4/6 inhibitor, galactose, manganese, and uridine encourage galactosylation [18, 19, 22, 40, 41], while zinc and ammonia suppress galactosylation [42, 43]. However, ammonia is harmful to rCHO cells [44]. The number of effective galactsylation suppressants is small, and, therefore, the suppression of galactosylation by media additives is not well understood. Our findings that MPPB suppressed the galactosylation may offer a new option to control the quality of mAb by the suppression of galactosylation in the *N*-linked glycans of mAb.

Finally, the structure-activity relationship study revealed that the most critical chemical portion of MPPB to maintain viability and improve cell-specific productivity of mAb in rCHO cells is 2,5-dimethylpyrrole (Fig 13, Table 1). Since 2,5-dimethylpyrrolidine having a reduced framework of 2,5-dimethylpyrrole showed no effect, the importance of the heteroaromatic framework of 2,5-dimethylpyrrole for both the activities was suggested. The 2,5-dialkyl substitution of pyrrole was also indispensable. The viability was decreased in the cases of other mono- or bis-alkyl-substituted pyrroles. Furthermore, non-alkylated or *N*-substituted pyrroles showed no effect on both the viability and the cell-specific productivity. Thus, the 2,5-dialkyl-substituted pyrrole framework was found to be effective on maintaining viability and improving cell-specific productivity of mAb in rCHO cells during the structure-activity relationship study. The mechanism of the action of MPPB and 2,5-dimethylpyrrole in rCHO cell cultures is currently being considered for future application studies.

Acknowledgments

The authors acknowledge the support provided by Akihiro Kawara (Daiichi Sankyo Co., Ltd.) in terms of contracting with the BINDS program, and by Masami Katase (Daiichi Sankyo Co., Ltd.) in terms of collecting commercially available chemical compounds.

References

1. Pento JT. Monoclonal antibodies for the treatment of cancer. Anticancer Res. 2017 Nov;37(11):5935-9. doi: 10.21873/anticanres.12040.
2. Yoo SM, Chung SH. Targets of monoclonal antibodies for immunological diseases. Arch. Pharm. Res. 2019;42:293-304. doi: 10.1007/s12272-018-1087-2.
3. Qu J, Wang L, Jiang M, Zhao D, Wang Y, Zhang F, et al. A review about pembrolizumab in first-line treatment of advanced NSCLC: focus on KEYNOTE studies. Cancer Manag Res. 2020;12:6493-509. doi: 10.2147/CMAR.S257188.
4. Keating GM. Nivolumab: a review in advanced nonsquamous non-small cell lung cancer. Drugs. 2016 Jun;76(9):969-78. doi: 10.1007/s40265-016-0589-9.

5. McKeage K, Perry CM. Trastuzumab: a review of its use in the treatment of metastatic breast cancer overexpressing HER2. Drugs. 2002;62(1):209-43. doi: 10.2165/00003495-200262010-00008.
6. Bain B., Brazil M. Adalimumab. Nat Rev Drug Discov. 2003;2:693-4. doi. /10.1038/nrd1182
7. Siddiqui MA, Scott LJ. Infliximab: a review of its use in Crohn's disease and rheumatoid arthritis. Drugs. 2005;65(15):2179-208. doi: 10.2165/00003495-200565150-00014.
8. Mohammadian O, Rajabibazl M, Pourmaleki E, Bayat H, Ahani R, Rahimpour A. Development of an improved lentiviral based vector system for the stable expression of monoclonal antibody in CHO cells. Prep Biochem Biotechnol. 2019;49(8):822-29. doi: 10.1080/10826068.2019.1621893.
9. Gupta K, Parasnis M, Jain R, Dandekar P. Vector-related stratagems for enhanced monoclonal antibody production in mammalian cells. Biotechnol Adv. 2019 Dec;37(8):107415. doi: 10.1016/j.biotechadv.2019.107415.
10. Wurm FM, Wurm MJ. Cloning of CHO cells, productivity and genetic stability—a discussion. Processes. 2017; 5(2):20. doi: 10.3390/pr5020020.
11. Fan Y, Jimenez Del Val I, Muller C, Sen JW, Rasmussen SK, Kontoravdi C, et al. Amino acid and glucose metabolism in fed-batch CHO cell culture affects antibody production and glycosylation. Biotechnology and Bioengineering. 2014 Sep;12:521-35. doi: 10.1002/bit.25450.

12. Xiao Z, Sabourin M, Piras G, Gorfien SF. Screening and optimization of chemically defined media and feeds with integrated and statistical approaches. Methods Mol Biol. 2014;1104:117-35. doi: 10.1007/978-1-62703-733-4_9.

13. Ghafuri-Esfahani A, Shokri R, Sharifi A, Shafiee L, Khosravi R, Kaghazian H, et al. Optimization of parameters affecting on CHO cell culture producing recombinant erythropoietin. Preparative Biochemistry & Biotechnology. 2020 Apr;50(8):834-41. doi: 10.1080/10826068.2020.1753072.

14. Trummer E, Fauland K, Seidinger S, Schriebl K, Lattenmayer C, Kunert R, et al. Process parameter shifting: part I. Effect of DOT, pH, and temperature on the performance of Epo-Fc expressing CHO cells cultivated in controlled batch bioreactors. Biotechnology and Bioengineering. 2006 Aug;94(6):1033-44. doi: org/10.1002/bit.21013.

15. Liu CH, Chen LH. Promotion of recombinant macrophage colony stimulating factor production by dimethyl sulfoxide addition in Chinese hamster ovary cells. J Biosci Bioeng. 2007 Jan;103(1):45-9. doi: 10.1263/jbb.103.45.

16. Ha TK, Kim YG, Lee GM. Effect of lithium chloride on the production and sialylation of Fc-fusion protein in Chinese hamster ovary cell culture. Appl Microbiol Biotechnol. 2014;98:9239-48. doi: 10.1007/s00253-014-6012-0.

17. Jiang Z, Sharfstein ST. Sodium butyrate stimulates monoclonal antibody over-expression in CHO cells by improving gene accessibility. Biotechnol Bioeng. 2008 May 1;100(1):189-94. doi: 10.1002/bit.21726.

18. Park JH, Noh SM, Woo JR, Kim JW, Lee GM. Valeric acid induces cell cycle arrest at G1 phase in CHO cell cultures and improves recombinant antibody productivity. Biotechnol J. 2016 Mar;11(4):487-96. doi: 10.1002/biot.201500327.

19. Yang WC, Lu J, Nguyen NB, Zhang A, Healy NV, Kshirsagar R, et al. Addition of valproic acid to CHO cell fed-batch cultures improves monoclonal antibody titers. Mol Biotechnol. 2014 May;56(5):421-8. doi: 10.1007/s12033-013-9725-x.

20. Toronjo-Urquiza L, James DC, Nagy T, Falconer RJ. Screening naturally occurring phenolic antioxidants for their suitability as additives to CHO cell culture media used to produce monoclonal antibodies. Antioxidants (Basel). 2019 Jun 3;8(6):159. doi: 10.3390/antiox8060159.

21. Chen F, Fan L, Wang J, Zhou Y, Ye Z, Zhao L, et al. Insight into the roles of hypoxanthine and thymidine on cultivating antibody-producing CHO cells: cell growth, antibody production and long-term stability. Appl Microbiol Biotechnol. 2012 Jan;93(1):169-78. doi: 10.1007/s00253-011-3484-z.

22. Du Z, Treiber D, McCarter JD, Fomina-Yadlin D, Saleem RA, McCoy RE, et al. Use of a small molecule cell cycle inhibitor to control cell growth and improve specific productivity

and product quality of recombinant proteins in CHO cell cultures. Biotechnol Bioeng. 2015 Jan;112(1):141-55. doi: 10.1002/bit.25332.

23. Baek E, Lee JS, Lee GM. Untangling the mechanism of 3-methyladenine in enhancing the specific productivity: transcriptome analysis of recombinant Chinese hamster ovary cells treated with 3-methyladenine. Biotechnol Bioeng. 2018 Sep;115(9):2243-54. doi: 10.1002/bit.26777.
24. Arden N, Ahn SH, Vaz W, Rhodes M, Hancock C, Abitorabi MA, et al. Chemical caspase inhibitors enhance cell culture viabilities and protein titer. Biotechnol Prog. 2007;23:506-11. doi: 10.1021/bp060222m.
25. Joshi SD, More Y, Vagdevi HM, Vaidya VP, Gadaginamath GS, Kulkarni VH. Synthesis of new 4-(2,5-dimethylpyrrol-1-yl)/4-pyrrol-1-yl benzoic acid hydrazide analogs and some derived oxadiazole, triazole and pyrrole ring systems: a novel class of potential antibacterial, antifungal and antitubercular agents. Med Chem Res. 2013;22:1073-89. doi: 10.1007/s00044-012-0112-0.
26. Joshi SD, More UA, Dixit SR, Korat HH, Aminabhavi TM, Badiger AM. Synthesis, characterization, biological activity, and 3D-QSAR studies on some novel class of pyrrole derivatives as antitubercular agents. Med Chem Res. 2014;23:1123-47. doi: 10.1007/s00044-013-0709-y.

27. Zhao T, Zhao Z, Lu F, Chang S, Zhang J, Pang J, et al. Two- and three-dimensional QSAR studies on hURAT1 inhibitors with flexible linkers: topomer CoMFA and HQSAR. Mol Divers. 2020;24:141-54. doi: 10.1007/s11030-019-09936-5.
28. Renard JM, Spagnoli R, Mazier C, Salles MF, Mandine E. Evidence that monoclonal antibody production kinetics is related to the integral of the viable cells curve in batch systems. Biotechnol Lett. 1988;10(2):91-6. doi: 10.1007/BF01024632.
29. Chen F, Ye Z, Zhao L, Liu X, Fan L, Tan WS. Correlation of antibody production rate with glucose and lactate metabolism in Chinese hamster ovary cells. Biotechnol Lett. 2012;34:425-32. doi: 10.1007/s10529-011-0798-y.
30. Becker M, Junghans L, Teleki A, Bechmann J, Takors R. The less the better: how suppressed base addition boosts production of monoclonal antibodies with Chinese hamster ovary cells. Front Bioeng Biotechnol. 2019;7:76. doi: 10.3389/fbioe.2019.00076.
31. Hara KY, Kondo A. ATP regulation in bioproduction. Microb Cell Fact. 2015;14:198. doi: 10.1186/s12934-015-0390-6.
32. Lowenstein J. Methods in enzymology, volume 13: citric acid cycle. 1st ed. Boston: Academic Press; 1969.
33. Kay J, Weitzman PD. Krebs citric acid cycle: half a century and still turning. 1st ed. London: Biochemical Society; 1987.

34. Mulukutla BC, Khan S, Lange A, Hu WS. Glucose metabolism in mammalian cell culture: new insights for tweaking vintage pathways. Trends Biotechnol. 2010 Sep;28(9):476-84. doi: 10.1016/j.tibtech.2010.06.005.

35. Hodoniczky J, Zheng YZ, James DC. Control of recombinant monoclonal antibody effector functions by Fc *N*-glycan remodeling in vitro. Biotechnol Prog. 2005;21:1644-52. doi: 10.1021/bp050228w.

36. Liu L. Antibody glycosylation and its impact on the pharmacokinetics and pharmacodynamics of monoclonal antibodies and Fc-fusion proteins. J Pharm Sci. 2015;104(6):1866-84. doi: 10.1002/jps.24444.

37. Simonov V, Ivanov S, Smolov M, Abbasova S, Piskunov A, Poteryaev D. Control of therapeutic IgG antibodies galactosylation during cultivation process and its impact on IgG1/FcγR interaction and ADCC activity. Biologicals. 2019 Mar;58:16-21. doi: 10.1016/j.biologicals.2019.01.002.

38. Thomann M, Reckermann K, Reusch D, Prasser J, Tejada ML. Fc-galactosylation modulates antibody-dependent cellular cytotoxicity of therapeutic antibodies. Mol Immunol. 2016 May;73:69-75. doi: 10.1016/j.molimm.2016.03.002.

39. Thomann M, Schlothauer T, Dashivets T, Malik S, Avenal C, Bulau P, et al. In vitro glycoengineering of IgG1 and its effect on Fc receptor binding and ADCC activity. PLoS One. 2015;10(8):e0134949. doi: 10.1371/journal.pone.0134949.

40. Gramer MJ, Eckblad JJ, Donahue R, Brown J, Shultz C, Vickerman K, et al. Modulation of antibody galactosylation through feeding of uridine, manganese chloride, and galactose. Biotechnol Bioeng. 2011 Jul;108(7):1591-602. doi: 10.1002/bit.23075.

41. Williamson J, Miller J, McLaughlin J, Combs R, Chu C. Scale-dependent manganese leaching from stainless steel impacts terminal galactosylation in monoclonal antibodies. Biotechnol Prog. 2018 Oct;34(5):1290-7. doi: 10.1002/btpr.2662.

42. Prabhu A, Gadre R, Gadgil M. Zinc supplementation decreases galactosylation of recombinant IgG in CHO cells. Appl Microbiol Biotechnol. 2018;102:5989-99. doi: 10.1007/s00253-018-9064-8.

43. Chen P, Harcum SW. Effects of elevated ammonium on glycosylation gene expression in CHO cells. Metab Eng. 2006 Mar;8(2):123-32. doi: 10.1016/j.ymben.2005.10.002.

44. Chen P, Harcum SW. Effects of amino acid additions on ammonium stressed CHO cells. J Biotechnol. 2005 May 25;117(3):277-86. doi: 10.1016/j.jbiotec.2005.02.003.

Supporting Information

S1 Fig. The fed-batch culture profile in the retained glucose condition. Glucose was added on days 8 and 12 to keep the concentration above 1 g/L in the fed-batch cultures. And Feed medium was added on

days 4, 6, and 8. Viable cell density (**A**), viability (**B**), glucose concentration (**C**), and lactate concentration (**D**) were measured every 2 days.

S2 Fig. The trend of viability and cell-specific productivity in each pyrrole derivatives concentration. Viability (A) and cell-specific productivity (B) were analyzed by sampling day-3 cells in the batch cultures. The tested pyrrole derivative concentrations were 0.08 to 0.64 mM in the culture solutions. Culture conditions were considered adequate when viability was above 80%.

S1 Table. The validity of the first screening condition. DMSO was used as the 0% control, and 4-phenyl butyric acid dissolved in DMSO was used as the 100% control. Validity was evaluated at the points of coefficient of variation (CV) using day 3 cell culture solution.

Click here to access/download;Figure;Fig 1.tif

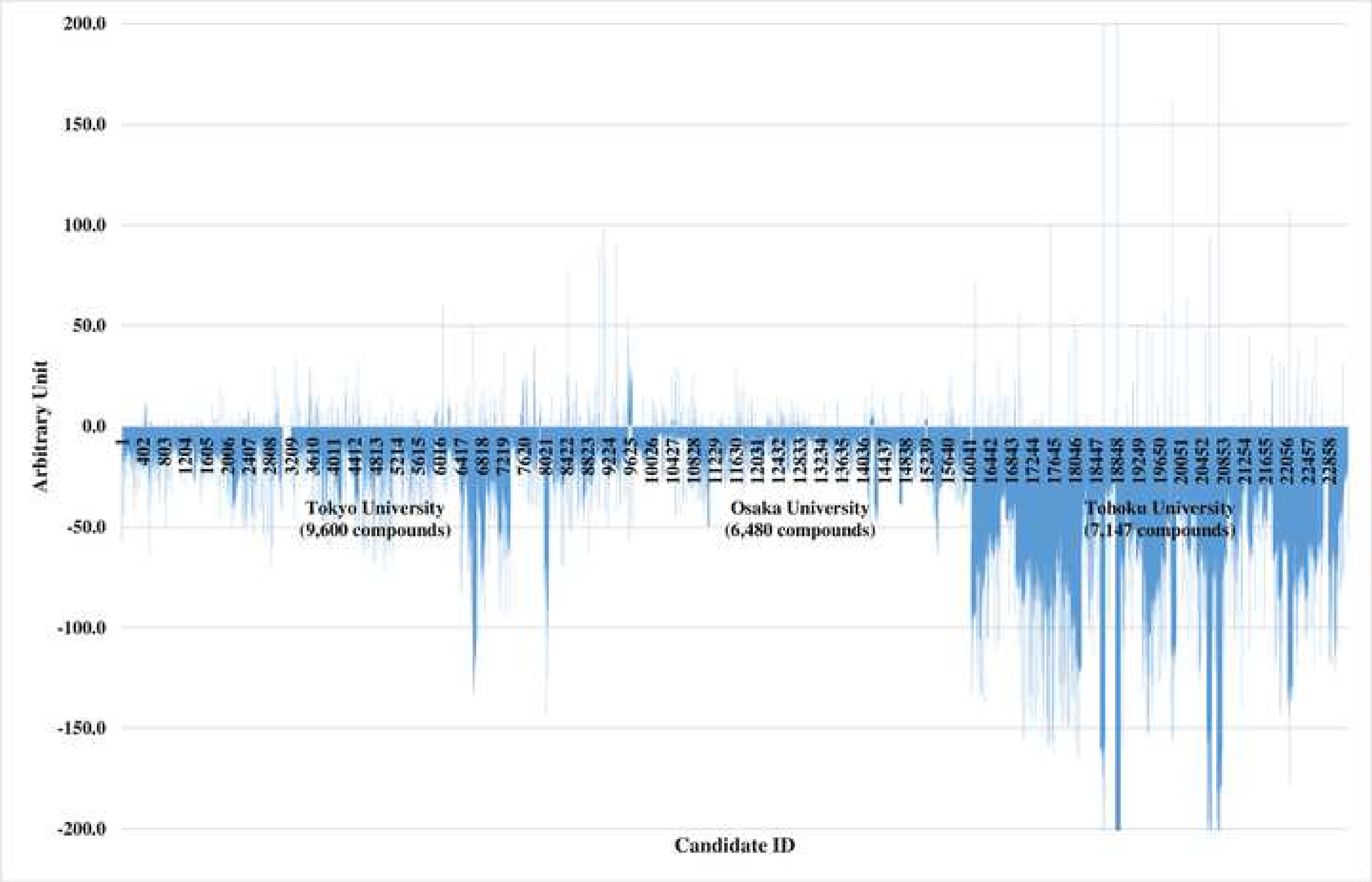

Click here to access/download;Figure;Fig 2.tif

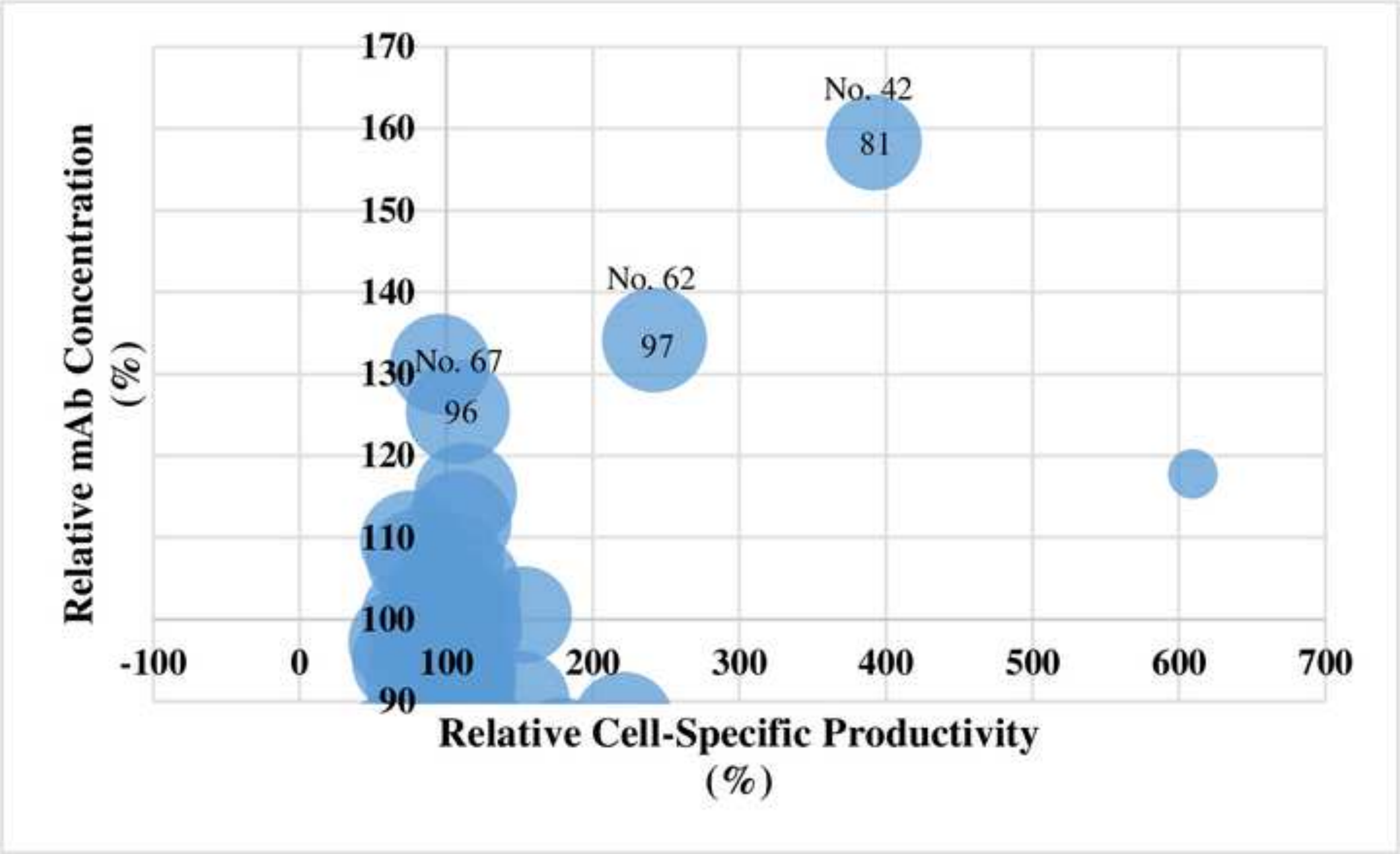

Click here to access/download;Figure;Fig 3.tif

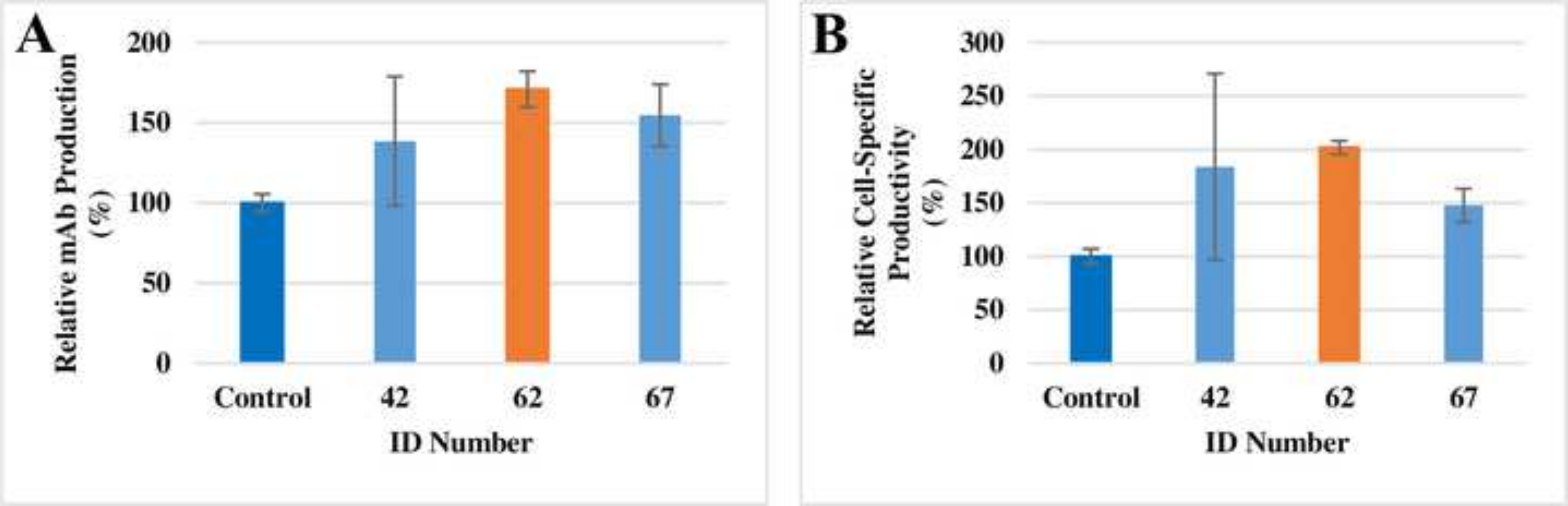

Click here to access/download;Figure;Fig 4.tif

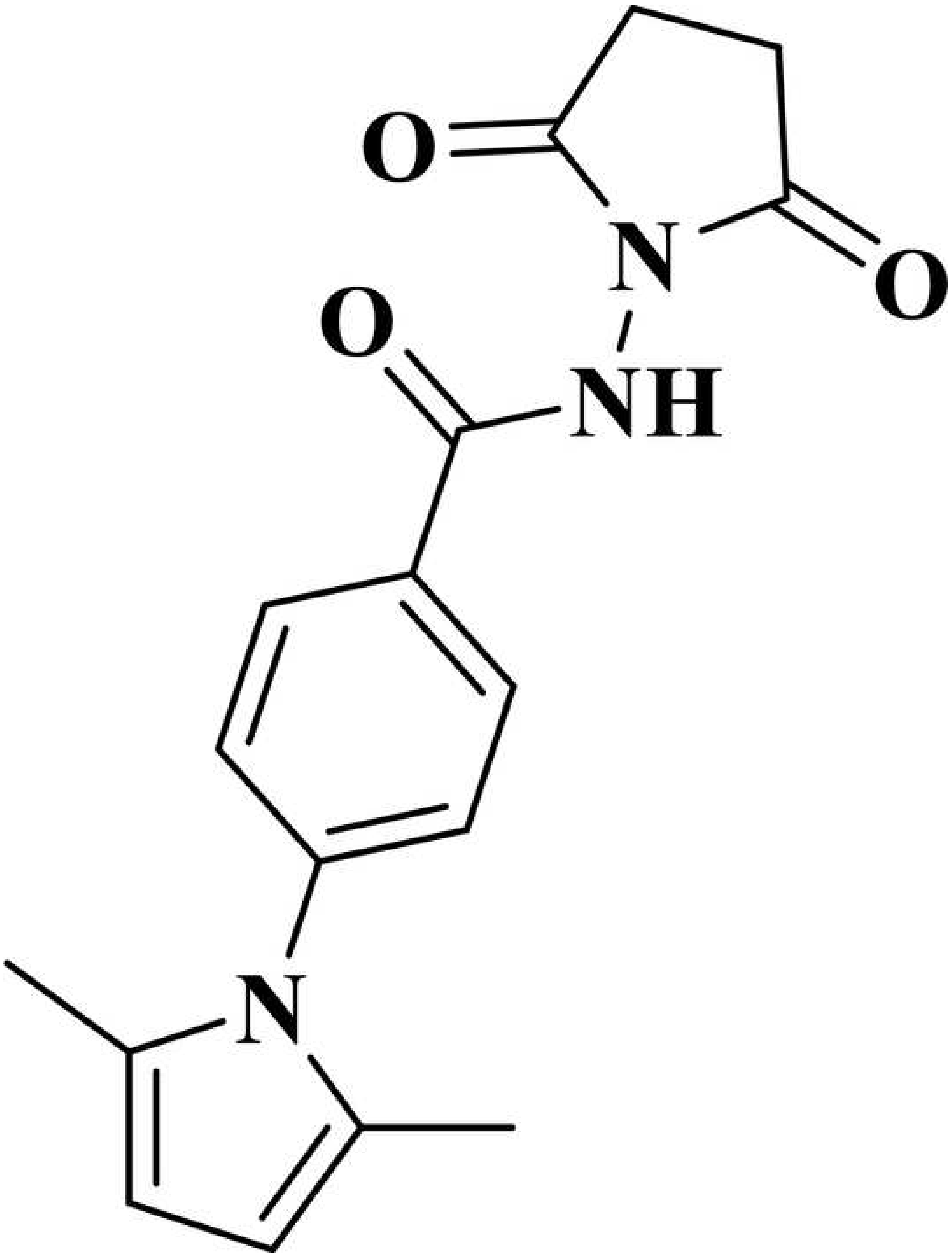

Click here to access/download;Figure;Fig 5.tif

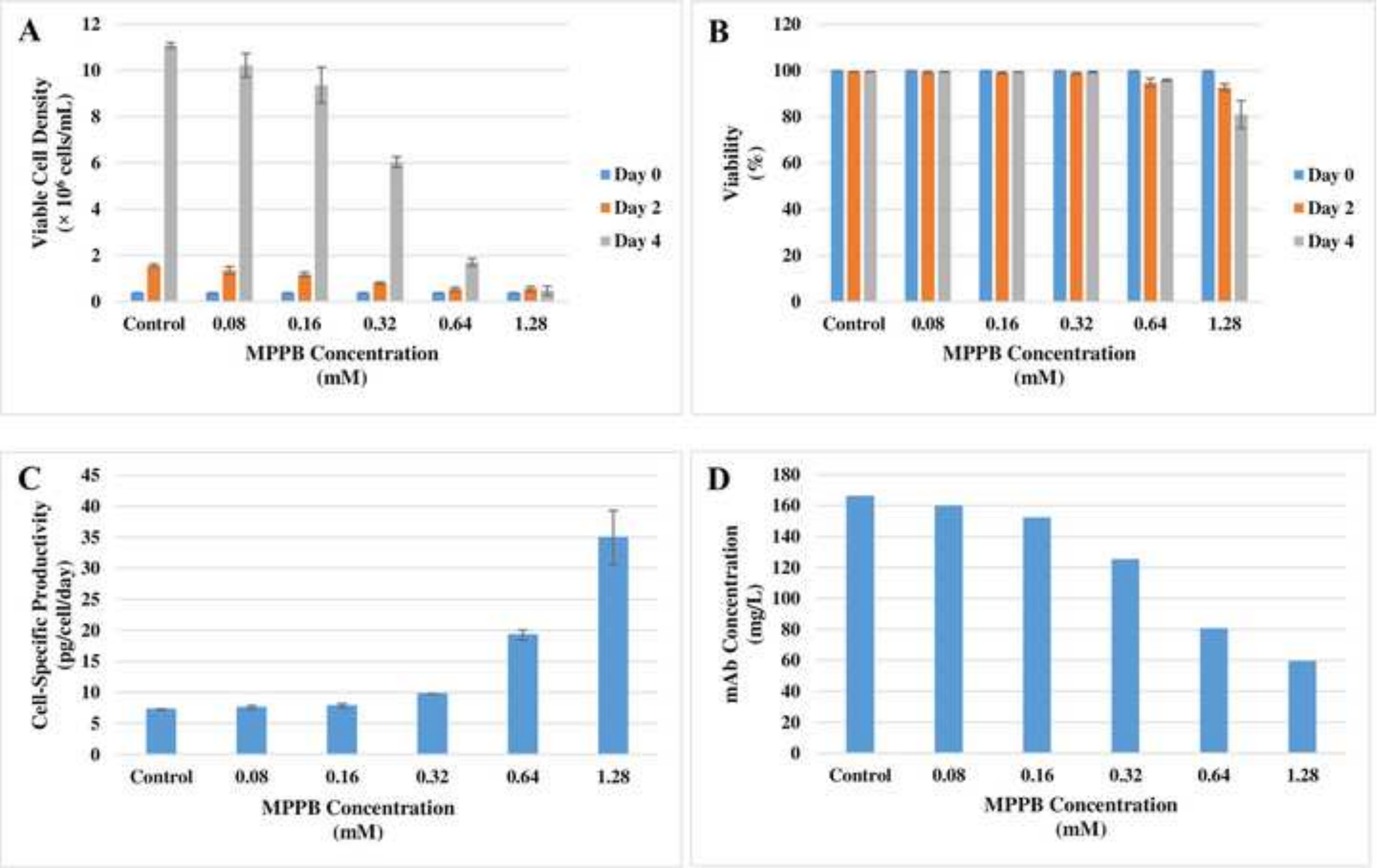

Click here to access/download;Figure;Fig 6.tif

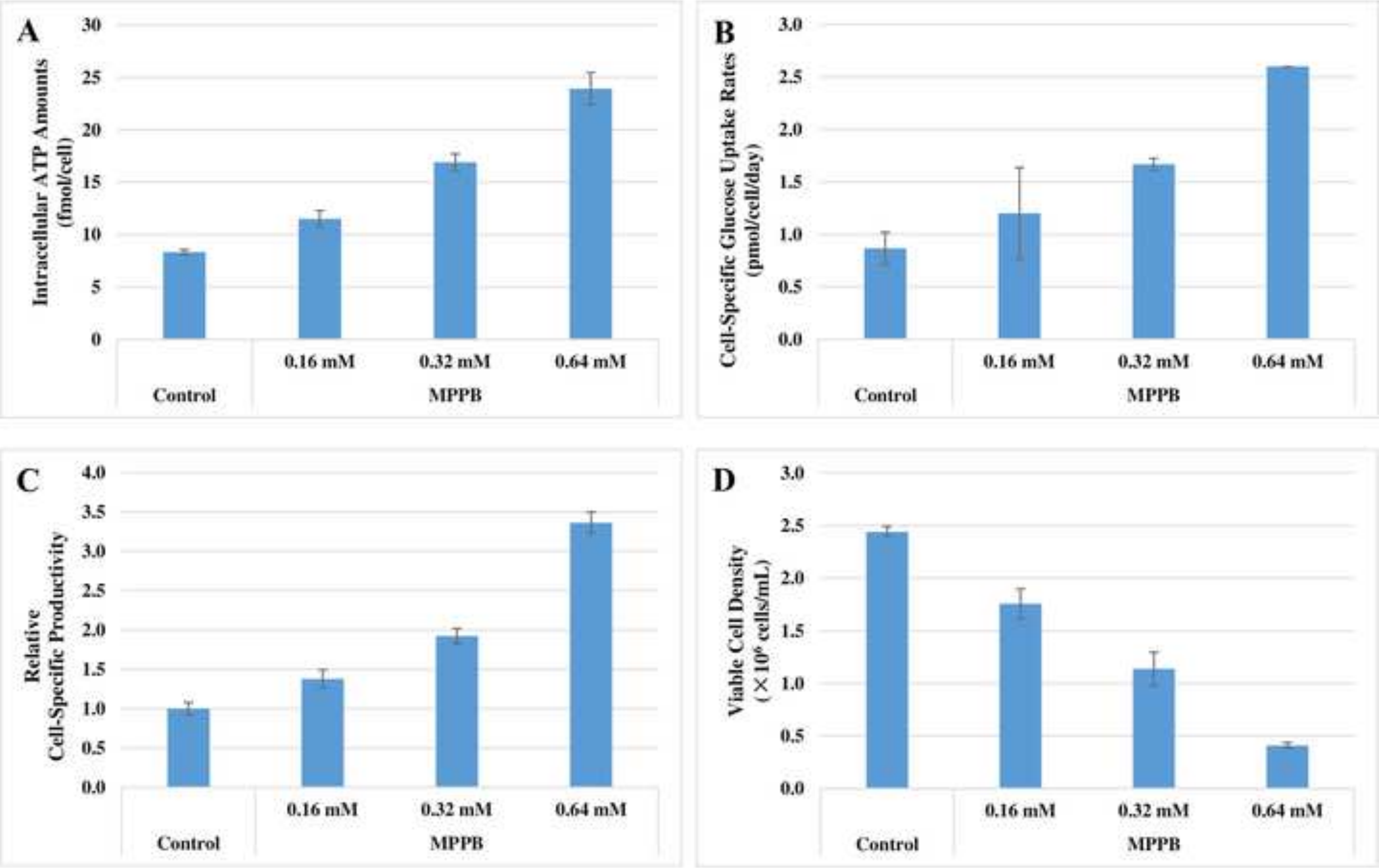

Click here to access/download;Figure;Fig 7.tif

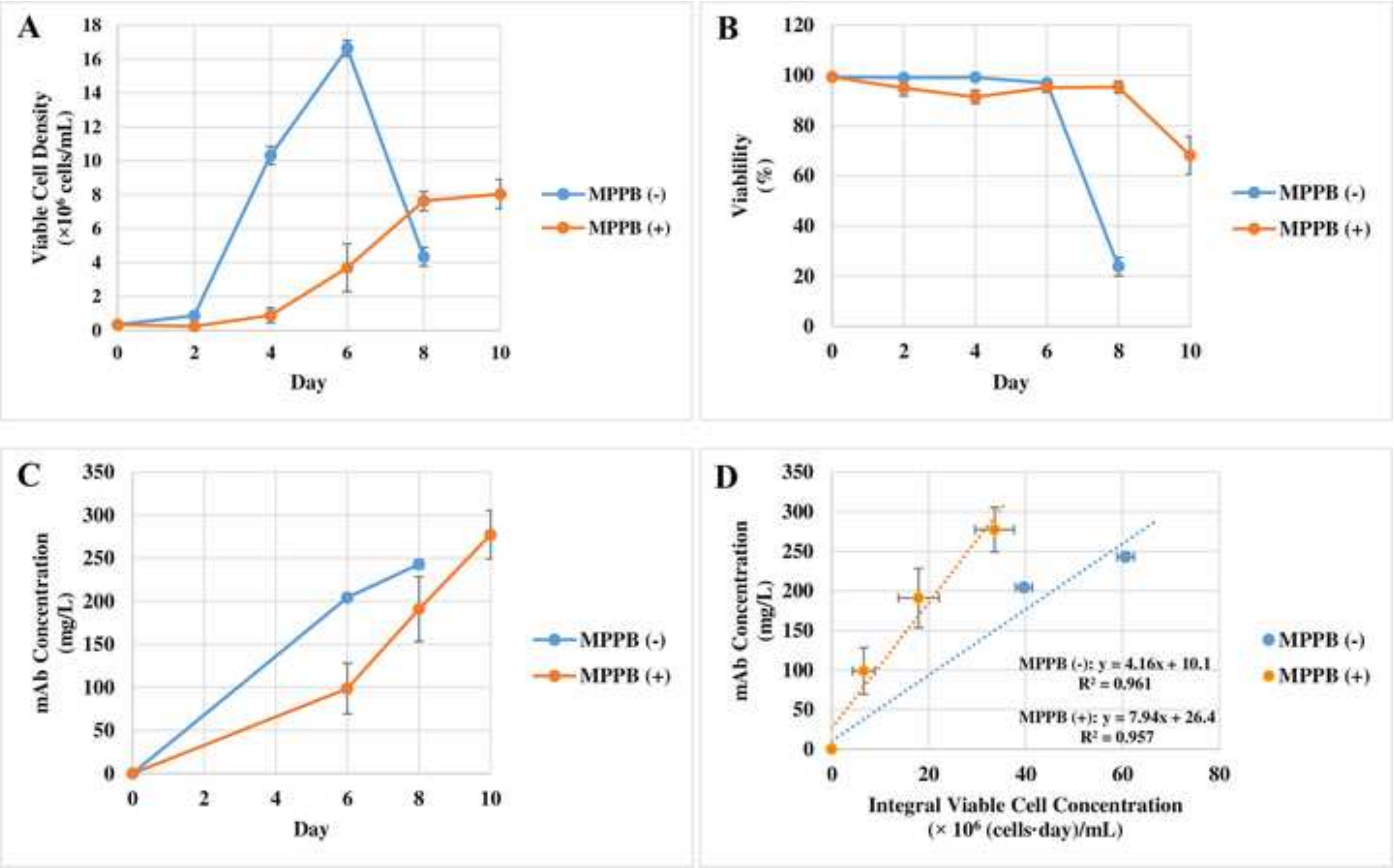

Click here to access/download;Figure;Fig 8.tif

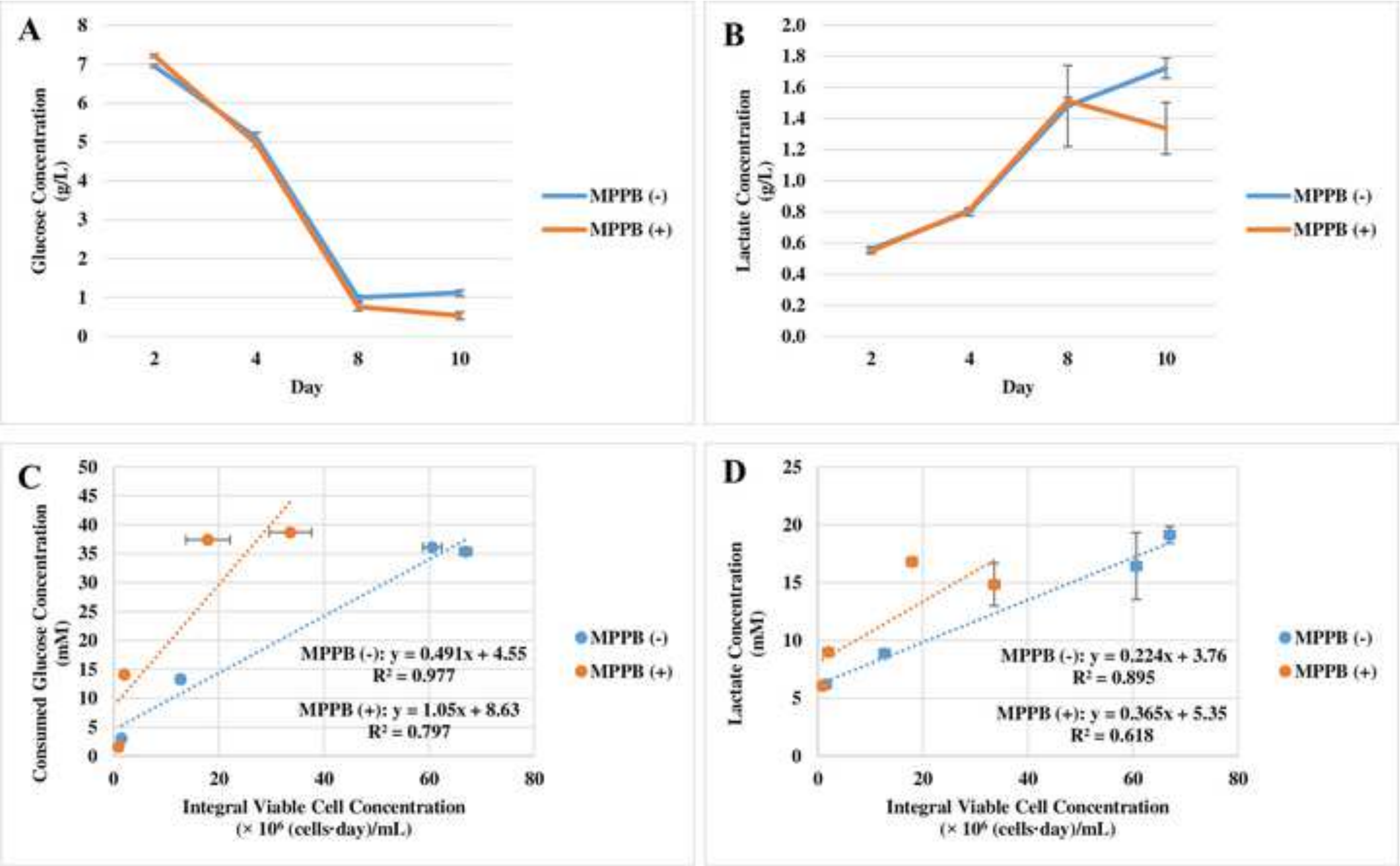

Click here to access/download;Figure;Fig 9.tif

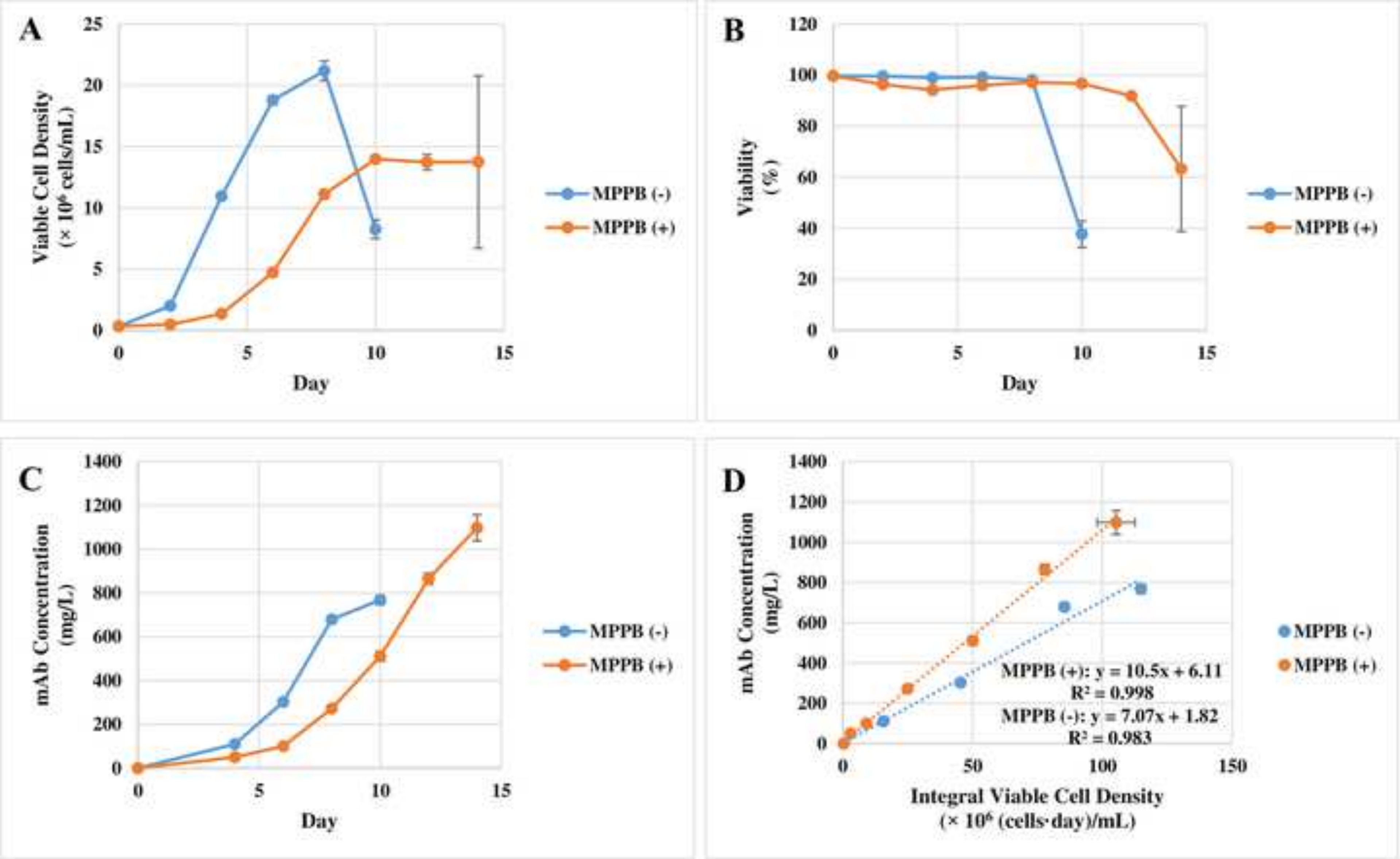

Click here to access/download;Figure;Fig 10.tif

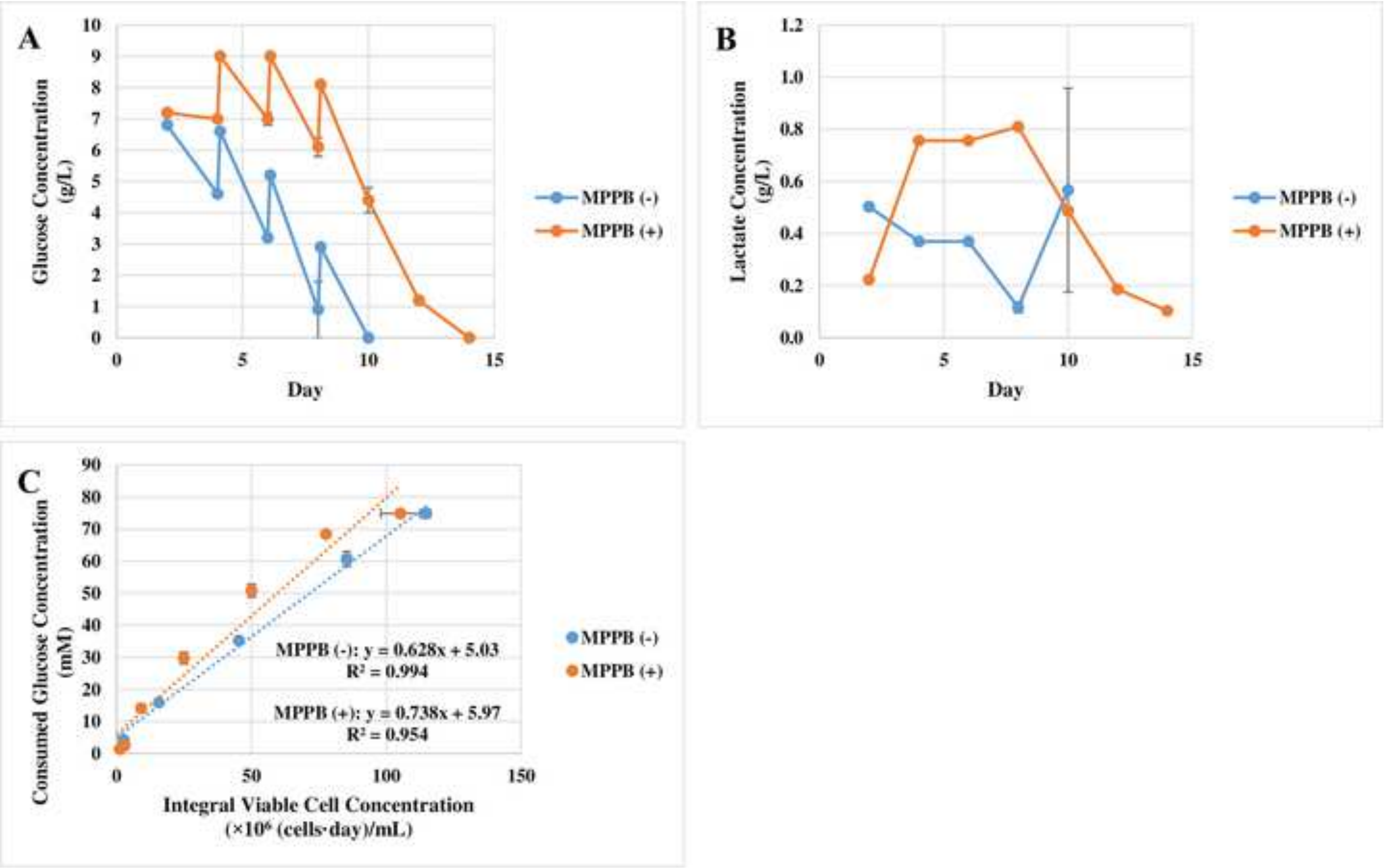

Click here to access/download;Figure;Fig 11.tif

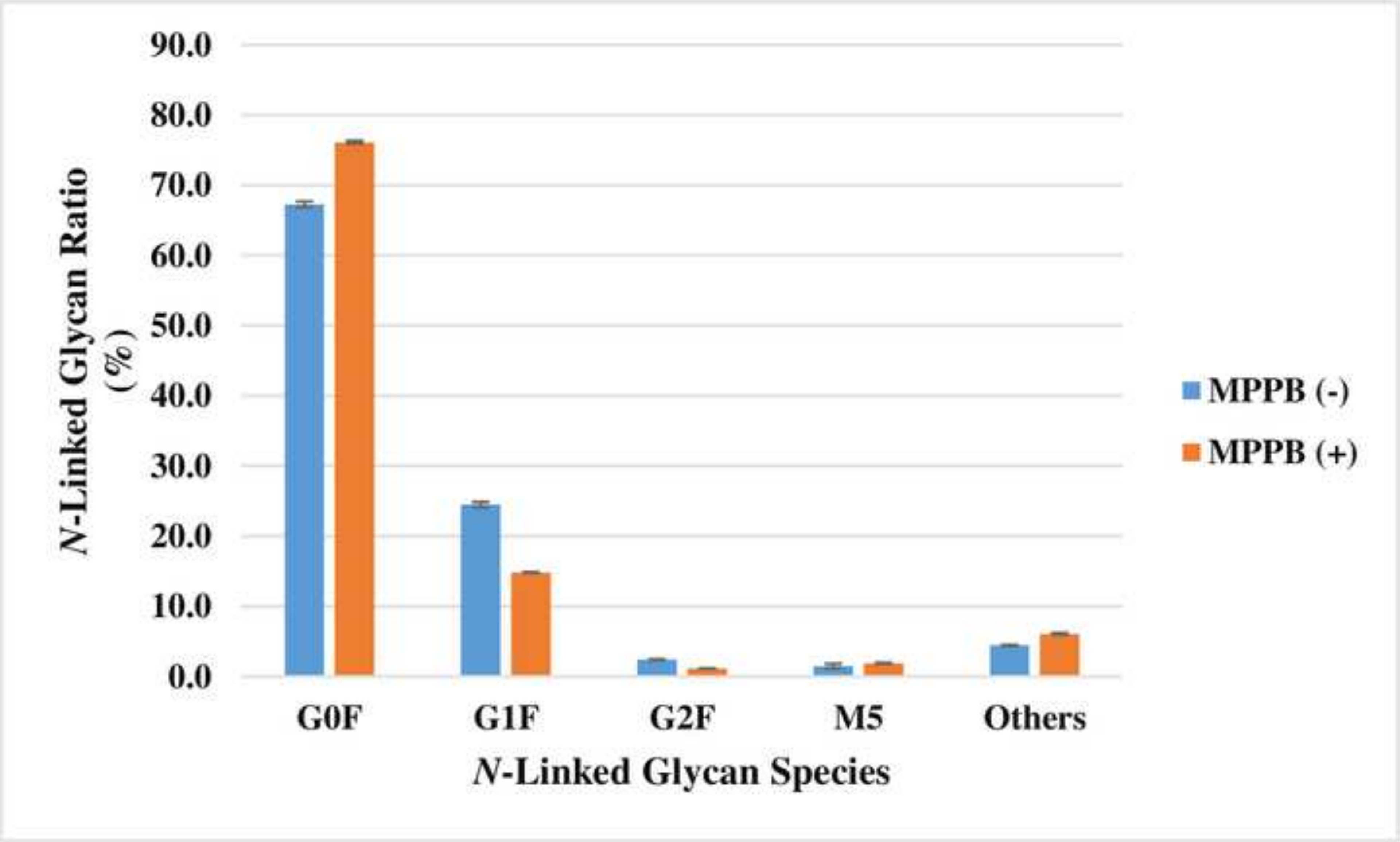

Click here to access/download;Figure;Fig 12.tif

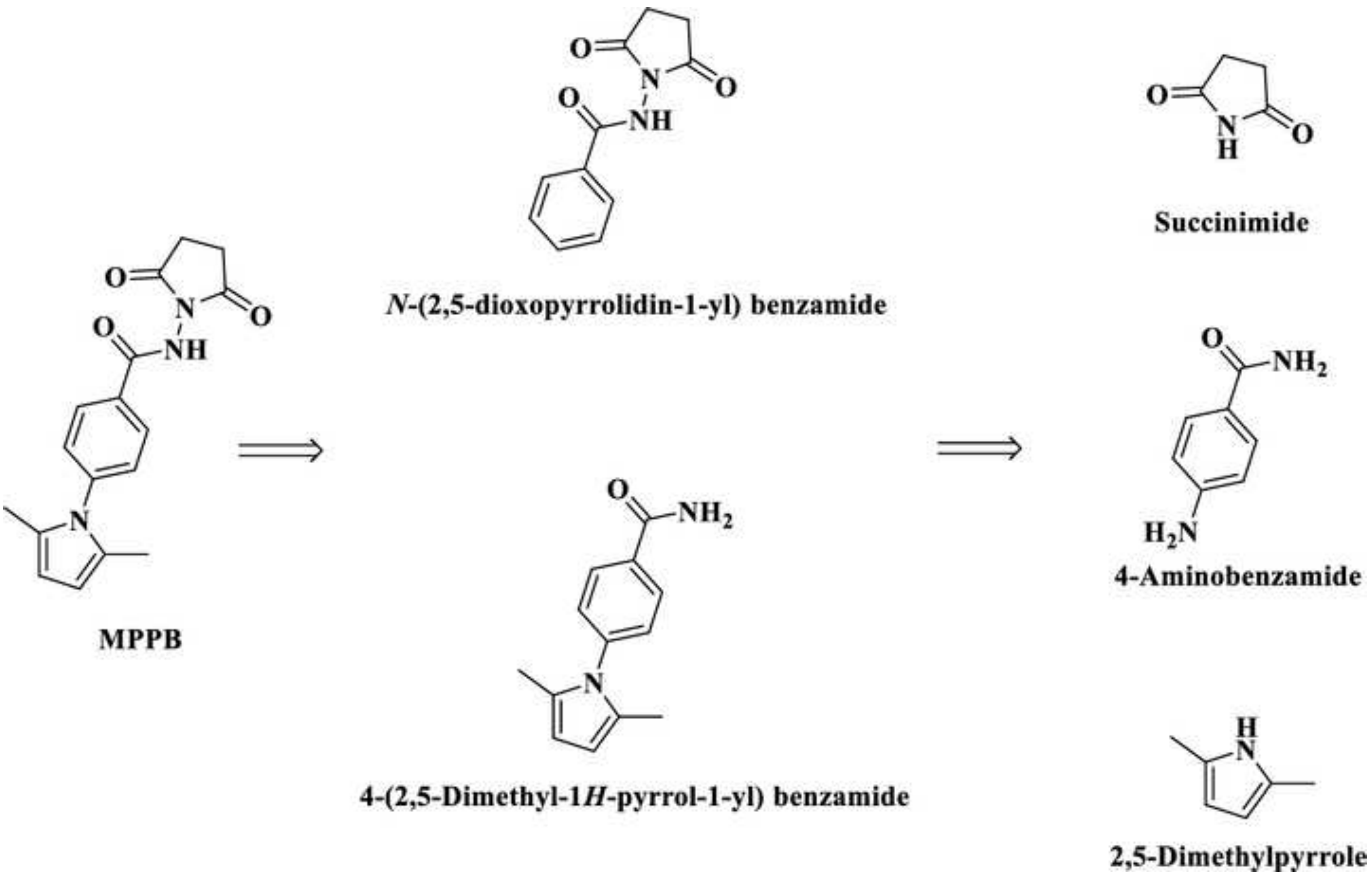

Click here to access/download;Figure;Fig 13.tif

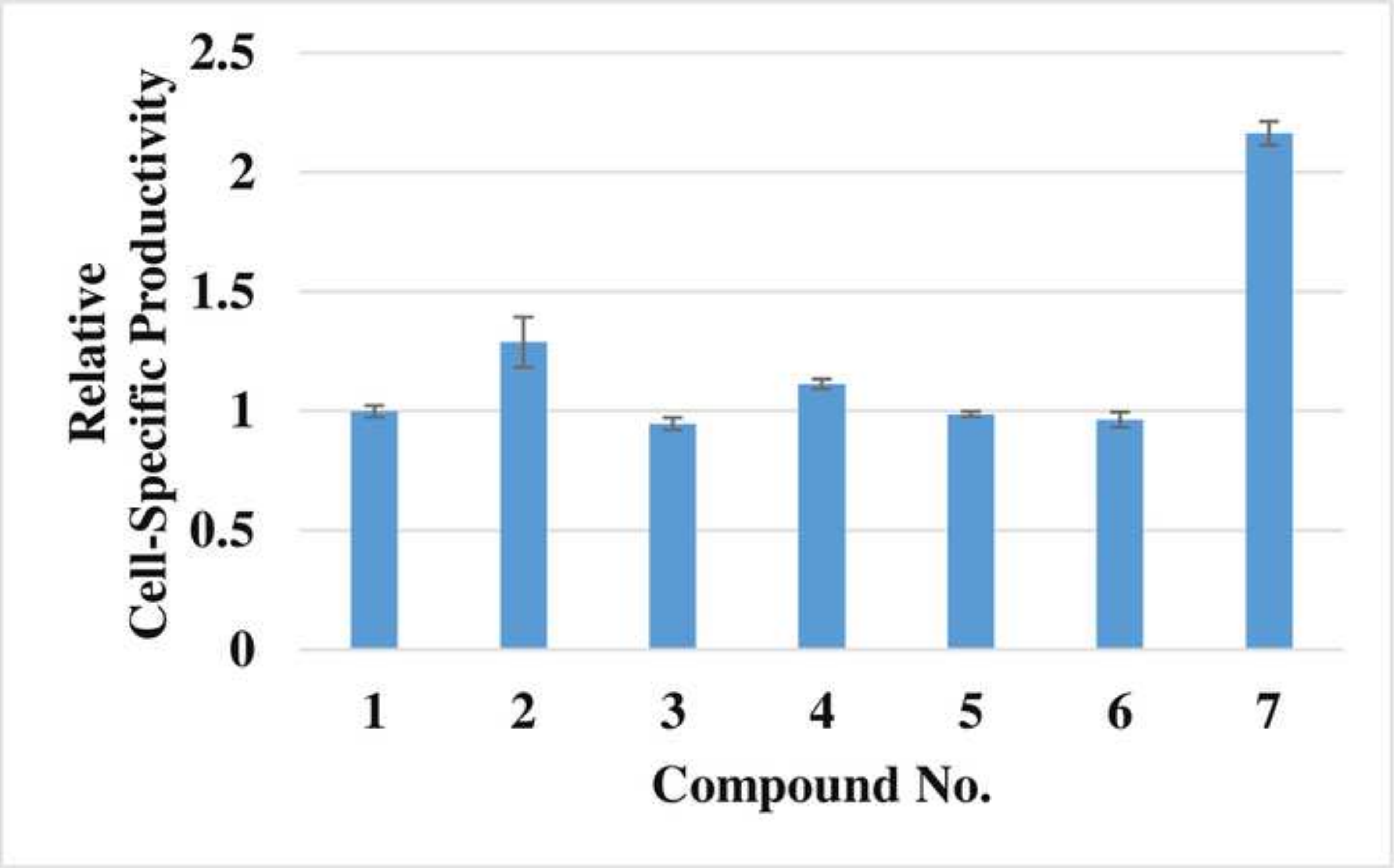

Click here to access/download
Supporting Information
S1 Fig.tif

Click here to access/download
Supporting Information
S1 Table.tif

Click here to access/download
Supporting Information
S2 Fig.tif

Formatted

4-(2,5-Dimethyl-1*H*-pyrrol-1-yl)-*N*-(2,5-dioxopyrrolidin-1-yl) benzamide improves monoclonal antibody production in a Chinese hamster ovary cell culture

A 2,5-dimethylpyrrol derivative improves monoclonal antibody productivity in rCHO cell culture

Yuichi Aki[1,2,¶,*], Yuta Katsumata[1,&], Hirofumi Kakihara[1,&], Koichi Nonaka[1,&], Kenshu Fujiwara[2,¶]

[1] Biologics Technology Research Laboratories, Biologics Division, Daiichi Sankyo Co., Ltd., Chiyoda-machi, Gunma, Japan

[2] Department of Life Science, Akita University, Tegata Gakuen-machi, Akita, Japan

*Corresponding author

E-mail: aki.yuichi.ha@daiichisankyo.co.jp (YA)

[¶]These authors contributed equally to this work.

[&]These authors also contributed equally to this work.

Abstract

There is a continuous demand to improve monoclonal antibody production for medication supply and medical cost reduction. For over 20 years, recombinant Chinese hamster ovary cells have been used as a host in monoclonal antibody production due to robustness, high productivity and ability to produce proteins with ideal glycans ~~because of easy handling~~. Some ~~And it is known that some~~ chemical compounds such as dimethyl sulfoxide, lithium chloride and butyric acid, and so on, have been shown to~~chemical compounds~~ improve monoclonal antibody production in these cell cultures. In this study, we aimed to discover new chemical compounds ~~to~~ that can improve ~~monoclonal~~ cell-specific antibody production ~~effectively~~ in recombinant Chinese hamster ovary cells. ~~During the screening performed for this study, o~~Out of the 23,227 chemicals screened in this study~~compounds~~, 4-(2,5-dimethyl-1*H*-pyrrol-1-yl)-*N*-(2,5-dioxopyrrolidin-1-yl) benzamide was found ~~as an enhancer of~~to increase monoclonal antibody production. The compound has characteristics that suppress cell growth and increase ~~specific glucose consumption~~cell-specific glucose uptake rate ~~–~~ and amount of intracellular adenosine triphosphate during monoclonal antibody production. In addition, the compound also suppressed the galactosylation on a monoclonal antibody, which is a critical quality attribute of therapeutic monoclonal antibodies ~~as pharmaceuticals~~. Therefore, the compound might also be used to control the level of the galactosylation for the ~~as an~~ *N*-linked glycans ~~control reagent and especially as a galactosylation suppression reagent~~. Further, the structure-activity relationship study revealed that 2,5-dimethylpyrrole was the most effective partial structure of 4-(2,5-dimethyl-1*H*-pyrrol-1-yl)-*N*-(2,5-dioxopyrrolidin-1-yl) benzamide on monoclonal

antibody production. Further structural optimization~~ in structure-activity relationship study~~ of 2,5-dimethylpyrrole derivatives could lead to improved production and quality control of monoclonal antibodies.

Introduction

The use of therapeutic monoclonal antibodies (mAb) is becoming more common in the treatment of cancer (with drugs such as pembrolizumab, nivolumab, trastuzumab, and so on) and autoimmune diseases (with drugs such as adalimumab, infliximab, and so on) [1-7].~~Monoclonal antibodyy (mAb) treatment is becoming a common therapeutic treatment for cancer and autoimmune disorders.~~ Pharmaceutical and biologics companies have strongly focused on increasing mAb supply and reducing ~~its~~ manufacturing costs for commercialization and sustainable growth. MAb concentrations of several grams per liter,~~Several gramsliter of~~ produced by using recombinant Chinese hamster ovary (rCHO) cells, which harbor genes encoding for mAb,~~Several grams/liter of production by recombinant Chinese hamster ovary (rCHO) cells, which harbor mAb genes,~~ has been achieved in fed-batch cultures~~ in fed-batch mode cultures~~ through effective host-vector system development [8, 9], genetically engineered host breeding [10], custom media development [11, 12], culture parameter optimization [13, 14], and so on. However, efforts have been continued to establish cost-effective manufacturing processes by reducing the use of expensive materials such as cell culture media and column resins. It is also known that adding chemical compounds in cell cultures improves mAb production. Various chemical compounds such as dimethyl

sulfoxide (DMSO) [15], lithium chloride [162], butyric acid [173], valeric acid [184], valproic acid [195], phenolic antioxidants [206], thymidine [217], and CDK4/6 inhibitor [228] have the potential to enhance mAb production when added to the cell culture at an adequate concentration to control the intracellular state of rCHO cells. In addition, 3-methyladenine up-regulates the unfolded protein response pathway and improves production [239], and compound 7312, which works as a caspase inhibitor, suppresses apoptosis and improves production [2410]. To pursue the possibility of the chemical compound approach, potential synthetic compounds that might enhance mAb production in rCHO cells were gathered from the Basis for Supporting Innovative Drug Discovery and Life Science Research (BINDS) program and evaluated by screening in batch mode cultures. We discoverednewly found that 4-(2,5-dimethyl-1*H*-pyrrol-1-yl)-*N*-(2,5-dioxopyrrolidin-1-yl) benzamide (MPPB), which was developed as an anti-tuberculosis therapeutic compound [2511-2713], stimulated mAb production in cell cultures. To reveal the characteristics of the compound, we investigated cell culture trends and the metabolism of rCHO cells under MPPB-added conditions. These studies were executed in both batch mode and fed-batch mode cultures. The results showed that MPPB improved mAb production in extended cell culture periodsretaining viability, suppressed cell growth, and increased glucose consumptioncell-specific glucose uptake rate and intracellular adenosine triphosphate amounts— in fed -. MPPB not only improved mAbb production, but it also affected the *N*-glycan profile. The structure-activity relationship of MPPB was also evaluated using the compounds corresponding to the partial structures of MPPB as additives in batch cultures and

demonstrated that 2,5-dimethylpyrrole exhibited a strong enhancement of the cell-specific productivity of mAb. ~~The structure-activity relationship of MPPB was also evaluated.~~

Commented [AY/安祐1]: 藤原先生のコメントを基に修正

Materials and M~~m~~ethods

Cell line and cell culture media

Formatted: Font: 16 pt

The tested rCHO cells, which expressed the mAb (Immunoglobulin G1), were generated from CHO-S host cells (Thermo Fisher Scientific, Waltham, MA, USA) by Daiichi Sankyo Co., Ltd. In-house chemically defined media in which L-alanyl-L-glutamine concentration was adjusted to 1 mM was used as expansion and basal media. In-house chemically defined feed media was used in the fed-batch ~~mode~~ cultures.

Chemical compound library

Formatted: Font: 16 pt

The chemical compound library was provided by the University of Tokyo, Osaka University, and Tohoku University, which are members of Basis for Supporting Innovative Drug Discovery and Life Science Research (BINDS) which is a program that supports drug discovery.

Expansion culture condition

Formatted: Font: 16 pt

The rCHO cells were inoculated at a target viable cell concentration of 0.3 × 10^6 cells/mL in 125-mL or 250-mL Erlenmeyer flasks (Corning, Corning, NY, USA) with expansion medium. The working volume was adjusted to 50 mL in the 125-mL flasks and to 100 mL in the 250-mL flasks. The culture solutions were incubated for 3 to 4 days at 37.0 °C in 5% CO_2-enriched air with the shaker set to 120 rpm (20-mm stroke length).

(50 mL in the 125-mL flasks or 100 mL in the 250-mL flasks) and incubated for 3 to 4 days in 5% CO_2-enriched air at 37.0 °C with shaking at 120 rpm (20-mm stroke length).

Formatted: Subscript

Screening of the chemical compound library

Formatted: Font: 16 pt

The rCHO cells were inoculated at a target viable cell concentration of 0.3 × 10^6 cells/mL in CELLSTAR 96-well suspension culture plates (Greiner Bio-One, Frickenhausen, Germany) with expansion medium (198 µL). Rows 1 and 12 on the 96-well plates were used as controls with DMSO (2 µL). On day 0, 2 µL of each chemical compound dissolved in DMSO was added to each well. The plates were incubated at 37.0 °C in 5% CO_2-enriched air with the shaker set to 120 rpm (20-mm stroke length) at 120 rpm (20-mm stroke length). After 3 days of culture, mAb concentrations were analyzed with Octet QKe (ForteBio, Fremont, CA, USA). The resulting mAb concentrations were converted to arbitrary units according to the following equation (1).The resulting mAb concentrations were evaluated as "indicators of mAb concentration," as defined by our team.

$$\text{Arbitrary unit} = (\text{tested mAb concentration} - (\text{average mAb concentration of the controls conditions} + 3\ \text{standard deviations})) \quad (1)$$

Formatted: Caption, Centered, Indent: First line: 0", Line spacing: single

Formatted: Font: Not Bold

Formatted: Font: Times New Roman, Not Italic

An indicator of mAb concentration was calculated as: tested mAb concentration - (average mAb concentration of the control condition + 3 standard deviations). The compounds for which this the indicator arbitrary unit showed a positive value were selected as candidates for the secondary screening. The validity of this screening protocol is shown in S1 Table.

Batch mode culture condition

Formatted: Font: 16 pt

In a secondary screening to evaluate the candidates, rCHO cells were inoculated at a target viable cell concentration of 0.3 × 10^6 cells/mL in 50-mL suspension culture flasks (Greiner Bio-One) with basal medium. The working volume was adjusted to 10 mL. The culture solutions were with 10 mL of basal medium and incubated for 3 days in 5% CO_2-enriched air at 37.0 °C on static culture mode. All tested compounds were dissolved in DMSO at a concentration of 100 mg/mL, and 10 μL (0.1% v/v) of this solution was added to a culture on day 0. In the suspension batch mode cultures, the rCHO cell seeding flasks were incubated for 3 to 10 days in 5% CO_2-enriched air at 37.0 °C with the shaker set to 120 rpm (20-mm stroke length) at 120 rpm (20-mm stroke length).

All tested compounds dissolved in DMSO were added to a culture on day 0.

Fed-batch mode culture condition

Formatted: Font: 16 pt

The rCHO cells were inoculated at a target viable cell concentration of 0.3 × 10^6 cells/mL in 125-mL Erlenmeyer flasks (Corning) with basal medium. The working volume was adjusted to 50 mL. The culture solutions were with 50 mL of expansion medium and incubated at 37.0 °C in 5% CO_2-enriched air

with the shaker set to 120 rpm (20-mm stroke length) until less than 70% viability was reached achievedin 5% CO_2-enriched air at 37.0°C at 120 rpm (20-mm stroke length). On days 4, 6, and 8, feed medium at 2% v/v against the starting volume was added. On day 0, 50 µL (0.1% v/v) of 4-(2,5-dimethyl-1*H*-pyrrol-1-yl)-*N*-(2, 5-dioxopyrrolidin-1-yl) benzamide (Abamachem Ltd., Kyiv, Ukraine) dissolved in DMSO at a concentration of 200 mg/mL was added.

Commented [AY/安祐2]: These sentence were revised according to English editing service.

Measurement of viable cell density and viability

Formatted: Font: 16 pt

Vi-CELL XR (Beckman Coulter, CA, USA) was used to evaluate viable cell density (VCD) and viability according to the manufacturer’s instructions. All testing samples were diluted twice except for on days 0 to 4.

Metabolite analysis

Formatted: Font: 16 pt

Glucose and lactate concentrations in the culturesGlucose consumption and lactate accumulation wereere analyzed by BioProfile FLEX2 (Nova Biomedical, Waltham, MA, USA) according to the manufacturer’s protocol.

Concentration of mAb

Formatted: Font: 16 pt

Cell culture solution was filtrated with a 0.2-µm filter. The mAb concentration in the residual solution was measured by high-performance liquid chromatography (HPLC) with a Protein A affinity

chromatography column, which is a PA ImmunoDetection Sensor Cartridge, with an ID of 2.1 × 30 mm (Applied Biosystems, Bedford, MA, USA).

Measurement of intracellular ATP

An intracellular ATP assay kit (v2, Toyo B-Net, Tokyo, Japan) was used to quantitate ATP amounts in rCHO cells. Each cell culture solution (100 µL) was diluted with phosphate-buffered saline (1 mL) and then centrifuged (450 g, 5 min), and the supernatant was discarded (1 mL). The resulting cell pellet was suspended in phosphate-buffered saline (1 mL) and then centrifuged (450 g, 5 min), and the supernatant was again discarded (1 mL). The cell pellet was suspended in the ATP extraction reagent from the kit (0.5 mL) and incubated for 5 minutes to extract ATP from the cells. The suspension (10 µL) was mixed with luciferase luminescence assay reagents (100 µL), and measurements were obtained by using the Infinite M Plex plate reader (Tecan, Mannedorf, Switzerland). The resulting ATP concentration was converted to the amount of intracellular ATP per cell

Formatted: Font: 16 pt

Formatted: Font: 16 pt

Formatted: Indent: First line: 0 ch

Calculation of cell-specific productivity, cell-specific glucose uptake rates, and cell-specific lactate production rates

Cell-specific productivity (pg/cell/day), cell-specific glucose uptake rates (pmol/cell/day), and cell-specific lactate production rates (pmol/cell/day) were calculated as the slopes of mAb concentration

Formatted: Font: 16 pt, Bold

Formatted: Font: 16 pt

Formatted: Font: 16 pt

Formatted: Font: 16 pt

(mg/L), consumed glucose concentration (mM), and lactate concentration (mM), respectively, to integral viable cell concentration ((cells·day)/mL), according to previous reports [28, 29].

N-linked glycan analysis

The samples purified with the spin column-based antibody purification kit (Cosmo Bio, Tokyo, Japan) according to the manufacturer's protocol were labeled with 2-AB using the EZGlyco mAb-N kit (Sumitomo Bakelite, Tokyo, Japan) for *N*-linked glycan analysis, which was performed under gradient conditions of 50 mM ammonium formate buffer (pH 4.4) on HPLC using a XBridge BEH Amide XP Column (2.5 µm, ID 4.6 × 150 mm, Waters Corporation, Milford, MA, USA) at 60 °C. The detected wavelength was 420 nm, and the flow rate was changed from 0.4 to 1.0 mL/min under gradient conditions.

Statistical analysis

At least 3 batches were executed for statistical analysis. The mean ± standard deviation (SD) and P-value were calculated using JMP statistical analysis software (SAS, NC, USA). Differences in the data were considered significant at $P < 0.05$.

Results

Formatted: Font: 16 pt

Formatted: Font: 16 pt

Initial screening of the chemical compound library

A total of 23,227 chemical compounds were evaluated by cultivation in 96-well plates as an initial screening process (Fig 1). In the rCHO cell cultures, 566 compounds showed a positive effect in terms of increased mAb production. The hit rate was 2.5%. In parallel, we investigated the commercial availability of the first screening positives before starting the second screening to obtain sufficient amounts of the positives for further study. Although 67 chemical compounds were commercially available, another 5 compounds for which the original structures were not commercially available were also tested as derivatives. Therefore, a total of 72 samples underwent the second screening in suspension cultureAs a result, sixty-seven chemical compounds were identified as commercially available. In addition, other five compounds were found to be close similar to commercially available compounds. To conduct further screening, the original five compounds were replaced to the commercially available substitutes. A total of 72 samples underwent a second screening in a suspension culture.

Fig 1. Results of the first screening to evaluate the effect of the chemical compounds on mAb production. Higher mAb production is shown as a positive arbitrary unit.

Second screening of the selected compounds in a suspension culture

Seventy-two chemical compounds were further evaluated in a suspension culture for 3 days.

The relative cell-specific productivity, relative mAb concentration, and viability were assessed to evaluate the chemical compound treatments against the control condition.~~Relative cell-specific productivity, relative mAb concentration, and viability were compared with the control condition. For most of the compounds, the relative mAb concentration was increased with the chemical compound treatment. Therefore, the chemical compounds were screened for relative mAb concentration, relative cell-specific productivity, and viability.~~ The ~~cutoff points for the selection~~selection criteria of the candidate compounds were set to over 120% for relative mAb concentration, 105% for relative cell-specific productivity, and 80% for viability. The samples with the ID numbers 42, 62, and 67 met these criteria, with increases of 125 to 158% for mAb concentration and 108 to 392% for cell-specific productivity in comparison with the control condition (Fig 2).

Fig 2. Relative mAb concentration and cell-specific productivity increased with the addition of the chemical compounds. The numbers in and above the circles indicate viability and the sample's ID number, respectively.

MPPB as an effective additive of mAb production

The three selected compounds had higher~~ values in terms of~~ relative mAb concentration and relative cell-specific productivity. ID number 62 showed an especially strong effect, with a 171% increase in relative mAb concentration and a 202% increase in relative cell-specific productivity compared with the

control condition (Fig 3). As ID number 62 demonstrated the largest effect, we further investigated the characteristics of ID number 62 in batch and fed-batch cultures further.ID number 62 showed higher values than the other tested compounds, so we investigated it further. The selected compound was MPPB (Fig 4), a commercially available substitute for a first-screening-hit-compound supplied from the chemical compound library of the University of Tokyo. MPPB was originally developed as an anti-tuberculosis therapeutic compound [2511-2713].

Fig 3. The effect of the three candidate compounds on relative mAb concentration and relative cell-specific productivity. All cell cultures were executed three times and evaluated on day 3. Each value of relative mAb production (**A**) and relative cell-specific productivity (**B**) were compared statistically.

Fig 4. The structure of 4-(2,5-dimethyl-1*H*-pyrrol-1-yl)-*N*-(2,5-dioxopyrrolidin-1-yl) benzamide (MPPB).

Effect of MPPB on the culture profile in the batch mode cultures

The effect of MPPB on the culture profile was tested in the batch mode cultures, as shown in Fig 5. MPPB suppressed cell growth depending on the concentration of MPPB added (Fig 5A). Viability was slightly decreased at a MPPB concentration of 1.28 mM (Fig 5B) and. cCell-specific productivity was increased by an addition of MPPB with a concentration over 0.32 mM (Fig 5C). MAb concentration decreased with the addition of MPPB due to suppressed cell growth in these short-period batch cultures

Formatted: Font: (Default) Times New Roman

Formatted: Font: (Default) Times New Roman

Formatted: Left, Indent: First line: 0.58"

(Fig 5D). These results showed that treatment with MPPB concentrations of 0.32 to 0.64 mM suppressed cell growth and increased cell-specific productivity while retaining viability. From these resultsTherefore, MPPB concentrations of 0.32 to 0.64 mM were used to clarify the characters ofcharacterize MPPB furtherin following experiments.

Formatted: Left, Indent: First line: 0.58"

Fig 5. MPPB suppresses cell growth and increases cell-specific productivity. The tested MPPB concentration is indicated in the figure. In these tests, VCD viable cell density (**A**), Vviability (**B**), and cell-specific productivity (**C**), and mAb concentration (**D**) were evaluated on day 4.

Formatted: Font: Bold

Becker et al. [30] and Hara and Kondo [31] reported that increased amounts of intracellular ATP are associated with increased cell-specific productivity. Therefore, we investigated intracellular ATP amounts and cell-specific glucose uptake rates, which are related to ATP production [32, 33], in the batch cultures to clarify the reason for the increasing cell-specific productivity (Fig 6). The results showed that both intracellular ATP amounts and cell-specific glucose uptake rates were increased with higher MPPB concentrations. Intracellular ATP amounts increased from 8 to 24 fmol/cell (Fig 6A), and cell-specific glucose uptake rates increased from 0.9 to 2.6 pmol/cell/day (Fig 6B). Intracellular ATP amounts and cell-specific glucose uptake rates were positively correlated with cell-specific productivity (Fig 6C). Additionally, VCD was suppressed with MPPB treatment (Fig 6D)

Fig 6. MPPB increased both intracellular ATP amounts and cell-specific glucose uptake rates and suppressed VCD. Given previous results, the tested MPPB concentrations ranged from 0.32 to 0.64 mM. Intracellular ATP amounts (A), cell-specific glucose uptake rates (B), relative cell-specific productivity (C), and viable cell density (D) were evaluated on day 3.

MPPB was further evaluated in the batch mode cultures. The maximum VCD was reduced from 16.6×10^6 cells/mL to 8.0×10^6 cells/mL with MPPB treatment (Fig 6Fig 7A). On the other hand, there was longer viability under the MPPB-added condition (Fig 6Fig 7B). The mAb concentration under the MPPB-added condition was almost the same value as the control condition at each harvest time point (Fig 6Fig 7C). The MPPB addition increased the cell-specific productivity from 4.2 pg/cell/day to 7.9 pg/cell/day (Fig 6Fig 7D).

Fig 6Fig 7. Cell culture profile and mAb production in rCHO cells treated with MPPB. MPPB at 0.64 mM was added in the batch mode cultures on day 0. VCD Viable cell density (**A**) and viability (**B**) were measured every 2 days. Cell culture was continued until viability below 70% was reached. The slope of concentration of mAb concentration (**C**) toand integral viable cell concentration was used to indicate cell-specific productivitywere used to calculate mAb production (**D**) [2814].

From a spent media analysis, glucose~~ consumption~~ and lactate concentrations~~accumulation~~ showed no differences between the conditions with and without the addition of MPPB (Figs 8~~7~~A and 8~~7~~B). However, ~~specific glucose consumption~~cell-specific glucose uptake rate increased from 0.49 to 1.1 pmol/cell/day with MPPB treatment (Fig 8~~7~~C). In other words, the cell-specific glucose uptake rate under the MPPB-added condition was 0.61 pmol/cell/day higher than that under the control condition. Meanwhile, the cell-specific lactate production rate increased from 0.22 to 0.37 pmol/cell/day under the MPPB-added condition (Fig 8D). The increase in the cell-specific lactate production rate was 0.15 pmol/cell/day, which was smaller than the increase in the cell-specific glucose uptake rate (0.61 pmol/cell/day). This result suggested that consumed glucose was efficiently converted to ATP in the tricarboxylic acid cycle~~ Glucose consumption under the MPPB-added condition was 2.1-fold higher than that under the control condition. Specific lactate accumulation slightly increased from 0.22 to 0.37 pmol/cell/day under the MPPB-added condition (Fig 7D).~~.

Fig 8~~7~~. The effect of MPPB on the metabolite profile in the batch~~ mode~~ cultures. The spent media analysis of glucose concentration (**A**) and lactate concentration (**B**) was conducted every 2 days starting on day 2. ~~Specific glucose consumption~~Cell-specific glucose uptake rates (**C**) and ~~specific lactate accumulation~~cell-specific lactate production rates (**D**) were indicated as the slopes of ~~cumulative~~ consumed glucose ~~consumption~~ concentration and ~~cumulative~~ lactate ~~accumulation~~ concentration, respectively to integral viable cell concentration [28~~14~~, 29~~15~~].

Effect of MPPB on the culture profile in the fed-batch ~~mode~~ cultures

Further evaluation of the MPPB-added condition was continued in the fed-batch ~~mode~~ cultures. The maximum VCD under the MPPB-added condition was reduced from 21.2 × 10^6 cells/mL in the control condition to 14.0 × 10^6 cells/mL (Fig 9~~8~~A). The VCD and viability also decreased after day 8 in the control condition (Figs 9~~8~~A and 9~~8~~B). However, they were kept until day 12 under the MPPB-added condition. Despite suppression of the VCD, the final mAb concentration under the MPPB-added condition reached 1,098 mg/L which was~~and became~~ 1.5 times higher than that under the control condition (Fig 9~~8~~C). The cell-specific productivity under the MPPB-added condition also became 1.5-fold higher than that under the control condition (Fig 9~~8~~D). The cell-specific productivity under the control and MPPB-added conditions was 7.1 and 11 pg/cell/day, respectively.

Fig 9~~8~~. Cell culture profile and mAb production in the fed-batch ~~mode~~ cultures under the MPPB-added condition. MPPB at 0.64 mM was added to a culture on day 0, and 2% (v/v) feed medium was added on days 2, 4, and 6. ~~VCD~~ Viable cell density (**A**) and viability (**B**) were measured every 2 days. The cell culture was continued until viability below 70% was reached. The slope of the mAb concentration (**C**) and integral viable cell concentration were used as cell-specific productivity (**D**) [~~14~~28].

The glucose consumption rate under the control condition was faster than that under the MPPB-added condition, as shown in Fig 9A. Residual glucose was fully consumed by day 10 under the control condition. On the other hand, while, glucose under the MPPB-added condition was retained until day 14 (Fig 10A). The cell-specific glucose uptake rates were 0.63 and 0.74 pmol/cell/day under the control and MPPB-added conditions, respectively (Fig 10C). TThe lactate concentration was kept under 0.8 g/L, and no difference was noted in both conditions (Fig 109B). The specific glucose consumption rates were 0.63 and 0.74 pmol/cell/day under the control and MPPB-added conditions, respectively (Fig 9C).

Fig 109. Metabolite analysis of the fed-batch mode cultures under the MPPB-added condition. Glucose concentration (**A**) and lactate accumulation concentration (**B**) were measured before adding the feed medium. Glucose concentration after adding the feed medium was calculated using the added feed medium amount. Specific glucose consumptionCell-specific glucose uptake rates (**C**) was indicated as thea slope of cumulative consumed glucose consumption concentration against integral viable cell concentration [2814, 2915].

In addition, major *N*-linked glycans (G0F, G1F, G2F, and M5) on mAb were investigated on day 14 under the MPPB-added condition. G0F was the major *N*-linked glycan, and G1F was decreased from 24.5 to 14.8% under the MPPB-added condition, although G2F and M5 ratios were not changed, as shown in Fig 101.

Fig 11~~0~~. MPPB suppresses galactosylation of mAb. Each value was calculated as the percentage of total *N*-linked glycans. The ratio was statically analyzed.

Structure-activity relationship of MPPB

The MPPB structure is divided into five chemical components, as follows: *N*-(2,5-dioxopyrrolidin-1-yl) benzamide; 4-(2,5-dimethyl-1*H*-pyrrol-1-yl) benzamide; succinimide; 4-aminobenzamide; and 2,5-dimethylpyrrole (Fig 12~~1~~).

Fig 12~~1~~. Five chemical components derived from MPPB.

The activity of the five chemical components derived from MPPB was identified and compared with that of MPPB in terms of cell-specific productivity (Fig 13~~2~~). 4-(2,5-Dimethyl-1*H*-pyrrol-1-yl) benzamide and 2,5-dimethylpyrrole increased cell-specific productivity. Further, the 2,5-dimethylpyrrole-added condition had a 2.2-fold higher cell-specific productivity than the control condition.

Fig 13~~2~~. Evaluation of the effect of the five chemical components derived from MPPB on cell-specific productivity. Each chemical compound was added on day 0 and evaluated on day 3. The tested concentration of each chemical compound was 0.32 mM.

Lane 1: dimethyl sulfoxide (control); lane 2: MPPB; lane 3: *N*-(2,5-dioxopyrrolidin-1-yl) benzamide; lane 4: 4-(2,5-dimethyl-1*H*-pyrrol-1-yl) benzamide; lane 5: succinimide; lane 6: 4-aminobenzamide; lane 7: 2,5-dimethylpyrrole.

Further evaluation was conducted to identify the effect of pyrrole derivatives on viability and cell-specific productivity in the batch mode cultures (Table 1). Cell-specific productivity with alkyl pyrrole derivatives (**2**, **9-14**) was increased up to 1.4- to 7.8-fold higher than that with the control condition, although pyrrole (**7**) and 1-alkyl pyrroles (**1**, **8**) did not show any activity. On the other hand, viability decreased below 50% with these alkyl pyrrole derivatives (**2**, **9-14**), except for 2,5-dimethylpyrrole (**13**). 2,5-Dimethylpyrrolidine (**6**), which has a reduced framework of 2,5-dimethylpyrrole (**13**), also did not affect the cell culture. Thus, 2,5-dimethylpyrrole (**13**) was found to be the most effective chemical structure to increase cell-specific productivity while retaining viability in the 0.32 mM concentration. In addition, alkyl-pyrroles (**9**-**14**) that showed high cell-specific productivity in the 0.32 mM concentration were tested to clarify which pyrrole derivative was the best compound in terms of increasing cell-specific productivity and retaining viability. The results showed that 2,5-dimethylpyrrole (**13**) effectively increased cell-specific productivity while retaining viability (S2 Fig)Thus, 2,5-Dimethyl pyrrole (**13**) was found to be the most effective chemical structure to increase mAb production in a tested rCHO cell culture..

Formatted: Font: Bold

Formatted: Font: Bold

Formatted: Font: Bold

Formatted: Font: Bold

Table 1. Effect of pyrrole derivatives on viability and relative cell-specific productivity in rCHO cell cultures.

Compound No.	Compound name	Viability (%)	Relative cell-specific produc~~tion rate~~tivity
-	Dimethyl sulfoxide (control)	99.4 (0.4)	1.0 (0.0)
-	MPPB	99.2 (0.5)	1.4 (0.2)
**1**	1-(2-Hydroxyethyl)pyrrole	99.6 (0.2)	1.0 (0.1)
**2**	2-Ethylpyrrole	41.2 (2.5)	1.4 (0.1)
**3**	Pyrrole-2-carboxaldehyde	99.3 (0.2)	1.0 (0.1)
**4**	Pyrrole-2-carboxylic acid	99.5 (0.2)	1.0 (0.1)
**5**	Pyrrolidine	98.9 (1.4)	1.0 (0.0)
**6**	2,5-Dimethylpyrrolidine	99.0 (1.0)	1.0 (0.0)
**7**	Pyrrole	99.3 (0.7)	1.0 (0.1)
**8**	1-Methylpyrrole	99.4 (0.1)	1.0 (0.1)
**9**	2-Methylpyrrole	50.2 (0.2)	3.0 (0.2)
**10**	3-Methylpyrrole	34.4 (2.0)	4.0 (0.2)
**11**	2,3-Dimethylpyrrole	2.6 (1.7)	7.8 (0.4)
**12**	2,4-Dimethylpyrrole	30.7 (3.4)	4.8 (0.3)
**13**	2,5-Dimethylpyrrole	97.9 (0.6)	2.2 (0.0)
**14**	3,4-Dimethylpyrrole	4.0 (1.2)	7.4 (0.3)

Formatted Table

Each pyrrole derivative was added on day 0 and evaluated on day 3. The tested concentration of each pyrrole derivative was 0.32 mM. Standard deviation was indicated in the blanket.

Discussion

In this study, we screened chemical compounds to find novel additives that improve mAb production in rCHO cells and thereby promote pharmaceutical supply (Figs 1-3). MPPB had 1.5-fold

higher mAb production than the control conditions in the fed-batch~~ mode~~ cultures. This is the first study in which MPPB was applied in rCHO cell cultures to improve mAb production.

Our study revealed two key factors for the improvement of mAb production by MPPB. One wa~~i~~s the ~~extension of cell culture period (Figs 6A and 8A)~~retaining viability (Figs 7B and 9B), and the other wa~~i~~s the increase of cell-specific productivity (Figs 7~~6~~D and 9~~8~~D). It is likely that these two factors explain the increased mAb production in the MPPB-added condition despite lower cell concentrations compared to the control condition.

The mechanism of~~The reason why~~ MPPB in terms of ~~extended~~ retaining viability~~cell culture periods~~ is unclear. MPPB might keep glucose concentrations high, leading to ~~extended cell culture periods~~retained viability (Figs 9~~8~~B~~A~~ and 10~~9~~A). However, although the remaining glucose concentration was almost similar with and without the addition of MPPB in the batch ~~mode~~ cultures, the viability remained high under the MPPB-added condition (Figs 7~~6~~B and 8~~7~~A).~~ Additionally,~~ O~~o~~nce, ~~we kept a~~ glucose ~~concentration~~ was maintained above 1 g/L in the fed-batch~~ mode~~ cultures (S1 Fig), the viability under the control condition –was 45% lower than that under the MPPB-added condition on day 12. These results suggested that cell growth suppression by MPPB treatment prevented the depletion of medium components except for glucose, resulting in maintained viability~~These results indicate that MPPB worked on any intracellular functions that keep cells alive or suppress the exhaustion of unidentified medium ingredients to maintain viability~~.–

Commented [AY/安祐3]: This sentence was edited by Scientific language Co., Ltd.

The cause of increased cell-specific productivity, however, is clearer. In our study, MPPB treatment had two important effects to increase cell-specific productivity. One was the suppression of cell growth (Figs 5A, 6D, 7~~6~~A and 9~~8~~A), and the other was the acceleration of ~~specific glucose consumption~~cell-specific glucose uptake rates (Figs 6B, 8C and 10C), while keeping cell-specific lactate production rates low (Fig 8D). Both the effects would control the amounts of intracellular ~~adenosine triphosphates (~~ATP~~s)~~. As reported by Mulukutla et al., growing cells require more ATPs than quiescent cells [34~~16~~]. An ~~Increased~~ increased ~~specific glucose consumption~~cell-specific glucose uptake rate ~~(Figs 7C and 9C)~~ ~~would~~ leads to an increased amounts of intracellular ATP when ~~since~~ glucose ~~is~~ is ~~commonly~~ metabolized to ATP in the tricarboxylic acid cycle [32~~17~~, 33~~18~~], but not when glucose is metabolized to lactate. Becker et al. [30~~19~~] and Hara and Kondo [~~20~~31] have reported that the energy available in the form of intracellular ATP is crucial for mAb production. Accordingly, it may be hypothesized that rCHO cells increased intracellular ATP amounts by suppressing cell growth and increasing ~~glucose consumption~~cell-specific glucose uptake rates, thereby increasing cell-specific productivity, as expected. To test this hypothesis, we ~~preliminary~~ investigated ~~intracellular ATP amounts~~the relationship between the amount of intracellular ATP and MPPB concentration (Fig 6). We found that the amount of intracellular ATP ~~amounts~~ was~~ere~~ ~~highly~~ enhanced ~~(6.4-fold) under the MPPB-added condition~~with higher MPPB concentrations (~~S2~~ Fig 6A). Cell-specific glucose uptake rates and cell-specific productivity were also increased (Figs 6B and 6C). On the other hand,~~and~~ VCD was also suppressed with higher MPPB conditions (Fig 6D). Therefore, we predicted that the increased cell-specific productivity under the MPPB-added condition

Formatted: Not Highlight

Formatted: Font: 10.5 pt

might occur due to the increasing amount~~s~~ of intracellular ATP resulting from cell growth suppression and enhanced cell-specific glucose uptake rates. Our results showed that a chemical compound such as MPPB, which suppresse~~d~~s cell growth and increase~~d~~s intracellular ATP amounts, may improve mAb production in rCHO cell cultures.

Furthermore, we found that MPPB treatment suppressed galactosylation in *N*-linked glycans on the expressed mAb (Fig 1~~1~~0). The galactosylation ratio in *N*-linked glycans on mAb is one of the critical quality attributes of mAb in terms of pharmaceutical development, because the galactosylation profile affects complement-dependent cytotoxicity [~~21~~35, 36~~22~~] and antibody-dependent cellular cytotoxicity [37~~23, 24, ~~39~~25~~]. Therefore, to adjust mAb activity and maintain consistent quality, galactosylation is often controlled during pharmaceutical process research and development. Some media additives are known to affect the galactosylation of mAb in rCHO cells. For example, valeric acid, valproic acid, CDK4/6 inhibitor, galactose, manganese, and uridine encourage galactosylation [18, 19, 22, 40, 41~~8, 26, 27~~], while zinc and ammonia suppress galactosylation [42~~28~~, 43~~29~~]. However, ammonia is harmful to rCHO cells [44~~30~~]. The number of effective galactsylation suppressants is small, and, therefore, the suppression of galactosylation by media additives is not well understood. Our findings that MPPB suppressed the galactosylation may offer a new option to control the quality of mAb by the suppression of gal~~r~~actosylation in the *N*-linked glycans of mAb.

Finally, the structure-activity relationship study revealed that the most critical chemical portion of MPPB to maintain viability and improve cell-specific productivity of mAb in rCHO cells is 2,5-

dimethyl-pyrrole (Fig 132, Table 1). Since 2,5-dimethyl-pyrrolidine having a reduced framework of 2,5-dimethyl-pyrrole showed no effect, the importance of the heteroaromatic framework of 2,5-dimethyl-pyrrole for both the activities was suggested. The 2,5-dialkyl substitution of pyrrole was also indispensable. The viability was extremely decreased in the cases of other mono- or bis-alkyl-substituted pyrroles. Furthermore, non-alkylated or *N*-substituted pyrroles showed no effect on both the viability and the cell-specific productivity. Thus, the 2,5-dialkyl-substituted pyrrole framework was found to be effective on maintaining viability and improving cell-specific productivity of mAb in rCHO cells during the structure-activity relationship study. The mechanism of the action of MPPB and 2,5-dimethyl-pyrrole in rCHO cell cultures is currently being considered for future application studies.

Acknowledgments

The authors acknowledge the support provided by Akihiro Kawara (Daiichi Sankyo Co., Ltd.) in terms of contracting with the BINDS program, and by Masami Katase (Daiichi Sankyo Co., Ltd.) in terms of collecting commercially available chemical compounds.

Formatted: Font: 10.5 pt, Not Bold

~~Acknowledgments~~

This research was supported by the Platform Project for Supporting Drug Discovery and Life Science Research (Basis for Supporting Innovative Drug Discovery and Life Science Research [BINDS]) from Japan Agency for Medical Research and Development (AMED) under grant numbers JP19am0101086, JP19am0101084, and JP19am0101095 (support numbers 1009, 0847, and 1011).

References

Commented [AY/安祐4]: I ensured your references abide by the guidelines given by *PLOS ONE*. *PLOS ONE* uses the numbered citation (citation-sequence) method and first six authors followed by “et al.”

1. Pento JT. Monoclonal antibodies for the treatment of cancer. Anticancer Res. 2017 Nov;37(11):5935-9. doi: 10.21873/anticanres.12040.
2. Yoo SM, Chung SH. Targets of monoclonal antibodies for immunological diseases. Arch. Pharm. Res. 2019;42:293-304. doi: 10.1007/s12272-018-1087-2.
3. Qu J, Wang L, Jiang M, Zhao D, Wang Y, Zhang F, et al. A review about pembrolizumab in first-line treatment of advanced NSCLC: focus on KEYNOTE studies. Cancer Manag Res. 2020;12:6493-509. doi: 10.2147/CMAR.S257188.
4. Keating GM. Nivolumab: a review in advanced nonsquamous non-small cell lung cancer. Drugs. 2016 Jun;76(9):969-78. doi: 10.1007/s40265-016-0589-9.
5. McKeage K, Perry CM. Trastuzumab: a review of its use in the treatment of metastatic breast cancer overexpressing HER2. Drugs. 2002;62(1):209-43. doi: 10.2165/00003495-200262010-00008.
6. Bain B., Brazil M. Adalimumab. Nat Rev Drug Discov. 2003;2:693-4. doi. /10.1038/nrd1182

7. Siddiqui MA, Scott LJ. Infliximab: a review of its use in Crohn's disease and rheumatoid arthritis. Drugs. 2005;65(15):2179-208. doi: 10.2165/00003495-200565150-00014.

8. Mohammadian O, Rajabibazl M, Pourmaleki E, Bayat H, Ahani R, Rahimpour A. Development of an improved lentiviral based vector system for the stable expression of monoclonal antibody in CHO cells. Prep Biochem Biotechnol. 2019;49(8):822-29. doi: 10.1080/10826068.2019.1621893.

9. Gupta K, Parasnis M, Jain R, Dandekar P. Vector-related stratagems for enhanced monoclonal antibody production in mammalian cells. Biotechnol Adv. 2019 Dec;37(8):107415. doi: 10.1016/j.biotechadv.2019.107415.

10. Wurm FM, Wurm MJ. Cloning of CHO cells, productivity and genetic stability—a discussion. Processes. 2017; 5(2):20. doi: 10.3390/pr5020020.

11. Fan Y, Jimenez Del Val I, Muller C, Sen JW, Rasmussen SK, Kontoravdi C, et al. Amino acid and glucose metabolism in fed-batch CHO cell culture affects antibody production and glycosylation. Biotechnology and Bioengineering. 2014 Sep;12:521-35. doi: 10.1002/bit.25450.

12. Xiao Z, Sabourin M, Piras G, Gorfien SF. Screening and optimization of chemically defined media and feeds with integrated and statistical approaches. Methods Mol Biol. 2014;1104:117-35. doi: 10.1007/978-1-62703-733-4_9.

13. Ghafuri-Esfahani A, Shokri R, Sharifi A, Shafiee L, Khosravi R, Kaghazian H, et al. Optimization of parameters affecting on CHO cell culture producing recombinant erythropoietin. Preparative Biochemistry & Biotechnology. 2020 Apr;50(8):834-41. doi: 10.1080/10826068.2020.1753072.

14. Trummer E, Fauland K, Seidinger S, Schriebl K, Lattenmayer C, Kunert R, et al. Process parameter shifting: part I. Effect of DOT, pH, and temperature on the performance of Epo-Fc expressing CHO cells cultivated in controlled batch bioreactors. Biotechnology and Bioengineering. 2006 Aug;94(6):1033-44. doi: org/10.1002/bit.21013.

~~1.~~15. Liu CH, Chen LH. Promotion of recombinant macrophage colony stimulating factor production by dimethyl sulfoxide addition in Chinese hamster ovary cells. J Biosci Bioeng. 2007 Jan;103(1):45-9. doi: 10.1263/jbb.103.45.

~~2.~~16. Ha TK, Kim YG, Lee GM. Effect of lithium chloride on the production and sialylation of Fc-fusion protein in Chinese hamster ovary cell culture. Appl Microbiol Biotechnol. 2014;98:9239-48. doi: 10.1007/s00253-014-6012-0.

~~3.~~17. Jiang Z, Sharfstein ST. Sodium butyrate stimulates monoclonal antibody over-expression in CHO cells by improving gene accessibility. Biotechnol Bioeng. 2008 May 1;100(1):189-94. doi: 10.1002/bit.21726.

~~4.~~18. Park JH, Noh SM, Woo JR, Kim JW, Lee GM. Valeric acid induces cell cycle arrest at G1 phase in CHO cell cultures and improves recombinant antibody productivity. Biotechnol J. 2016 Mar;11(4):487-96. doi: 10.1002/biot.201500327.

~~5.~~19. Yang WC, Lu J, Nguyen NB, Zhang A, Healy NV, Kshirsagar R, et al. Addition of valproic acid to CHO cell fed-batch cultures improves monoclonal antibody titers. Mol Biotechnol. 2014 May;56(5):421-8. doi: 10.1007/s12033-013-9725-x.

~~6.~~20. Toronjo-Urquiza L, James DC, Nagy T, Falconer RJ. Screening naturally occurring phenolic antioxidants for their suitability as additives to CHO cell culture media used to produce monoclonal antibodies. Antioxidants (Basel). 2019 Jun 3;8(6):159. doi: 10.3390/antiox8060159.

~~7.~~21. Chen F, Fan L, Wang J, Zhou Y, Ye Z, Zhao L, et al. Insight into the roles of hypoxanthine and thymidine on cultivating antibody-producing CHO cells: cell growth, antibody production and long-term stability. Appl Microbiol Biotechnol. 2012 Jan;93(1):169-78. doi: 10.1007/s00253-011-3484-z.

~~8.~~22. Du Z, Treiber D, McCarter JD, Fomina-Yadlin D, Saleem RA, McCoy RE, et al. Use of a small molecule cell cycle inhibitor to control cell growth and improve specific productivity and product quality of recombinant proteins in CHO cell cultures. Biotechnol Bioeng. 2015 Jan;112(1):141-55. doi: 10.1002/bit.25332.

~~9.~~23. Baek E, Lee JS, Lee GM. Untangling the mechanism of 3-methyladenine in enhancing the specific productivity: transcriptome analysis of recombinant Chinese hamster ovary cells treated with 3-methyladenine. Biotechnol Bioeng. 2018 Sep;115(9):2243-54. doi: 10.1002/bit.26777.

~~10.~~24. Arden N, Ahn SH, Vaz W, Rhodes M, Hancock C, Abitorabi MA, et al. Chemical caspase inhibitors enhance cell culture viabilities and protein titer. Biotechnol Prog. 2007;23:506-11. doi: 10.1021/bp060222m.

~~11.~~25. Joshi SD, More Y, Vagdevi HM, Vaidya VP, Gadaginamath GS, Kulkarni VH~~, et al~~. Synthesis of new 4-(2,5-dimethylpyrrol-1-yl)/4-pyrrol-1-yl benzoic acid hydrazide analogs and some derived oxadiazole, triazole and pyrrole ring systems: a novel class of potential antibacterial, antifungal and antitubercular agents. Med Chem Res. 2013;22:1073-89. doi: 10.1007/s00044-012-0112-0.

~~12.~~26. Joshi SD, More UA, Dixit SR, Korat HH, Aminabhavi TM, Badiger AM~~, et al~~. Synthesis, characterization, biological activity, and 3D-QSAR studies on some novel class of pyrrole derivatives as antitubercular agents. Med Chem Res. 2014;23:1123-47. doi: 10.1007/s00044-013-0709-y.

~~13.~~27. Zhao T, Zhao Z, Lu F, Chang S, Zhang J, Pang J, et al. Two- and three-dimensional QSAR studies on hURAT1 inhibitors with flexible linkers: topomer CoMFA and HQSAR. Mol Divers. 2020;24:141-54. doi: 10.1007/s11030-019-09936-5.

~~14.~~28. Renard JM, Spagnoli R, Mazier C, Salles MF, Mandine E. Evidence that monoclonal antibody production kinetics is related to the integral of the viable cells curve in batch systems. Biotechnol Lett. 1988;10(2):91-6. d~~D~~oi:~~;~~ 10.1007/BF01024632.

~~15.~~29. Chen F, Ye Z, Zhao L, Liu X, Fan L, Tan WS. Correlation of antibody production rate with glucose and lactate metabolism in Chinese hamster ovary cells. Biotechnol Lett. 2012;34:425-32. doi~~.~~: 10.1007/s10529-011-0798-y.

30. Becker M, Junghans L, Teleki A, Bechmann J, Takors R. The less the better: how suppressed base addition boosts production of monoclonal antibodies with Chinese hamster ovary cells. Front Bioeng Biotechnol. 2019;7:76. doi: 10.3389/fbioe.2019.00076.

31. Hara KY, Kondo A. ATP regulation in bioproduction. Microb Cell Fact. 2015;14:198. doi: 10.1186/s12934-015-0390-6.

~~16.~~1. ~~Mulukutla BC, Khan S, Lange A, Hu WS. Glucose metabolism in mammalian cell culture: new insights for tweaking vintage pathways. Trends Biotechnol. 2010 Sep;28(9):476-84. doi: 10.1016/j.tibtech.2010.06.005~~

~~17.~~32. Lowenstein J. Methods in enzymology, volume 13: ~~C~~citric acid cycle. 1st ed. Boston: Academic Press; 1969.

~~18.~~33. Kay J, Weitzman PD. Krebs~~'~~ citric acid cycle: half a century and still turning. 1st ed. London: Biochemical Society; 1987.

19.1. Becker M, Junghans L, Teleki A, Bechmann J, Takors R. The less the better: how suppressed base addition boosts production of monoclonal antibodies with Chinese hamster ovary cells. Front Bioeng Biotechnol. 2019;7:76. doi: 10.3389/fbioe.2019.00076

20.1. Hara KY, Kondo A. ATP regulation in bioproduction. Microb Cell Fact. 2015;14:198. doi: 10.1186/s12934-015-0390-6

34. Mulukutla BC, Khan S, Lange A, Hu WS. Glucose metabolism in mammalian cell culture: new insights for tweaking vintage pathways. Trends Biotechnol. 2010 Sep;28(9):476-84. doi: 10.1016/j.tibtech.2010.06.005.

21.35. Hodoniczky J, Zheng YZ, James DC. Control of recombinant monoclonal antibody effector functions by Fc *N*-glycan remodeling in vitro. Biotechnol Prog. 2005;21:1644-52. doi: 10.1021/bp050228w.

22.36. Liu L. Antibody glycosylation and its impact on the pharmacokinetics and pharmacodynamics of monoclonal antibodies and Fc-fusion proteins. J Pharm Sci. 2015;104(6):1866-84. doi: 10.1002/jps.24444.

23.37. Simonov V, Ivanov S, Smolov M, Abbasova S, Piskunov A, Poteryaev D. Control of therapeutic IgG antibodies galactosylation during cultivation process and its impact on IgG1/FcγR interaction and ADCC activity. Biologicals. 2019 Mar;58:16-21. doi: 10.1016/j.biologicals.2019.01.002.

Formatted: Font: Italic

~~24.~~38. Thomann M, Reckermann K, Reusch D, Prasser J, Tejada ML. Fc-galactosylation modulates antibody-dependent cellular cytotoxicity of therapeutic antibodies. Mol Immunol. 2016 May;73:69-75. doi: 10.1016/j.molimm.2016.03.002.

~~25.~~39. Thomann M, Schlothauer T, Dashivets T, Malik S, Avenal C, Bulau P, et al. In vitro glycoengineering of IgG1 and its effect on Fc receptor binding and ADCC activity. PLoS One. 2015;10(8):e0134949. doi: 10.1371/journal.pone.0134949.

~~26.~~40. Gramer MJ, Eckblad JJ, Donahue R, Brown J, Shultz C, Vickerman K, et al. Modulation of antibody galactosylation through feeding of uridine, manganese chloride, and galactose. Biotechnol Bioeng. 2011 Jul;108(7):1591-602. doi: 10.1002/bit.23075.

~~27.~~41. Williamson J, Miller J, McLaughlin J, Combs R, Chu C. Scale-dependent manganese leaching from stainless steel impacts terminal galactosylation in monoclonal antibodies. Biotechnol Prog. 2018 Oct;34(5):1290-7. doi: 10.1002/btpr.2662.

~~28.~~42. Prabhu A, Gadre R, Gadgil M. Zinc supplementation decreases galactosylation of recombinant IgG in CHO cells. Appl Microbiol Biotechnol. 2018;102:5989-99. doi: 10.1007/s00253-018-9064-8.

~~29.~~43. Chen P, Harcum SW. Effects of elevated ammonium on glycosylation gene expression in CHO cells. Metab~~ol~~ Eng. 2006 Mar;8(2):123-32. doi: 10.1016/j.ymben.2005.10.002.

~~30.~~44. Chen P, Harcum SW. Effects of amino acid additions on ammonium stressed CHO cells. J Biotechnol. 2005 May 25;117(3):277-86. doi: 10.1016/j.jbiotec.2005.02.003.

Formatted: Font: (Default) Times New Roman

Formatted: Font: (Default) Times New Roman

Supporting I~~i~~nformation

S1 Fig. The fed-batch culture profile in the retained glucose condition. Glucose was added on days 8 and 12 to keep the concentration above 1 g/L in the fed-batch cultures. And Feed medium was added on days 4, 6, and 8. V~~CD~~iable cell density (**A**), ~~V~~viability (**B**), ~~G~~glucose concentration (**C**), and ~~L~~lactate concentration (**D**) were measured every 2 days.

~~**S2 Fig. MPPB affects intracellular ATP amounts in rCHO cells.** The tested MPPB concentration was 0.64 mM in the culture solution, and the ATP amounts were analyzed by sampling day-4 cells in the batch mode cell cultures.~~**S2 Fig. The trend of viability and cell-specific productivity in each pyrrole derivatives concentration.** Viability (A) and cell-specific productivity (B) were analyzed by sampling day-3 cells in the batch cultures. The tested pyrrole derivative concentrations were 0.08 to 0.64 mM in the culture solutions. Culture conditions were considered adequate when viability was above 80%.

S1 Table. The validity of the first screening condition. DMSO was used as the 0% control, and 4-phenyl butyric acid dissolved in DMSO was used as the 100% control. Validity was evaluated at the points of coefficient of variation (CV) using day 3 cell culture solution.

Yong-Bin Yan, PhD
Academic Editor
PLOS ONE

January 21, 2021

Dear Dr. Yan,

Thank you for inviting us to submit a revised draft of our manuscript, entitled "4-(2,5-Dimethyl-1*H*-pyrrol-1-yl)-*N*-(2,5-dioxopyrrolidin-1-yl) benzamide improves monoclonal antibody production in a Chinese hamster ovary cell culture" to *PLOS ONE*. We appreciate the insightful feedback from you and the reviewers, and we used this feedback to strengthen our manuscript. We hope the responses provided below satisfactorily address the comments and questions from you and the reviewers. To facilitate the review of our revisions, point-by-point responses to the questions and comments are included below.

Responses to the Editor:

Comment 1

Please ensure that your manuscript meets PLOS ONE's style requirements, including those for file naming.

Response

We revised the manuscript to meet *PLOS ONE*'s style requirements. For example, we updated our file names, as well as the font size and equation formatting in the manuscript document, which were noted by reviewer #1. Thank you.

Comment 2

We note that you have indicated that data from this study are available upon request. PLOS only allows data to be available upon request if there are legal or ethical restrictions on sharing data publicly.

Response

Thank you for your observation. The chemical compound library is legally restricted by contract with the Basis for Supporting Innovative Drug Discovery and Life Science Research (BINDS) program. Therefore, we would like to update the information regarding data availability according to your advice as follows.

"Data Availability Statement:
The chemical compound library used for screening in this study is owned by the Basis

for Supporting Innovative Drug Discovery and Life Science Research (BINDS) program. BINDS has contractually imposed upon us restrictions involving data availability. The contact information of the data access committee and other institutional bodies are as follows:

Data access committee
Basis for Supporting Innovative Drug Discovery and Life Science Research (BINDS)
1-1-1, Yayoi, Bunkyo-ku, Tokyo 113-8657, Japan
E-mail: assist@mail.ecc.u-tokyo.ac.jp
Website: https://www.binds.jp/

Other institutional bodies
1. University of Tokyo
7-3-1, Hongo, Bunkyo-ku, Tokyo 113-0033, Japan
E-mail: ddiinfo@mol.f.u-tokyo.ac.jp

2. Osaka University
1-6, Yamadaoka, Fukida-shi, Osaka 565-0871, Japan
E-mail: yakugaku-syomu@office.osaka-u.ac.jp

3. Tohoku University
6-3, Aoba, Aramaki-aza, Aoba-ku, Sendai-shi, Miyagi 980-8578, Japan
E-mail: www@mail.pharm.tohoku.ac.jp

There are no restrictions on the other data we studied. These data are included in the paper and Supporting Information files."

Comment 3 and 4-1

We answer funding statement for Comment 3 and a part of comment 4 in this same section.

I. Comment 3

We note that you have provided funding information that is not currently declared in your Funding Statement. However, funding information should not appear in the Acknowledgments section or other areas of your manuscript. We will only publish funding information present in the Funding Statement section of the online submission form. Please remove any funding-related text from the manuscript and let us know how

you would like to update your Funding Statement.

II. Comment 4-1

We note that one or more of the authors are employed by a commercial company: Daiichi Sankyo Co., Ltd.

1. Please provide an amended Funding Statement declaring this commercial affiliation, as well as a statement regarding the Role of Funders in your study. If the funding organization did not play a role in the study design, data collection and analysis, decision to publish, or preparation of the manuscript and only provided financial support in the form of authors' salaries and/or research materials, please review your statements relating to the author contributions, and ensure you have specifically and accurately indicated the role(s) that these authors had in your study. You can update author roles in the Author Contributions section of the online submission form.

Response

Thank you for allowing us to amend this information. We removed appreciation for BINDS from the Acknowledgments section of the manuscript based on your comment. Instead, we named two individuals who supported the study. In addition, Daiichi Sankyo Co., Ltd., did not play a role in the study design or any other aspect. Further, the specific roles of the authors were revised in the Author Contributions section. We would like to revise our funding information as follows.

"Funding Statement:
1. The chemical compound library used in this study was provided by the Platform Project for Supporting Drug Discovery and Life Science Research (the Basis for Supporting Innovative Drug Discovery and Life Science Research [BINDS] program) from the Japan Agency for Medical Research and Development (AMED) under grant numbers JP19am0101086, JP19am0101084, and JP19am0101095 (support numbers 1009, 0847, and 1011, respectively). The funders had no role in the study design, data collection, data analysis, decision to publish, or preparation of the manuscript.
2. Daiichi Sankyo Co., Ltd., provided support in the form of salaries for the authors and supporters (YA, YK, HK, KN, AK, and MK), but the company did not have any additional role in the study design, data collection, data analysis, decision to publish, or preparation of the manuscript. The specific roles of these authors are articulated in the Author Contributions section."

Comment 4-2

We note that one or more of the authors are employed by a commercial company: Daiichi Sankyo Co., Ltd.
Please also provide an updated Competing Interests Statement declaring this commercial affiliation along with any other relevant declarations relating to employment, consultancy, patents, products in development, or marketed products, etc.
<u>Response</u>
Thank you for allowing us to amend this statement. Based on the journal's requirements, we have revised the statement as follows.

"Competing Interests Statement:
Daiichi Sankyo Co., Ltd. employs some authors [YA, YK, HK, and KN]. This commercial affiliation does not alter your adherence to all PLOS ONE policies on sharing data and materials by including the following statement: "This does not alter our adherence to PLOS ONE policies on sharing data and materials."

Responses to Reviewer #1:
Thank you for your advice, which was valuable as we revised our manuscript. We appreciate your suggestions regarding grammar and scientific wording. The revised document "PONE-D-20-26236_reviewer V1_authors V1" was submitted in addition to this manuscript.

<u>Specific Comment 1</u>
Word-choice: due to robustness and high productivity/ability to produce proteins with ideal glycans.
<u>Response</u> (See page 2, lines 23-24 of the revised manuscript)
Thank you for this comment. The sentence was revised as follows.

"due to robustness, high productivity and ability to produce proteins with ideal glycans."

<u>Specific Comment 2</u>
Add examples of chemical compounds. "Chemical compounds such as... have been shown to improve"
<u>Response</u> (See page 2, lines 24-25 of the revised manuscript)
Thank you for this comment. The sentence was revised as follows.

"Some chemical compounds such as dimethyl sulfoxide, lithium chloride and butyric

acid, and so on, have been shown to improve"

<u>Specific Comment 3</u>

Revise “from compounds to improve” to “compounds that improve”

<u>Response</u> (See page 2, lines 26 of the revised manuscript)

Thank you for this comment. The sentence was revised as “compounds that can improve”.

<u>Specific Comment 4</u>

Need revision of grammar, and addition of medicine examples and references. “Monoclonal antibody (mAb) treatment is becoming a common therapeutic treatment for cancer and autoimmune disorders.”

<u>Response</u> (See page 3, lines 40-42 of the revised manuscript)

We revised the sentence by adding medicine examples and references as follows.

“The use of therapeutic monoclonal antibodies (mAb) is becoming more common in the treatment of cancer (with drugs such as pembrolizumab, nivolumab, trastuzumab, and so on) and autoimmune diseases (with drugs such as adalimumab, infliximab, and so on) [1-7].”

<u>Specific Comment 5</u>

Revise following grammars; “Several grams/liter of production by recombinant Chinese hamster ovary (rCHO) cells, which harbor mAb genes”

<u>Response</u> (See page 3, lines 44-45 of the revised manuscript)

We revised the sentence as follows.

"MAb concentrations of several grams per liter, produced by using recombinant Chinese hamster ovary (rCHO) cells, which harbor genes encoding for mAb"

<u>Specific Comment 6</u>

What does mean the “state”?

<u>Response</u> (See page 3, lines 54 of the revised manuscript)

We thought the cell cycle explained “the state.” We revised this to “intracellular state.”

Specific Comment 7

How was structure-activity relationship evaluated? "The structure-activity relationship of MPPB was also evaluated." is abstract.

Response (See page 4, lines 66-69 of the revised manuscript)

We agree that this sentence was abstract and should include a note on 2,5-dimethylpyrrole. The sentence was revised as follows.

"The structure-activity relationship of MPPB was also evaluated using the compounds corresponding to the partial structures of MPPB as additives in batch cultures and demonstrated that 2,5-dimethylpyrrole exhibited a strong enhancement of the cell-specific productivity of mAb."

Specific Comment 8

Does "a target concentration" mean "a target viable cell concentration"?

Response (See page 5, lines 85 and 92, page 6, lines 106, and page 7, lines 115 of the revised manuscript)

Yes, it means "a target viable cell concentration." We revised all relevant sentences to use this alternate terminology.

Specific Comment 9

Working volume of 50 mL in 125 mL or 50 mL of media and then seed culture? Which is correct?

Response (See page 5, lines 86-87, page 6, lines 108 and page 7 line 116 of the revised manuscript)

A working volume of 50 mL is correct. The related sentences were revised as follows.

"The working volume was adjusted to 50 mL in the 125-mL flasks and to 100 mL in the 250-mL flasks." (Lines 86-87)

"The working volume was adjusted to 10 mL." (Line 108)

"The working volume was adjusted to 50 mL." (Line 116)

Specific Comment 10

Need more context. Is it (arbitrary unit) mab concentration or not?

Response (See page 6, lines 98-99 of the revised manuscript)

It was not the mAb concentration. This indicator was the arbitrary unit, which was converted from the mAb concentration. The following context was added to the manuscript.

"The resulting mAb concentrations were converted to arbitrary units according to the following equation (1)"

Specific Comment 11

Use format as equation 1 or list as text (- is minus or subtract from

Response (See page 6, lines 100-101 of the revised manuscript)

Based on the reviewer's comment, we formatted equation (1) as follows.

$$\text{Arbitrary unit} = (\text{tested mAb concentration} - (\text{average mAb concentration of the controls} + 3 \text{ standard deviations}))$$

Specific Comment 12

There was substantial grammatical issues, primarily in the abstract and introduction which will require a major revision. One particular issue in the results section is the use of glucose consumption and lactate accumulation. This terminology should be clarified to be either glucose concentration, cell-specific glucose uptake rate, or cumulative glucose consumption, where applicable.

Response (See lines 30, 65, 129, 253, 254, 256, 259, 265, 266, 287, 293, 295, 364, and 358-359; Figs 6, 8, and 10; and S1 Fig of the revised manuscript)

Based on the reviewer's comment, we distinguished between "glucose concentration" and "cell-specific glucose uptake rate" and between "lactate concentration" and "cell-specific lactate production rate" in the relevant areas of the manuscript.

Specific Comment 13

Were they (5 compounds) replaced (with 67 compounds)? That would be a total of 67 compounds.

Response (See page 10, lines 176-178 of the revised manuscript)

Although 67 chemical compounds were commercially available, another 5 compounds for which the original structures were not commercially available were also tested as derivatives. Therefore, the total number of compounds was 72. Thank you very

much for your suggestion about grammar. We revised the text as follows.

"Although 67 chemical compounds were commercially available, another 5 compounds for which the original structures were not commercially available were also tested as derivatives. Therefore, a total of 72 samples underwent the second screening in suspension culture."

Specific Comment 14

This sentence is repetitive. Write a better transition.

"Relative cell-specific productivity, relative mAb concentration, and viability were compared with the control condition. For most of the compounds, the relative mAb concentration was increased with the chemical compound treatment. Therefore, the chemical compounds were screened for relative mAb concentration, relative cell-specific productivity, and viability."

Response (See page 10, lines 186-187 of the revised manuscript)

We revised the sentence as follows.

"The relative cell-specific productivity, relative mAb concentration, and viability were assessed to evaluate the chemical compound treatments against the control condition."

Specific Comment 15

Need revise the sentence like following;

"As ID number 62 demonstrated the largest effect as so was structure-activity relationship investigated further"

Response (See page 11, lines 201-202 of the revised manuscript)

Thank you very much for your suggestion for clarifying the sentence. Other factors were also tested in batch and fed-batch cultures. We therefore revised the sentence as follows.

"As ID number 62 demonstrated the largest effect, we further investigated the characteristics of ID number 62 in batch and fed-batch cultures."

Specific Comment 16

Lactate is also double.

Response (See pages 14, lines 255-267 of the revised manuscript)

As you pointed out, lactate was also doubled. However, we would like to

mention that the increase in the cell-specific lactate production rate was smaller than the increase in the cell-specific glucose uptake rate. This finding means that glucose was efficiently used in the tricarboxylic acid cycle. We revised the text as follows.

"In other words, the cell-specific glucose uptake rate under the MPPB-added condition was 0.61 pmol/cell/day higher than that under the control condition. Meanwhile, the cell-specific lactate production rate increased from 0.22 to 0.37 pmol/cell/day under the MPPB-added condition (Fig 8D). The increase in the cell-specific lactate production rate was 0.15 pmol/cell/day, which was smaller than the increase in the cell-specific glucose uptake rate (0.61 pmol/cell/day). This result suggested that consumed glucose was efficiently converted to ATP in the tricarboxylic acid cycle."

Specific Comment 17

Combine with first sentence.

"The glucose consumption rate under the control condition was faster than that under the MPPB-added condition, as shown in Fig 9A. Residual glucose was fully consumed by day 10. On the other hand, glucose under the MPPB-added condition was retained until day 14. The lactate concentration was kept under 0.8 g/L, and no difference was noted in both conditions (Fig 9B). The specific glucose consumption rates were 0.63 and 0.74 pmol/cell/day under the control and MPPB-added conditions, respectively (Fig 9C)."

Response (See page 16, lines 286-290 of the revised manuscript)

Thank you for this suggestion. We revised the text as follows.

"Residual glucose was fully consumed by day 10 under the control condition, while glucose under the MPPB-added condition was retained until day 14 (Fig 10A). The cell-specific glucose uptake rates were 0.63 and 0.74 pmol/cell/day under the control and MPPB-added conditions, respectively (Fig 10C). The lactate concentration was kept under 0.8 g/L and no difference was noted in both condition (Fig 10B)."

Specific Comment 18

"Cell-specific glucose uptake rate (C) was indicated as a slope of consumed glucose concentration against integral viable cell concentration [28, 29]." This sentence should be noted earlier.

Response (See page 8-9, lines 149-154 and page 14, lines 250-251 of the revised manuscript)

Based on the reviewer's comment, we added the calculation method to the Materials and Methods section. Similar calculation methods were used for cell-specific productivity and cell-specific lactate production rate. In addition, the written expressions in the manuscript were aligned. We updated the manuscript as follows.

"**Calculation of cell-specific productivity, cell-specific glucose uptake rates, and cell-specific lactate production rates**

Cell-specific productivity (pg/cell/day), cell-specific glucose uptake rates (pmol/cell/day), and cell-specific lactate production rates (pmol/cell/day) were calculated as the slopes of mAb concentration (mg/L), consumed glucose concentration (mM), and lactate concentration (mM), respectively, to integral viable cell concentration ((cells·day)/mL), according to previous reports [28, 29]." (Lines 151-154)

"The slopes of mAb concentration (**C**) to integral viable cell concentration was used to indicate cell-specific productivity (**D**) [28]." (Lines 250-251)

Specific Comment 19

You've demonstrated a chemical dose-dependent response to viability. Should mention that lower concentrations would need to be tested to understand potency each of the chemicals 2,5-dimethylpyrrole is most comparable to MPPB.

Response (See page 18, lines 331-336 of the revised manuscript)

Thank you for this important comment. We added new data about the relationships among some pyrroles' concentrations, cell-specific productivity, and viability. The data showed that 2,5-dimethylpyrrole was the best, with viability above 80%, which is a realistic condition for cell cultures. In addition, 3,4-dimethylpyrrole had high cell-specific productivity. However, it had lower productivity than 2,5-dimethylpyrrole. We think the data support the conclusion that 2,5-dimethylpyrrole was the best compound. Therefore, the data were added to the Supporting Information (S2 Fig). Moreover, the following context was added to the manuscript.

"Thus, 2,5-dimethylpyrrole (13) was found to be the most effective chemical structure to increase cell-specific productivity while retaining viability in the 0.32 mM concentration. In addition, alkyl-pyrroles (9-14) that showed high cell-specific productivity in the 0.32 mM concentration were tested to clarify which pyrrole derivative was the best compound in terms of increasing cell-specific productivity and

retaining viability. The results showed that 2,5-dimethylpyrrole (13) effectively increased cell-specific productivity while retaining viability (S2 Fig)."

<u>Specific Comment 20</u>

"Viability" would be correct, but not "extension of cell culture period".

<u>Response</u> (See page 4, line 64 and page 21, lines 350 and 353-354 of the revised manuscript)

Thank you very much for this clarification. Retained viability contributed to extended cell culture periods. Therefore, "viability" is appropriate in this context. In all related sentences, we changed language reflecting "extension of cell culture period" to language reflecting "viability."

<u>Specific Comment 21</u>

How much lower? Please clarify the value.

<u>Response</u> (See page 21, lines 357-358 of the revised manuscript)

Based on the reviewer's comment, we clarified the value as follows.

"…viability under the control condition was 45% lower than that under the MPPB-added condition on day 12."

<u>Specific Comment 22</u>

Unlikely... lower growth indicates cell cycle arrest. Cells consume less amino acids where growth is arrested. Higher cell density and increased growth would deplete component most likely.

<u>Response</u> (See page 21, lines 358-360 of the revised manuscript)

Based on the reviewer's comment, we revised the sentence as follows.

"These results suggested that cell growth suppression by MPPB treatment prevented the depletion of medium components except for glucose, resulting in maintained viability."

<u>Specific Comment 23</u>

This is also unlikely. "The cause of increased cell-specific productivity, however, is clearer. In our study, MPPB treatment had two important effects to increase cell-specific productivity. One was the suppression of cell growth (Figs 6A and 8A), and the other was the acceleration of specific glucose consumption. Both the effects would control the amount of intracellular adenosine triphosphates (ATPs)"

Response (See pages 21, lines 361-380 and Fig 6 of the revised manuscript)

We understand your concern. We included a new figure (Fig 6), mentioned in the Results section, that shows the relationship between the amount of intracellular ATP, the cell-specific glucose uptake rate, cell-specific productivity, and viable cell density (VCD). The figure shows that intracellular ATP amounts, cell-specific glucose uptake rates, and cell-specific productivity were completely positively correlated. On the other hand, intracellular ATP amounts and VCD had a negative correlation. These phenomena have been reported by Becker et al. and Hara and Kondo, who found that intracellular ATP amounts were positively correlated with cell-specific productivity (refs. 30 and 31 of the revised manuscript). Further, increased cell-specific glucose uptake rate and suppressed VCD have been shown to increase the amount of intracellular ATP (refs. 32-34 of the revised manuscript). Therefore, we think that one of the reasons for the increased productivity was the enhanced amount of intracellular ATP.

Specific Comment 24

Glucose is converted to pyruvate. Pyruvate is either converted to lactate or enters the TCA cycle.

Response (See pages 22, lines 366-368 and Fig 6 of the revised manuscript)

We added the following explanation.

"An increased cell-specific glucose uptake rate leads to an increased amounts of intracellular ATP when glucose is metabolized to ATP in the tricarboxylic acid cycle [32, 33], but not when glucose is metabolized to lactate."

Our results showed that the cell-specific lactate production rate increase was lower than the cell-specific glucose uptake rate increase. (This result was also added to the revised manuscript [lines 253-261]). This finding means that most consumed glucose was changed to ATP. To support this explanation, the ATP amounts in the batch cultures were added as new data (Fig 6).

Specific Comment 25

Should be in the results section. Where is the method for this? (about ATP)

Response (See pages 8, lines 138-147; page 13, lines 227-235; page 22, lines 371-378; and Fig 6 of the revised manuscript)

We described the ATP analysis method in the Materials and Methods section

(pages 8, lines 138-147). In addition, the content of Fig 6 is now reflected in the Results section (page 13, lines 227-235). Finally, the content of the Discussion section was revised (page 22, lines 371-378). The specific updates are as follows.

Materials and Methods section:

“**Measuring intracellular ATP**

An intracellular ATP assay kit (v2, Toyo B-Net, Tokyo, Japan) was used to quantitate ATP amounts in rCHO cells. Each cell culture solution (100 µL) was diluted with phosphate-buffered saline (1 mL) and then centrifuged (450 g, 5 min), and the supernatant was discarded (1 mL). The resulting cell pellet was suspended in phosphate-buffered saline (1 mL) and then centrifuged (450 g, 5 min), and the supernatant was again discarded (1 mL). The cell pellet was suspended in the ATP extraction reagent from the kit (0.5 mL) and incubated for 5 minutes to extract ATP from the cells. The suspension (10 µL) was mixed with luciferase luminescence assay reagents (100 µL), and measurements were obtained by using the Infinite M Plex plate reader (Tecan, Mannedorf, Switzerland). The resulting ATP concentration was converted to the amount of intracellular ATP per cell.”

Results section:

“Becker et al. [30] and Hara and Kondo [31] reported that increased amounts of intracellular ATP are associated with increased cell-specific productivity. Therefore, we investigated intracellular ATP amounts and cell-specific glucose uptake rates, which are related to ATP production [32, 33], in the batch cultures to clarify the reason for the increasing cell-specific productivity (Fig 6). The results showed that both intracellular ATP amounts and cell-specific glucose uptake rates were increased with higher MPPB concentrations. Intracellular ATP amounts increased from 8 to 24 fmol/cell (Fig 6A), and cell-specific glucose uptake rates increased from 0.9 to 2.6 pmol/cell/day (Fig 6B). Intracellular ATP amounts and cell-specific glucose uptake rates were positively correlated with cell-specific productivity (Fig 6C). Additionally, VCD was suppressed with MPPB treatment (Fig 6D)”

Discussion section:

“To test this hypothesis, we investigated the relationship between the amount of intracellular ATP and MPPB concentration (Fig 6). We found that the amount of intracellular ATP was enhanced with higher MPPB concentrations (Fig 6A).

Cell-specific glucose uptake rates and cell-specific productivity were also increased (Figs 6B and 6C). On the other hand, VCD was also suppressed with higher MPPB conditions (Fig 6D). Therefore, we predicted that the increased cell-specific productivity under the MPPB-added condition might occur due to the increasing amount of intracellular ATP resulting from cell growth suppression and enhanced cell-specific glucose uptake rates."

Specific Comment 26

How is MPPB structure similar to CDK4/6 inhibitor? What other hypothesis do you have for galactosylation suppression?

Response

Thank you for this question. MPPB and CDK4/6 are both alkaloids. However, their chemical structures are completely different. MPPB consists of succinimide, amino benzamide, and 2,5-dimethylpyrrole, while CDK4/6 consists of pyrimidine, 2-amino pyridine, piperazine, etc. We do not yet have any hypothesis regarding the galactosylation suppression. Therefore, we need to further study the mechanism for galactosylation suppression.

Specific Comment 27

Substantially and why is this? (The viability was decreased in the cases of other mono- or bis-alkyl-substituted pyrroles.)

Response

Although we considered the reason from the point of view of the electron density distribution of the chemical structure, the reason has not yet been clarified. As you mentioned, adequate concentrations exist for each compound (see S2 Fig of the revised manuscript). However, we cannot yet answer how the alkyl-substituted position affects viability. This mechanism requires further study.

Specific Comment 28

The discovered chemical additives should be compared to known additives that boost specific productivity as part of the structure-activity section.

Response

Thank you very much for this important suggestion. We also think that we should further the study in this way. We are proceeding with further study of MPPB and 2,5-dimethylpyrrole with comparisons to known additives. Therefore, in the structure-activity section of the present article, we would like to report only the

chemical structure of MPPB and the structure-activity relationship of the most effective structure. We still believe the current content will be worthwhile for readers.

Responses to Reviewer #2:

Thank you very much for your essential questions, which we addressed to strengthen our study. The questions helped us to improve our manuscript and to focus the direction of our future research.

Specific Comment 1

Why does MPPB suppress cell growth and increase specific glucose consumption during monoclonal antibody production? Also, it also can suppress the galactosylation on a monoclonal antibody. Could you please explain why?

Response

This is a key question. We analyzed RNA sequences in a preliminary study, but differences between the control and MPPB-added conditions were not investigated. Therefore, we cannot provide an explanation based on the current data. We would like to clarify this characteristic as much as possible in future research.

Specific Comment 2

Could you please explain the concentration of MPPB on cells growth and monoclonal antibody production?

Response (See page 12, lines 217-221 and Fig 5D of the revised manuscript)

Thank you for pointing out the relationship between MPPB concentration and mAb production. MAb production decreased with higher MPPB concentrations due to cell growth suppression (see Fig 5D, which was newly added to the revised manuscript). We paid attention to increased cell-specific production and proceeded with studies in batch and fed-batch cultures. The following content was added to the revised manuscript based on your question.

“MAb concentration decreased with the addition of MPPB due to suppressed cell growth in these short-period batch cultures (Fig 5D). These results showed that treatment with MPPB concentrations of 0.32 to 0.64 mM suppressed cell growth and increased cell-specific productivity while retaining viability. Therefore, MPPB concentrations of 0.32 to 0.64 mM were used to characterize MPPB.”

Specific Comment 3

What is the difference of MPPB from any other known chemicals for

monoclonal antibody production in Chinese hamster ovary cells?

Response (See page 22, lines 378-380 and 387 of the revised manuscript)

This compound would be the same as other compounds in terms of cell growth suppression and increased cell-specific productivity or mAb production. However, in most cases, the relationship between increased glucose consumption and increased intracellular ATP amounts is not reported or discussed (refs. 15-24 of the revised manuscript). This point will be new knowledge. Therefore, we added this information to the manuscript as follows.

"Our results showed that a chemical compound such as MPPB, which suppresses cell growth and increases intracellular ATP amounts, may improve mAb production in rCHO cell cultures."

Further, valeric acid, valproic acid, and CDK4/6 inhibitor are known to increase the galactosylation ratio (refs. 18, 19 and 22 of the revised manuscript), an outcome that differs from that of MPPB. Therefore, MPPB would also have worth from the point of diversity as a galactosylation control reagent. Valeric acid and valproic acid are now mentioned in the revised manuscript to strengthen the difference between MPPB and other chemical compounds.

Specific Comment 4

The authors claim MPPB increases the production of ATP, which is responsible for the increase of monoclonal antibody productivity? The reviewer wonders whether ATP has the same effect on the monoclonal antibody productivity? Why?

Response (See page 13, lines 227-235 and Fig 6 of the revised manuscript)

CHO cells need ATP for mAb production. In addition, there are some reports about the relationship between mAb production and the amount of intracellular ATP (refs. 30 and 31 of the revised manuscript). To support this theory, we added new information to the revised manuscript. The data showed that mAb production per cell and intracellular ATP amounts are completely correlated (see Fig 6 of the revised manuscript).

Specific Comment 5

How does MPPB regulate the produce of ATP in the cells?

Response (See page 13, lines 227-235; page 22, lines 361-380; and Fig 6 of the revised manuscript)

The detailed mechanism is not yet clear. However, based on our results and past reports, we suspect that cell growth suppression and increased cell-specific glucose consumption without much lactate accumulation are critical reasons. The former saves ATP (ref. 34 of the revised manuscript), and the latter produces ATP (refs. 32 and 33 of the revised manuscript). We found that the amount of intracellular ATP changed depending on MPPB concentration (Fig 6). This content was added to the manuscript. We need further studies to investigate the mechanism.

Specific Comment 6

The authors suggest 2,5-dimethylpyrrole was the most effective partial structure of MPPB on monoclonal antibody production. Whether can 2,5-dimethylpyrrole affect monoclonal antibody production through affecting the production of ATP? Is this mechanism from other known chemicals used for monoclonal antibody production in the cells?

Response

Thank you for your comment. This point is very important. Our research showed that 2,5-dimethylpyrrole increased intracellular ATP amounts while increasing cell-specific glucose uptake rates. This research is ongoing. Therefore, in this manuscript, we would like to mention only that 2,5-dimethylpyrrole was the most effective partial structure of MPPB. In the near future, we would like to report this additional information.

Other notes

We adjusted some words for easier understanding by readers. We apologize for these revisions.

(See page 20, line 338 of the revised manuscript)
Specific productivity → Cell-specific productivity

(See page 20, Table 1 of the revised manuscript)
Relative specific production rate → Relative cell-specific productivity

(See page 21, lines 395, 396, and 402 of the revised manuscript)
2,5-dimethyl pyrrole → 2,5-dimethylpyrrole

(See page 21, lines 395 of the revised manuscript)

2,5-dimethyl pyrrolidine →2,5-dimethylpyrrolidine

Again, thank you for giving us the opportunity to strengthen our manuscript by addressing your comments and questions. We incorporated your feedback. We appreciate your time and look forward to your response.

Sincerely,

Yuichi Aki
Biologics Technology Research Laboratories, Biologics Division, Daiichi Sankyo Co., Ltd.
2716-1, Aza Kurakake, Oaza Akaiwa, Chiyoda-machi, Oura-gun, Gunma 370-0503, Japan
E-mail: aki.yuichi.ha@daiichisankyo.co.jp
