# Supplementary material for: 4-(2,5-Dimethyl-1H-pyrrol-1-yl)-N-(2,5-dioxopyrrolidin-1-yl) benzamide improves monoclonal antibody production in a Chinese hamster ovary cell culture
Source: PLoS One. 2021 Apr 22;16(4):e0250416. doi: 10.1371/journal.pone.0250416 (PMC8061942; doi:10.1371/journal.pone.0250416)
Supplement: Attachment — Submitted filename: comments to PLoS ONE.docx [file pone.0250416.s005.docx]

It is significant to improve monoclonal antibody production using recombinant Chinese hamster ovary cells for medication supply and medical cost reduction. In the manuscript entitled“4-(2,5-Dimethyl-1H-pyrrol-1-yl)-N-(2,5-dioxopyrrolidin-1-yl) benzamide (MPPB) improves monoclonal antibody production in a Chinese hamster ovary cell culture”, the authors Yuichi Aki et al. have discovered a kind of new chemical MPPB to improve monoclonal antibody production effectively in recombinant Chinese hamster ovary cells through scale screening. They found that it can suppress cell growth and increase specific glucose consumption during monoclonal antibody production. In addition, it also can suppress the galactosylation on a monoclonal antibody. Further, the structure-activity study revealed that 2,5-dimethylpyrrole was the most effective partial structure of MPPB on monoclonal antibody production. The premise of this paper is compelling and the approach seems reasonable. While the results seem to contribute meaningfully to monoclonal antibody production in recombinant Chinese hamster ovary cells, several aspects of this paper - especially a more complete description and discussion of the advantage and disadvantages of this new chemical - could be improved including:

1. Why does MPPB suppress cell growth and increase specific glucose consumption during monoclonal antibody production? Also, it also can suppress the galactosylation on a monoclonal antibody. Could you please explain why?
2. Could you please explain the concentration of MPPB on cells growth and monoclonal antibody production?
3. What is the difference of MPPB from any other known chemicals for monoclonal antibody production in Chinese hamster ovary cells?
4. The authors claim MPPB increases the production of ATP, which is responsible for the increase of monoclonal antibody productivity? The reviewer wonders whether ATP has the same effect on the monoclonal antibody productivity? Why?
5. How does MPPB regulate the produce of ATP in the cells?
6. The authors suggest 2,5-dimethylpyrrole was the most effective partial structure of MPPB on monoclonal antibody production. Whether can 2,5-dimethylpyrrole affect monoclonal antibody production through affecting the production of ATP? Is this mechanism from other known chemicals used for monoclonal antibody production in the cells?
