# Supplementary material for: 4-(2,5-Dimethyl-1H-pyrrol-1-yl)-N-(2,5-dioxopyrrolidin-1-yl) benzamide improves monoclonal antibody production in a Chinese hamster ovary cell culture
Source: PLoS One. 2021 Apr 22;16(4):e0250416. doi: 10.1371/journal.pone.0250416 (PMC8061942; doi:10.1371/journal.pone.0250416)
Supplement: Attachment — Submitted filename: PONE-D-20-26236R1 Response to reviewers.doc [file pone.0250416.s006.doc]

Yong-Bin Yan, PhD

Academic Editor

*PLOS ONE*

January 21, 2021

Dear Dr. Yan,

Thank you for inviting us to submit a revised draft of our manuscript, entitled “4-(2,5-Dimethyl-1*H*-pyrrol-1-yl)-*N*-(2,5-dioxopyrrolidin-1-yl) benzamide improves monoclonal antibody production in a Chinese hamster ovary cell culture” to *PLOS ONE*. We appreciate the insightful feedback from you and the reviewers, and we used this feedback to strengthen our manuscript. We hope the responses provided below satisfactorily address the comments and questions from you and the reviewers. To facilitate the review of our revisions, point-by-point responses to the questions and comments are included below.

**Responses to the Editor:**

**Comment 1**

Please ensure that your manuscript meets PLOS ONE's style requirements, including those for file naming.

**Response**

We revised the manuscript to meet *PLOS ONE*’s style requirements. For example, we updated our file names, as well as the font size and equation formatting in the manuscript document, which were noted by reviewer #1. Thank you.

**Comment 2**

We note that you have indicated that data from this study are available upon request. PLOS only allows data to be available upon request if there are legal or ethical restrictions on sharing data publicly.

**Response**

Thank you for your observation. The chemical compound library is legally restricted by contract with the Basis for Supporting Innovative Drug Discovery and Life Science Research (BINDS) program. Therefore, we would like to update the information regarding data availability according to your advice as follows.

“Data Availability Statement:

The chemical compound library used for screening in this study is owned by the Basis for Supporting Innovative Drug Discovery and Life Science Research (BINDS) program. BINDS has contractually imposed upon us restrictions involving data availability. The contact information of the data access committee and other institutional bodies are as follows:

Data access committee

Basis for Supporting Innovative Drug Discovery and Life Science Research (BINDS)

1-1-1, Yayoi, Bunkyo-ku, Tokyo 113-8657, Japan

E-mail: [assist@mail.ecc.u-tokyo.ac.jp](mailto:assist@mail.ecc.u-tokyo.ac.jp)

Website: <https://www.binds.jp/>

Other institutional bodies

1. University of Tokyo

7-3-1, Hongo, Bunkyo-ku, Tokyo 113-0033, Japan

E-mail: [ddiinfo@mol.f.u-tokyo.ac.jp](mailto:ddiinfo@mol.f.u-tokyo.ac.jp)

2. Osaka University

1-6, Yamadaoka, Fukida-shi, Osaka 565-0871, Japan

E-mail: [yakugaku-syomu@office.osaka-u.ac.jp](mailto:yakugaku-syomu@office.osaka-u.ac.jp)

3. Tohoku University

6-3, Aoba, Aramaki-aza, Aoba-ku, Sendai-shi, Miyagi 980-8578, Japan

E-mail: [www@mail.pharm.tohoku.ac.jp](mailto:www@mail.pharm.tohoku.ac.jp)

There are no restrictions on the other data we studied. These data are included in the paper and Supporting Information files.”

**Comment 3 and 4-1**

We answer funding statement for Comment 3 and a part of comment 4 in this same section.

I. **Comment 3**

We note that you have provided funding information that is not currently declared in your Funding Statement. However, funding information should not appear in the Acknowledgments section or other areas of your manuscript. We will only publish funding information present in the Funding Statement section of the online submission form. Please remove any funding-related text from the manuscript and let us know how you would like to update your Funding Statement.

II. **Comment 4-1**

We note that one or more of the authors are employed by a commercial company: Daiichi Sankyo Co., Ltd.

1.	Please provide an amended Funding Statement declaring this commercial affiliation, as well as a statement regarding the Role of Funders in your study. If the funding organization did not play a role in the study design, data collection and analysis, decision to publish, or preparation of the manuscript and only provided financial support in the form of authors' salaries and/or research materials, please review your statements relating to the author contributions, and ensure you have specifically and accurately indicated the role(s) that these authors had in your study. You can update author roles in the Author Contributions section of the online submission form.

**Response**

Thank you for allowing us to amend this information. We removed appreciation for BINDS from the Acknowledgments section of the manuscript based on your comment. Instead, we named two individuals who supported the study. In addition, Daiichi Sankyo Co., Ltd., did not play a role in the study design or any other aspect. Further, the specific roles of the authors were revised in the Author Contributions section. We would like to revise our funding information as follows.

“Funding Statement:

1. The chemical compound library used in this study was provided by the Platform Project for Supporting Drug Discovery and Life Science Research (the Basis for Supporting Innovative Drug Discovery and Life Science Research [BINDS] program) from the Japan Agency for Medical Research and Development (AMED) under grant numbers JP19am0101086, JP19am0101084, and JP19am0101095 (support numbers 1009, 0847, and 1011, respectively). The funders had no role in the study design, data collection, data analysis, decision to publish, or preparation of the manuscript.

2. Daiichi Sankyo Co., Ltd., provided support in the form of salaries for the authors and supporters (YA, YK, HK, KN, AK, and MK), but the company did not have any additional role in the study design, data collection, data analysis, decision to publish, or preparation of the manuscript. The specific roles of these authors are articulated in the Author Contributions section.”

**Comment 4-2**

We note that one or more of the authors are employed by a commercial company: Daiichi Sankyo Co., Ltd.

Please also provide an updated Competing Interests Statement declaring this commercial affiliation along with any other relevant declarations relating to employment, consultancy, patents, products in development, or marketed products, etc.

**Response**

Thank you for allowing us to amend this statement. Based on the journal’s requirements, we have revised the statement as follows.

“Competing Interests Statement:

Daiichi Sankyo Co., Ltd. employs some authors [YA, YK, HK, and KN]. This commercial affiliation does not alter your adherence to all PLOS ONE policies on sharing data and materials by including the following statement: “This does not alter our adherence to　PLOS ONE policies on sharing data and materials.”

**Responses to Reviewer #1:**

Thank you for your advice, which was valuable as we revised our manuscript. We appreciate your suggestions regarding grammar and scientific wording. The revised document “PONE-D-20-26236_reviewer V1_authors V1” was submitted in addition to this manuscript.

**Specific Comment 1**

Word-choice: due to robustness and high productivity/ability to produce proteins with ideal glycans.

**Response** (See page 2, lines 23-24 of the revised manuscript)

Thank you for this comment. The sentence was revised as follows.

"due to robustness, high productivity and ability to produce proteins with ideal glycans."

**Specific Comment 2**

Add examples of chemical compounds. “Chemical compounds such as... have been shown to improve”

**Response** (See page 2, lines 24-25 of the revised manuscript)

Thank you for this comment. The sentence was revised as follows.

"Some chemical compounds such as dimethyl sulfoxide, lithium chloride and butyric acid, and so on, have been shown to improve"

**Specific Comment 3**

Revise “from compounds to improve” to “compounds that improve”

**Response** (See page 2, lines 26 of the revised manuscript)

Thank you for this comment. The sentence was revised as “compounds that can improve”.

**Specific Comment 4**

Need revision of grammar, and addition of medicine examples and references.

“Monoclonal antibody (mAb) treatment is becoming a common therapeutic treatment for cancer and autoimmune disorders.”

**Response** (See page 3, lines 40-42 of the revised manuscript)

We revised the sentence by adding medicine examples and references as follows.

“The use of therapeutic monoclonal antibodies (mAb) is becoming more common in the treatment of cancer (with drugs such as pembrolizumab, nivolumab, trastuzumab, and so on) and autoimmune diseases (with drugs such as adalimumab, infliximab, and so on) [1-7].”

**Specific Comment 5**

Revise following grammars; “Several grams/liter of production by recombinant Chinese hamster ovary (rCHO) cells, which harbor mAb genes”

**Response** (See page 3, lines 44-45 of the revised manuscript)

We revised the sentence as follows.

"MAb concentrations of several grams per liter, produced by using recombinant Chinese hamster ovary (rCHO) cells, which harbor genes encoding for mAb"

**Specific Comment 6**

What does mean the “state”?

**Response** (See page 3, lines 54 of the revised manuscript)

We thought the cell cycle explained “the state.” We revised this to “intracellular state.”

**Specific Comment 7**

How was structure-activity relationship evaluated? “The structure-activity relationship of MPPB was also evaluated.” is abstract.

**Response** (See page 4, lines 66-69 of the revised manuscript)

We agree that this sentence was abstract and should include a note on 2,5-dimethylpyrrole. The sentence was revised as follows.

“The structure-activity relationship of MPPB was also evaluated using the compounds corresponding to the partial structures of MPPB as additives in batch cultures and demonstrated that 2,5-dimethylpyrrole exhibited a strong enhancement of the cell-specific productivity of mAb.”

**Specific Comment 8**

Does “a target concentration” mean “a target viable cell concentration”?

**Response** (See page 5, lines 85 and 92, page 6, lines 106, and page 7, lines 115 of the revised manuscript)

Yes, it means “a target viable cell concentration.” We revised all relevant sentences to use this alternate terminology.

**Specific Comment 9**

Working volume of 50 mL in 125 mL or 50 mL of media and then seed culture? Which is correct?

**Response** (See page 5, lines 86-87, page 6, lines 108 and page 7 line 116 of the revised manuscript)

A working volume of 50 mL is correct. The related sentences were revised as follows.

“The working volume was adjusted to 50 mL in the 125-mL flasks and to 100 mL in the 250-mL flasks.” (Lines 86-87)

“The working volume was adjusted to 10 mL.” (Line 108)

“The working volume was adjusted to 50 mL.” (Line 116)

**Specific Comment 10**

Need more context. Is it (arbitrary unit) mab concentration or not?

**Response** (See page 6, lines 98-99 of the revised manuscript)

It was not the mAb concentration. This indicator was the arbitrary unit, which was converted from the mAb concentration. The following context was added to the manuscript.

“The resulting mAb concentrations were converted to arbitrary units according to the following equation (1)”

**Specific Comment 11**

Use format as equation 1 or list as text (- is minus or subtract from

**Response** (See page 6, lines 100-101 of the revised manuscript)

Based on the reviewer’s comment, we formatted equation (1) as follows.

**Specific Comment 12**

There was substantial grammatical issues, primarily in the abstract and introduction which will require a major revision. One particular issue in the results section is the use of glucose consumption and lactate accumulation. This terminology should be clarified to be either glucose concentration, cell-specific glucose uptake rate, or cumulative glucose consumption, where applicable.

**Response** (See lines 30, 65, 129, 253, 254, 256, 259, 265, 266, 287, 293, 295, 364, and 358-359; Figs 6, 8, and 10; and S1 Fig of the revised manuscript)

Based on the reviewer’s comment, we distinguished between “glucose concentration” and “cell-specific glucose uptake rate” and between “lactate concentration” and “cell-specific lactate production rate” in the relevant areas of the manuscript.

**Specific Comment 13**

Were they (5 compounds) replaced (with 67 compounds)? That would be a total of 67 compounds.

**Response** (See page 10, lines 176-178 of the revised manuscript)

Although 67 chemical compounds were commercially available, another 5 compounds for which the original structures were not commercially available were also tested as derivatives. Therefore, the total number of compounds was 72. Thank you very much for your suggestion about grammar. We revised the text as follows.

“Although 67 chemical compounds were commercially available, another 5 compounds for which the original structures were not commercially available were also tested as derivatives. Therefore, a total of 72 samples underwent the second screening in suspension culture.”

**Specific Comment 14**

This sentence is repetitive. Write a better transition.

“Relative cell-specific productivity, relative mAb concentration, and viability were compared with the control condition. For most of the compounds, the relative mAb concentration was increased with the chemical compound treatment. Therefore, the chemical compounds were screened for relative mAb concentration, relative cell-specific productivity, and viability.”

**Response** (See page 10, lines 186-187 of the revised manuscript)

We revised the sentence as follows.

“The relative cell-specific productivity, relative mAb concentration, and viability were assessed to evaluate the chemical compound treatments against the control condition.”

**Specific Comment 15**

Need revise the sentence like following;

“As ID number 62 demonstrated the largest effect as so was structure-activity relationship investigated further”

**Response** (See page 11, lines 201-202 of the revised manuscript)

Thank you very much for your suggestion for clarifying the sentence. Other factors were also tested in batch and fed-batch cultures. We therefore revised the sentence as follows.

“As ID number 62 demonstrated the largest effect, we further investigated the characteristics of ID number 62 in batch and fed-batch cultures.”

**Specific Comment 16**

Lactate is also double.

**Response** (See pages 14, lines 255-267 of the revised manuscript)

As you pointed out, lactate was also doubled. However, we would like to mention that the increase in the cell-specific lactate production rate was smaller than the increase in the cell-specific glucose uptake rate. This finding means that glucose was efficiently used in the tricarboxylic acid cycle. We revised the text as follows.

“In other words, the cell-specific glucose uptake rate under the MPPB-added condition was 0.61 pmol/cell/day higher than that under the control condition. Meanwhile, the cell-specific lactate production rate increased from 0.22 to 0.37 pmol/cell/day under the MPPB-added condition (Fig 8D). The increase in the cell-specific lactate production rate was 0.15 pmol/cell/day, which was smaller than the increase in the cell-specific glucose uptake rate (0.61 pmol/cell/day). This result suggested that consumed glucose was efficiently converted to ATP in the tricarboxylic acid cycle.”

**Specific Comment 17**

Combine with first sentence.

“The glucose consumption rate under the control condition was faster than that under the MPPB-added condition, as shown in Fig 9A. Residual glucose was fully consumed by day 10. On the other hand, glucose under the MPPB-added condition was retained until day 14. The lactate concentration was kept under 0.8 g/L, and no difference was noted in both conditions (Fig 9B). The specific glucose consumption rates were 0.63 and 0.74 pmol/cell/day under the control and MPPB-added conditions, respectively (Fig 9C).”

**Response** (See page 16, lines 286-290 of the revised manuscript)

Thank you for this suggestion. We revised the text as follows.

“Residual glucose was fully consumed by day 10 under the control condition, while glucose under the MPPB-added condition was retained until day 14 (Fig 10A). The cell-specific glucose uptake rates were 0.63 and 0.74 pmol/cell/day under the control and MPPB-added conditions, respectively (Fig 10C). The lactate concentration was kept under 0.8 g/L and no difference was noted in both condition (Fig 10B).”

**Specific Comment 18**

“Cell-specific glucose uptake rate (C) was indicated as a slope of consumed glucose concentration against integral viable cell concentration [28, 29].” This sentence should be noted earlier.

**Response** (See page 8-9, lines 149-154 and page 14, lines 250-251 of the revised manuscript)

Based on the reviewer’s comment, we added the calculation method to the Materials and Methods section. Similar calculation methods were used for cell-specific productivity and cell-specific lactate production rate. In addition, the written expressions in the manuscript were aligned. We updated the manuscript as follows.

“**Calculation of cell-specific productivity, cell-specific glucose uptake rates, and cell-specific lactate production rates**

Cell-specific productivity (pg/cell/day), cell-specific glucose uptake rates (pmol/cell/day), and cell-specific lactate production rates (pmol/cell/day) were calculated as the slopes of mAb concentration (mg/L), consumed glucose concentration (mM), and lactate concentration (mM), respectively, to integral viable cell concentration ((cells·day)/mL), according to previous reports [28, 29].” (Lines 151-154)

“The slopes of mAb concentration (**C**) to integral viable cell concentration was used to indicate cell-specific productivity (**D**) [28].” (Lines 250-251)

**Specific Comment 19**

You've demonstrated a chemical dose-dependent response to viability. Should mention that lower concentrations would need to be tested to understand potency each of the chemicals 2,5-dimethylpyrrole is most comparable to MPPB.

**Response** (See page 18, lines 331-336 of the revised manuscript)

Thank you for this important comment. We added new data about the relationships among some pyrroles’ concentrations, cell-specific productivity, and viability. The data showed that 2,5-dimethylpyrrole was the best, with viability above 80%, which is a realistic condition for cell cultures. In addition, 3,4-dimethylpyrrole had high cell-specific productivity. However, it had lower productivity than 2,5-dimethylpyrrole. We think the data support the conclusion that 2,5-dimethylpyrrole was the best compound. Therefore, the data were added to the Supporting Information (S2 Fig). Moreover, the following context was added to the manuscript.

“Thus, 2,5-dimethylpyrrole (13) was found to be the most effective chemical structure to increase cell-specific productivity while retaining viability in the 0.32 mM concentration. In addition, alkyl-pyrroles (9-14) that showed high cell-specific productivity in the 0.32 mM concentration were tested to clarify which pyrrole derivative was the best compound in terms of increasing cell-specific productivity and retaining viability. The results showed that 2,5-dimethylpyrrole (13) effectively increased cell-specific productivity while retaining viability (S2 Fig).”

**Specific Comment 20**

“Viability” would be correct, but not “extension of cell culture period”.

**Response** (See page 4, line 64 and page 21, lines 350 and 353-354 of the revised manuscript)

Thank you very much for this clarification. Retained viability contributed to extended cell culture periods. Therefore, “viability” is appropriate in this context. In all related sentences, we changed language reflecting “extension of cell culture period” to language reflecting “viability.”

**Specific Comment 21**

How much lower? Please clarify the value.

**Response** (See page 21, lines 357-358 of the revised manuscript)

Based on the reviewer’s comment, we clarified the value as follows.

“…viability under the control condition was 45% lower than that under the MPPB-added condition on day 12.”

**Specific Comment 22**

Unlikely... lower growth indicates cell cycle arrest. Cells consume less amino acids where growth is arrested. Higher cell density and increased growth would deplete component most likely.

**Response** (See page 21, lines 358-360 of the revised manuscript)

Based on the reviewer’s comment, we revised the sentence as follows.

“These results suggested that cell growth suppression by MPPB treatment prevented the depletion of medium components except for glucose, resulting in maintained viability.”

**Specific Comment 23**

This is also unlikely. “The cause of increased cell-specific productivity, however, is clearer. In our study, MPPB treatment had two important effects to increase cell-specific productivity. One was the suppression of cell growth (Figs 6A and 8A), and the other was the acceleration of specific glucose consumption. Both the effects would control the amount of intracellular adenosine triphosphates (ATPs)”

**Response** (See pages 21, lines 361-380 and Fig 6 of the revised manuscript)

We understand your concern. We included a new figure (Fig 6), mentioned in the Results section, that shows the relationship between the amount of intracellular ATP, the cell-specific glucose uptake rate, cell-specific productivity, and viable cell density (VCD). The figure shows that intracellular ATP amounts, cell-specific glucose uptake rates, and cell-specific productivity were completely positively correlated. On the other hand, intracellular ATP amounts and VCD had a negative correlation. These phenomena have been reported by Becker et al. and Hara and Kondo, who found that intracellular ATP amounts were positively correlated with cell-specific productivity (refs. 30 and 31 of the revised manuscript). Further, increased cell-specific glucose uptake rate and suppressed VCD have been shown to increase the amount of intracellular ATP (refs. 32-34 of the revised manuscript). Therefore, we think that one of the reasons for the increased productivity was the enhanced amount of intracellular ATP.

**Specific Comment 24**

Glucose is converted to pyruvate. Pyruvate is either converted to lactate or enters the TCA cycle.

**Response** (See pages 22, lines 366-368 and Fig 6 of the revised manuscript)

We added the following explanation.

“An increased cell-specific glucose uptake rate leads to an increased amounts of intracellular ATP when glucose is metabolized to ATP in the tricarboxylic acid cycle [32, 33], but not when glucose is metabolized to lactate.”

Our results showed that the cell-specific lactate production rate increase was lower than the cell-specific glucose uptake rate increase. (This result was also added to the revised manuscript [lines 253-261]). This finding means that most consumed glucose was changed to ATP. To support this explanation, the ATP amounts in the batch cultures were added as new data (Fig 6).

**Specific Comment 25**

Should be in the results section. Where is the method for this? (about ATP)

**Response** (See pages 8, lines 138-147; page 13, lines 227-235; page 22, lines 371-378; and Fig 6 of the revised manuscript)

We described the ATP analysis method in the Materials and Methods section (pages 8, lines 138-147). In addition, the content of Fig 6 is now reflected in the Results section (page 13, lines 227-235). Finally, the content of the Discussion section was revised (page 22, lines 371-378). The specific updates are as follows.

Materials and Methods section:

“**Measuring intracellular ATP**

An intracellular ATP assay kit (v2, Toyo B-Net, Tokyo, Japan) was used to quantitate ATP amounts in rCHO cells. Each cell culture solution (100 µL) was diluted with phosphate-buffered saline (1 mL) and then centrifuged (450 g, 5 min), and the supernatant was discarded (1 mL). The resulting cell pellet was suspended in phosphate-buffered saline (1 mL) and then centrifuged (450 g, 5 min), and the supernatant was again discarded (1 mL). The cell pellet was suspended in the ATP extraction reagent from the kit (0.5 mL) and incubated for 5 minutes to extract ATP from the cells. The suspension (10 µL) was mixed with luciferase luminescence assay reagents (100 µL), and measurements were obtained by using the Infinite M Plex plate reader (Tecan, Mannedorf, Switzerland). The resulting ATP concentration was converted to the amount of intracellular ATP per cell.”

Results section:

“Becker et al. [30] and Hara and Kondo [31] reported that increased amounts of intracellular ATP are associated with increased cell-specific productivity. Therefore, we investigated intracellular ATP amounts and cell-specific glucose uptake rates, which are related to ATP production [32, 33], in the batch cultures to clarify the reason for the increasing cell-specific productivity (Fig 6). The results showed that both intracellular ATP amounts and cell-specific glucose uptake rates were increased with higher MPPB concentrations. Intracellular ATP amounts increased from 8 to 24 fmol/cell (Fig 6A), and cell-specific glucose uptake rates increased from 0.9 to 2.6 pmol/cell/day (Fig 6B). Intracellular ATP amounts and cell-specific glucose uptake rates were positively correlated with cell-specific productivity (Fig 6C). Additionally, VCD was suppressed with MPPB treatment (Fig 6D)”

Discussion section:

“To test this hypothesis, we investigated the relationship between the amount of intracellular ATP and MPPB concentration (Fig 6). We found that the amount of intracellular ATP was enhanced with higher MPPB concentrations (Fig 6A). Cell-specific glucose uptake rates and cell-specific productivity were also increased (Figs 6B and 6C). On the other hand, VCD was also suppressed with higher MPPB conditions (Fig 6D). Therefore, we predicted that the increased cell-specific productivity under the MPPB-added condition might occur due to the increasing amount of intracellular ATP resulting from cell growth suppression and enhanced cell-specific glucose uptake rates.”

**Specific Comment 26**

How is MPPB structure similar to CDK4/6 inhibitor? What other hypothesis do you have for galactosylation suppression?

**Response**

Thank you for this question. MPPB and CDK4/6 are both alkaloids. However, their chemical structures are completely different. MPPB consists of succinimide, amino benzamide, and 2,5-dimethylpyrrole, while CDK4/6 consists of pyrimidine, 2-amino pyridine, piperazine, etc. We do not yet have any hypothesis regarding the galactosylation suppression. Therefore, we need to further study the mechanism for galactosylation suppression.

**Specific Comment 27**

Substantially and why is this? (The viability was decreased in the cases of other mono- or bis-alkyl-substituted pyrroles.)

**Response**

Although we considered the reason from the point of view of the electron density distribution of the chemical structure, the reason has not yet been clarified. As you mentioned, adequate concentrations exist for each compound (see S2 Fig of the revised manuscript). However, we cannot yet answer how the alkyl-substituted position affects viability. This mechanism requires further study.

**Specific Comment 28**

The discovered chemical additives should be compared to known additives that boost specific productivity as part of the structure-activity section.

**Response**

Thank you very much for this important suggestion. We also think that we should further the study in this way. We are proceeding with further study of MPPB and 2,5-dimethylpyrrole with comparisons to known additives. Therefore, in the structure-activity section of the present article, we would like to report only the chemical structure of MPPB and the structure-activity relationship of the most effective structure. We still believe the current content will be worthwhile for readers.

**Responses to Reviewer #2:**

Thank you very much for your essential questions, which we addressed to strengthen our study. The questions helped us to improve our manuscript and to focus the direction of our future research.

**Specific Comment 1**

Why does MPPB suppress cell growth and increase specific glucose consumption during monoclonal antibody production? Also, it also can suppress the galactosylation on a monoclonal antibody. Could you please explain why?

**Response**

This is a key question. We analyzed RNA sequences in a preliminary study, but differences between the control and MPPB-added conditions were not investigated. Therefore, we cannot provide an explanation based on the current data. We would like to clarify this characteristic as much as possible in future research.

**Specific Comment 2**

Could you please explain the concentration of MPPB on cells growth and monoclonal antibody production?

**Response** (See page 12, lines 217-221 and Fig 5D of the revised manuscript)

Thank you for pointing out the relationship between MPPB concentration and mAb production. MAb production decreased with higher MPPB concentrations due to cell growth suppression (see Fig 5D, which was newly added to the revised manuscript). We paid attention to increased cell-specific production and proceeded with studies in batch and fed-batch cultures. The following content was added to the revised manuscript based on your question.

“MAb concentration decreased with the addition of MPPB due to suppressed cell growth in these short-period batch cultures (Fig 5D). These results showed that treatment with MPPB concentrations of 0.32 to 0.64 mM suppressed cell growth and increased cell-specific productivity while retaining viability. Therefore, MPPB concentrations of 0.32 to 0.64 mM were used to characterize MPPB.”

**Specific Comment 3**

What is the difference of MPPB from any other known chemicals for monoclonal antibody production in Chinese hamster ovary cells?

**Response** (See page 22, lines 378-380 and 387 of the revised manuscript)

This compound would be the same as other compounds in terms of cell growth suppression and increased cell-specific productivity or mAb production. However, in most cases, the relationship between increased glucose consumption and increased intracellular ATP amounts is not reported or discussed (refs. 15-24 of the revised manuscript). This point will be new knowledge. Therefore, we added this information to the manuscript as follows.

“Our results showed that a chemical compound such as MPPB, which suppresses cell growth and increases intracellular ATP amounts, may improve mAb production in rCHO cell cultures.”

Further, valeric acid, valproic acid, and CDK4/6 inhibitor are known to increase the galactosylation ratio (refs. 18, 19 and 22 of the revised manuscript), an outcome that differs from that of MPPB. Therefore, MPPB would also have worth from the point of diversity as a galactosylation control reagent. Valeric acid and valproic acid are now mentioned in the revised manuscript to strengthen the difference between MPPB and other chemical compounds.

**Specific Comment 4**

The authors claim MPPB increases the production of ATP, which is responsible for the increase of monoclonal antibody productivity? The reviewer wonders whether ATP has the same effect on the monoclonal antibody productivity? Why?

**Response** (See page 13, lines 227-235 and Fig 6 of the revised manuscript)

CHO cells need ATP for mAb production. In addition, there are some reports about the relationship between mAb production and the amount of intracellular ATP (refs. 30 and 31 of the revised manuscript). To support this theory, we added new information to the revised manuscript. The data showed that mAb production per cell and intracellular ATP amounts are completely correlated (see Fig 6 of the revised manuscript).

**Specific Comment 5**

How does MPPB regulate the produce of ATP in the cells?

**Response** (See page 13, lines 227-235; page 22, lines 361-380; and Fig 6 of the revised manuscript)

The detailed mechanism is not yet clear. However, based on our results and past reports, we suspect that cell growth suppression and increased cell-specific glucose consumption without much lactate accumulation are critical reasons. The former saves ATP (ref. 34 of the revised manuscript), and the latter produces ATP (refs. 32 and 33 of the revised manuscript). We found that the amount of intracellular ATP changed depending on MPPB concentration (Fig 6). This content was added to the manuscript. We need further studies to investigate the mechanism.

**Specific Comment 6**

The authors suggest 2,5-dimethylpyrrole was the most effective partial structure of MPPB on monoclonal antibody production. Whether can 2,5-dimethylpyrrole affect monoclonal antibody production through affecting the production of ATP? Is this mechanism from other known chemicals used for monoclonal antibody production in the cells?

**Response**

Thank you for your comment. This point is very important. Our research showed that 2,5-dimethylpyrrole increased intracellular ATP amounts while increasing cell-specific glucose uptake rates. This research is ongoing. Therefore, in this manuscript, we would like to mention only that 2,5-dimethylpyrrole was the most effective partial structure of MPPB. In the near future, we would like to report this additional information.

**Other notes**

We adjusted some words for easier understanding by readers. We apologize for these revisions.

(See page 20, line 338 of the revised manuscript)

Specific productivity → Cell-specific productivity

(See page 20, Table 1 of the revised manuscript)

Relative specific production rate → Relative cell-specific productivity

(See page 21, lines 395, 396, and 402 of the revised manuscript)

2,5-dimethyl pyrrole → 2,5-dimethylpyrrole

(See page 21, lines 395 of the revised manuscript)

2,5-dimethyl pyrrolidine →2,5-dimethylpyrrolidine

Again, thank you for giving us the opportunity to strengthen our manuscript by addressing your comments and questions. We incorporated your feedback. We appreciate your time and look forward to your response.

Sincerely,

Yuichi Aki

Biologics Technology Research Laboratories, Biologics Division, Daiichi Sankyo Co., Ltd.

2716-1, Aza Kurakake, Oaza Akaiwa, Chiyoda-machi, Oura-gun, Gunma 370-0503, Japan

E-mail: aki.yuichi.ha@daiichisankyo.co.jp
