# Supplementary material for: 4-(2,5-Dimethyl-1H-pyrrol-1-yl)-N-(2,5-dioxopyrrolidin-1-yl) benzamide improves monoclonal antibody production in a Chinese hamster ovary cell culture
Source: PLoS One. 2021 Apr 22;16(4):e0250416. doi: 10.1371/journal.pone.0250416 (PMC8061942; doi:10.1371/journal.pone.0250416)
Supplement: Attachment — Submitted filename: Responses to Reviewers.docx [file pone.0250416.s008.docx]

**Responses to Reviewer #1:**

Thank you again for your advice, which was very insightful. We sincerely considered your advice and updated our manuscript to reflect as much of your advice as possible. Some of the sentences you pointed out were very important to our manuscript. We hope our responses below acceptably address your comments and questions.

**Specific Comment 1**

Revise as follows. From “Some chemical compounds such as dimethyl sulfoxide, lithium chloride and butyric acid, and so on, have been shown...” to “Chemical compounds, such as dimethyl sulfoxide, lithium chloride and butyric acid, have been shown...”

**Response** (See page 2, lines 24-25 of the revised manuscript)

The sentence was revised as you suggested.

**Specific Comment 2**

Revise as follows. From “these” to ”mammalian”

**Response** (See page 2, line 25 of the revised manuscript)

The sentence was revised as you suggested.

**Specific Comment 3**

Revise as follows. “The compound has characteristics that suppress cell growth” to "The compound suppressed cell growth"

**Response** (See page 2, line 29 of the revised manuscript)

The sentence was revised as you suggested.

**Specific Comment 4**

Revise as follows. “increase cell-specific glucose uptake rate and amount of” to "increased both cell-specific glucose uptake rate and the amount of...."

**Response** (See page 2, lines 29-30 of the revised manuscript)

The grammar of this sentence was revised following your suggestion.

**Specific Comment 5**

Revise as follows. Erase “with drugs such as” and revise from “and so on” to “etc.”

**Response** (See page 3, lines 41 of the revised manuscript)

The sentence was revised as you suggested.

**Specific Comment 6**

Revise as follows. From “cells, which harbor genes encoding” to "cells harboring the genes encoding..."

**Response** (See page 3, line 45 of the revised manuscript)

The grammar of this sentence was revised following your suggestion.

**Specific Comment 7**

Revise as follows. From “culture parameter” to ", and culture parameter...” and erase “and so on”

**Response** (See page 3, line 47 of the revised manuscript)

The sentence was revised as you suggested.

**Specific Comment 8**

Revise as follows. Start new paragraph here “Efforts have…” and “To pursue the possibility…”

**Response** (See page 3, line 48 and page 4, lines57 of the revised manuscript)

These sentences were revised as you suggested.

**Specific Comment 9**

Revise as follows. From “MPPB-added” to "MPPB-supplemented"

**Response** (See page 4, line 63 of the revised manuscript)

The sentence was revised as you suggested.

**Specific Comment 10**

Did improving viability significantly improve mAb production or was it worth including because other supplemented molecules decreased viability?

Suggested improvement to sentence: The results showed MPPB improved mAb production while retaining viability by suppressing cell growth and increasing the cell-specific glucose uptake rate and intracellular adenosine triphosphate (ATP).

**Response** (See page 4, lines 64-67 of the revised manuscript)

Thank you very much for this insight. Retained viability also contributed to prolong production periods. If viability was not retained, mAb production was lower in the MPPB-supplemented condition than in the control condition, despite the increasing cell-specific production rate. Therefore, it was considered one of the reasons for the increase in mAb production. Of course, another reason is the increase in cell-specific productivity. We revised this sentence in light of your suggestion, as follows.

“The results showed that MPPB improved mAb production while retaining viability and increasing cell-specific productivity by suppressing cell growth and increasing the cell-specific glucose uptake rate and the amount of intracellular adenosine triphosphate (ATP)”

**Specific Comment 11**

Revise as follows.

Erase “it”, Revise as follows. From “The structure-activity relationship of MPPB was also evaluated using the compounds corresponding to the partial structures of MPPB as additives in batch cultures and demonstrated that 2,5-dimethylpyrrole exhibited a strong enhancement of the cell-specific productivity of mAb.” to “The structure-activity relationship of MPPB was evaluated using the compounds corresponding to the partial structures of MPPB as additives in batch cultures and demonstrated that 2,5-dimethylpyrrole enhanced the cell-specific productivity.”

**Response** (See page 4, line 67-70 of the revised manuscript)

The grammar of this sentence was checked and revised per your suggestion.

**Specific Comment 12**

Sentence is confusing. Culture solutions usually means basal media or feeds i.e. no cells. Static culture is usually for adherent cells

**Response-1** (See page 6, line 109 of the revised manuscript)

Thank you very much for this note. “Cell culture solutions” is correct. Therefore, we changed “culture solutions” to “cell culture solutions.”

**Response-2** (See pages 5-7, lines 92-117 and pages 10-11, lines 183, 188, and 189 of the revised manuscript)

We selected static culture in the second screening because the results of the second screening were similar to those of the initial screening in static culture. We estimated that the cell culture in the 96-well plate was substantially similar to that of the static culture condition. Our original explanations in the Materials and Methods section and the Results section were confusing. We thus clarified the culture method in the Materials and Methods section, as follows. We also explained the initial and second screenings in the screening sections. The second screening section was transferred from the batch culture section. We very much appreciate your comment leading to this revision.

“**Screening of the chemical compound library**

**Initial screening**

The rCHO cells were…

**Second screening**

In the second screening to evaluate…

**Batch culture condition**

In the suspension batch cultures…”

In the Results section, we erased the words “in suspension,” “in a suspension culture,” and “in a suspension culture for 3 days,” as they were thought to confuse readers. Plus, this idea was already explained in the Materials and Methods section.

**Specific Comment 13**

I believe this section should be merged with the "Second screening of the selected compounds in a suspension culture" as I believe it describes the same experimental results. If this section presents data from a separate experiment, it should be made clear.

**Response** (See page 11, lines 201-204 of the revised manuscript)

This selection was highly important for our study. Therefore, we re-tested the selected compounds to confirm the reproducibility of second screening results. We added a new explanation (as follows), and we changed the “MPPB as an effective additive of mAb production” section from a level 2 heading to a level 3 heading. Therefore, the “MPPB as an effective additive of mAb production” section is now included in the “Second screening of the selected compounds” section.

"The samples with the ID numbers 42, 62, and 67 were re-evaluated to confirm the reproducibility of second screening results. The three selected compounds had higher relative mAb concentration and relative cell-specific productivity."

**Specific Comment 14**

Is the data on this graph the same as what was shown in Fig. 2? If so, choose one figure to display this info.

**Response**

This answer is the same as the one for “Specific Comment 13.” Therefore, we decided to keep this figure.

**Specific Comment 15**

Revise as follows. From “MAb concentration decreased with the addition of MPPB due to suppressed cell growth in these short-period batch cultures” to " A dose-dependent reduction in day 4 mAb concentration was observed with increasing concentrations of MPPB due to suppressed cell growth in these short-period batch cultures"

**Response** (See page 12, lines 222-224 of the revised manuscript)

The substance of this sentence was revised following your suggestion.

**Specific Comment 16**

Revise as follows. From “and cell specific productivity (**C**), and mAb concentration (**D**) were evaluated on day 4.” to " and day 4 cell-specific productivity (**C**), and day 4 mAb concentration (**D**) were evaluated."

**Response** (See page 13, lines 229-231 of the revised manuscript)

The sentence was revised as you suggested as follows.

“The tested MPPB concentrations are indicated in the figure. In these tests, viable cell density (**A**), viability (**B**), cell-specific productivity on day 4 (**C**), mAb concentration on day 4 (**D**) were evaluated.”

**Specific Comment 17**

Cell growth or integral viable cell density was suppressed with MPPB treatment.

**Response** (See page 13, lines 241 and 244 and page 22, line 389 of the revised manuscript)

We agree with your comment that “suppression” should be used for cell growth, while “decrease” should be used for VCD. Please see the following specific adjustments.

"VCD was suppressed" → "cell growth was suppressed"

"suppressed VCD" → "suppressed cell growth"

"VCD was also suppressed" → "cell growth was also suppressed"

**Specific Comment 18**

Revise as follows. From “there was longer viability under the MPPB-added condition.” to "The MPPB-added condition retained a higher viability."

**Response** (See page 14, line 252 of the revised manuscript)

The sentence was revised as you suggested.

**Specific Comment 19**

Erase "In other words, the cell-specific glucose uptake rate under the MPPB-added condition was 0.61 pmol/cell/day higher than that under the control condition."

**Response** (See page 15 of the revised manuscript)

The sentence was erased as you suggested.

**Specific Comment 20**

Revise as follows. From “This result suggested that consumed glucose was efficiently converted to ATP in the tricarboxylic acid cycle.” to "This result suggests that consumed glucose was more efficiently converted to ATP via the tricarboxylic acid (TCA) cycle in the MPPB-added condition."

**Response** (See page 15, lines 267-269 of the revised manuscript)

The sentence was revised as you suggested.

**Specific Comment 21**

Revise as follows. From “kept” to "maintained"

**Response** (See page 16, line 283 of the revised manuscript)

The word was revised as you suggested.

**Specific Comment 22**

Revise as follows. From “Despite suppression of the VCD” to "Despite lower VCD"

**Response** (See page 16, line 334 of the revised manuscript)

The sentence was revised according to your suggestion as follows.

**Specific Comment 23**

Revise as follows. From “also became” to "was also"

**Response** (See page 16, line 286 of the revised manuscript)

The word was revised as you suggested.

**Specific Comment 24**

This should be mentioned earlier that “Residual glucose was fully consumed by day 10 under the control condition, while glucose under the MPPB-added condition was retained until day 14 (Fig 10A)”. What impact did glucose depletion have on the culture and on the results?

**Response** (See page 14, lines 248-250 and page 15, lines 278-281 of the revised manuscript)

Glucose depletion shortened the culture periods. The results showed that mAb production with glucose depletion was lower than when glucose was retained. However, mAb production was highest in the MPPB(+) condition without additional glucose. Therefore, “the results are consistent with those of the current manuscript.”

The representative data is the MPPB(+) condition without additional glucose, and the control condition is the MPPB(-) condition without additional glucose. The nutrients are completely the same in both conditions. The conditions clarify only the effect of MPPB.

We added the following sentences to explain that we did not optimize the cell culture conditions in order to clarify the effect of MPPB.

“These cell cultures were continued until less than 70% viability was reached. Culture conditions including pH, glucose concentration, and aeration were not optimized in any cell culture at this time in order to evaluate the effect of MPPB only.”

“These cell cultures were continued until less than 70% viability was reached. Culture conditions (pH, glucose concentration, and aeration) were not optimized in any cell culture at this time in order to evaluate the effect of MPPB only.”

**Specific Comment 25**

What about cell-specific lactate production? It appears that the MPPB (+) fed-batch culture has higher lactate production per cell which is the opposite of the batch process. A hypothesis for why this is should be mentioned?

**Response** (See page 16, lines 299-300, page 17, lines 304-307, and page 22, line 377 of the revised manuscript)

The lactate concentration looks high in the MPPB (+) condition. However, the cell-specific lactate production rate was lower with the MPPB treatment. Each value was -0.057 pmol/cell/day in the MPPB (+) condition and -0.003 pmol/cell/day in the MPPB (-) condition). We added this data as Fig 10D, and the following sentences were added to the manuscript.

“The lactate concentration was kept under 0.8 g/L, and the cell-specific lactate production rates were similar in both conditions (Figs 10B and 10D).”

“Cell-specific glucose uptake rates (**C**) and cell-specific lactate production rates (**D**) were indicated as the slopes of consumed glucose concentration and lactate concentration, respectively, against integral viable cell concentration [28, 29].”

“Figs 8D and 10D”

**Specific Comment 26**

What day was the MPPB (-) condition analyzed?

**Response** (See page 17, lines 309-310 of the revised manuscript)

Thank you for making this point. The MPPB (-) condition was analyzed on day 10. All N-glycans were analyzed on the final culture day. Therefore, we revised the text as follows.

" In addition, the major *N*-linked glycans (G0F, G1F, G2F, and M5) of mAb were investigated on each final culture day (MPPB (+): day 14, MPPB (-): day 10). "

**Specific Comment 27**

Divided based on expected active sites?

**Response** (See page 17, lines 318 of the revised manuscript)

No, we did not use any prediction tool, when it came to the structure-activity relationship analysis. Therefore, we split the MPPB based on commercial availability and inspiration.

**Specific Comment 28**

in batch or fed-batch?

**Response** (See page 18, lines 324-325 of the revised manuscript)

This study was executed in batch cultures. Therefore, we revised as follows.

"The activity of the five chemical components derived from MPPB was identified and compared with that of MPPB in terms of cell-specific productivity in the batch cultures"

**Specific Comment 29**

What is the impact of this result in terms of structure and activity?

**Response** (See page 18, line 328 of the revised manuscript)

As you mentioned, we judged 2,5-dimethylpyrrole to be the most active structure due to this result. Therefore, we added the following sentence to the manuscript.

"These results suggested that 2,5-dimethylpyrrole was the most active chemical structure of MPPB."

**Specific Comment 30**

Please add these labels (2), (3), etc. to the structures in figure 12. i.e. MPPB (2)

**Response** (See Fig 12 of the revised manuscript)

We revised the labels in Fig 12 as you suggested.

"MPPB (2), etc."

**Specific Comment 31**

Revise as follows. From “affect” to "effect"

**Response** (See page 19, line 343 of the revised manuscript)

We re-considered which word—“affect” of “effect”—would be most adequate for our manuscript. We would like to select “affect” in this sentence.

**Specific Comment 32**

Were tested at various concentrations (0.08-0.64 mM) in batch cultures? This sentence should go before the sentence "Thus, 2,5-dimethylpyrrole (13) was found..."

**Response** (See page 19, lines 343-345 of the revised manuscript)

To reflect your previous suggestion, we had newly studied at various concentrations (0.08–0.64 mM) in batch cultures (S2 Fig). We also think this data is useful, but our results are not affected by the information presented in S2 Fig. Therefore, we would like to handle it as supporting information for the simplicity of the manuscript.

We moved this sentence to the place where you suggested, and we revised some points, as follows.

"In addition, the alkyl pyrroles (**9**-**14**) that showed high cell-specific productivity in the 0.32 mM concentration were also tested in other MPPB concentrations (S2 Fig). The results showed that 2,5-dimethylpyrrole (**13**) effectively increased cell-specific productivity without decreasing viability."

**Specific Comment 33**

Alkyl-pyrroles or alkyl pyrroles? be consistent.

**Response** (See page 19, line 343 of the revised manuscript)

We have ensured “alkyl pyrroles” is used consistently.

**Specific Comment 34**

This sentence ” The results showed that 2,5-dimethylpyrrole (**13**) effectively increased cell-specific productivity without decreasing viability.” is not necessary. It is the same as the sentence two sentences before.

**Response** (See page 19, line 344 of the revised manuscript)

We would like to leave this sentence. Because this sentence became necessary according to our sentence revision.

**Specific Comment 35**

A diagram similar to figure 12 would be useful in understanding how the molecules differ.

**Response** (See page 20, lines 349-350 of the revised manuscript)

We added Fig 14 (the list of pyrrole derivatives), along with the following caption, according to your suggestion.

“Fig 14. Additional pyrrole derivatives tested to identify the structure-activity relationship of MPPB with viability and relative cell-specific productivity in rCHO cell cultures.”

**Specific Comment 36**

Revise as “MPPB: Retaining high viability (Figs 7B and 9B), and increasing of cell-specific productivity (Figs 7D and 9D).”

**Response** (See page 20, line 362-363 of the revised manuscript)

We revised this sentence following your suggestion.

"…MPPB: retaining high viability (Figs 7B and 9B), and increasing of cell-specific productivity (Figs 7D and 9D).

**Specific Comment 37**

Does viability significantly contribute to mAb production?

**Response** (See page 20, line 363 of the revised manuscript)

Yes, we think retained viability contributes to higher total mAb production.

For example, mAb production on day 8 in the batch cultures was lower in the MPPB(+) condition than in the MPPB(-) condition. However, mAb production became higher in the MPPB (+) condition on day 10. If viability was not retained until day 10, then the total mAb production would be lower in the MPPB (+) condition despite increasing cell-specific productivity with MPPB treatment.

**Specific Comment 38**

The section and the graph show glucose depletion on day 10, but the S1 fig shows it was maintained. This should really be in the discussion about the results for section "Effect of MPPB on the culture profile in the fed-batch cultures". If these studies are similar, choose the most representative study to present the data clearly. What about titer in the S1 fig?

**Response** (See page 21, line 369 and page 32, lines 565-571 [S1 Fig] of the revised manuscript)

The most representative data are the current data in the manuscript. Among all the data, the MPPB (+)/Gluc(-) condition was the highest in terms of mAb production. In addition, the preferable condition to compare with the MPPB(+)/Gluc(-) condition was the MPPB(-)/Gluc(-) condition because the nutrients were completely the same between both tests. This comparison made the effect of MPPB clearer.

We added mAb production, cell-specific glucose uptake rates, and cell-specific lactate production rates to S1 Fig. MAb production in the MPPB(+)/Gluc(+) condition decreased more than in the MPPB(+)/Gluc(-) condition. The reason for this has not yet been identified. Of course, cell-specific productivity and cell-specific glucose uptake rates were increased compared to the control in all MPPB-supplemented conditions.

"… and mAb concentration (**E**) were measured every 2 days. The each slope of mAb concentration, consumed glucose concentration and lactate concentration to integral viable cell concentration were used to indicate cell-specific productivity (**F**), cell-specific glucose uptake rates (**G**), and cell-specific lactate production rates (**H**), respectively [28]."

**Specific Comment 39**

Revise as follows. From “ATPs” to "ATP"

**Response** (See page 22, lines 379 of the revised manuscript)

The word was revised as you suggested.

**Specific Comment 40**

Reference: A Peak Antibody Production is Associated With Increased Oxidative Metabolism in an Industrially Relevant Fed-Batch CHO Cell Culture Neil Templeton, 1 Jason Dean, 2 Pranhitha Reddy, 2 Jamey D. Young

Growth-arrested cells divert glucose to TCA cycle

**Response** (See page 22, lines 379-380 and 384-385 and page 30, lines 530-532 of the revised manuscript)

Thank you very much for suggesting this paper. We did not notice that cell growth suppression was equal to induce cell into the stationary phase. According to your suggestion and this paper, we added the following text to the manuscript. It is a common idea according to past reports that cell growth suppression and increasing cell-specific glucose uptake rates both increase the amount of ATP. Therefore, we limited our hypothesis to only the MPPB-supplemented condition.

"Templeton et al. [35] reported that the stationary phase, which is a suppressed state of cell growth, activates the TCA cycle."

“it may be hypothesized that rCHO cells increased intracellular ATP amounts by suppressing cell growth and increasing cell-specific glucose uptake rates in MPPB-supplemented condition”

"35. Templeton N, Dean J, Reddy P, Young JD. Peak antibody production is associated with increased oxidative metabolism in an industrially relevant fed-batch CHO cell culture. Biotechnol Bioeng. 2013 Jul;110(7):2013-24. doi: 10.1002/bit.24858."

**Specific Comment 41**

This figure (S2 Fig) is very useful. Would 3,4-dimethylpyrole not be an example of a more potent inhibitor than 2,5-dimethylpyrrole as to 0.08 mM concentration is similar to the 0.64 mM concentration of 2,5-dimethylpyrrole?

**Response** (See page 32, lines 572 of the revised manuscript)

We agree that the cell-specific productivity of the 0.08 mM 3,4-dimethyl pyrrole treatment was similar to that of the 0.64 mM 2,5-dimethylpyrrole treatment. However, the trend of viability of 3,4-dimethyl pyrrole was not better than that of 2,5-dimethylpyrrole. Therefore, 2,5-dimethylpyrrole was considered suitable for cell culture.

According to these results, we think that the symmetric 2,5-dialkylated pyrrole is very important for retaining viability and increasing cell-specific productivity. This point will be clarified in a future study.

**Others**

(See page 16, lines 285 of the revised manuscript)

“times” was aligned as “-folds”.

(See page 22, lines 378 of the revised manuscript)

Reference position was moved as follows.

“ATP than quiescent cells [34]” to “Mulukutla et al. [34], growing”.

(See page 22, lines 383 of the revised manuscript)

“have reported” was revised as “reported”.

(See page 22, lines 390 of the revised manuscript)

“conditions” was aligned as “concentrations”.

(See page 23, lines 408 of the revised manuscript)

“viability and improve cell-specific productivity” was revised as “the viability and improve the cell-specific productivity”.

(See page 23, lines 408 of the revised manuscript)

“is” was revised as “was”.

Again, thank you for giving us the opportunity to strengthen our manuscript by addressing your comments and questions. We have incorporated your feedback. We appreciate your time and look forward to your response.

Sincerely,

Yuichi Aki

Biologics Technology Research Laboratories, Biologics Division, Daiichi Sankyo Co., Ltd.

2716-1, Aza Kurakake, Oaza Akaiwa, Chiyoda-machi, Oura-gun, Gunma 370-0503, Japan

E-mail: aki.yuichi.ha@daiichisankyo.co.jp
